# Acyl pyrazole sulfonamides as new antidiabetic agents: synthesis, glucosidase inhibition studies, and molecular docking analysis

**DOI:** 10.3389/fchem.2024.1380523

**Published:** 2024-04-17

**Authors:** Atteeque Ahmed, Sumera Zaib, Mashooq Ahmad Bhat, Aamer Saeed, Muhammad Zain Altaf, Fatima Tuz Zahra, Ghulam Shabir, Nehal Rana, Imtiaz Khan

**Affiliations:** ^1^ Department of Chemistry, Quaid-i-Azam University, Islamabad, Pakistan; ^2^ Department of Basic and Applied Chemistry, Faculty of Science and Technology, University of Central Punjab, Lahore, Pakistan; ^3^ Department of Pharmaceutical Chemistry, College of Pharmacy, King Saud University, Riyadh, Saudi Arabia; ^4^ Department of Chemistry and Manchester Institute of Biotechnology, The University of Manchester, Manchester, United Kingdom

**Keywords:** alpha glucosidase, diabetes mellitus, pyrazole-clubbed sulfonamides, pharmacokinetics, SeeSAR

## Abstract

Diabetes mellitus is a multi-systematic chronic metabolic disorder and life-threatening disease resulting from impaired glucose homeostasis. The inhibition of glucosidase, particularly *α*-glucosidase, could serve as an effective methodology in treating diabetes. Attributed to the catalytic function of glucosidase, the present research focuses on the synthesis of sulfonamide-based acyl pyrazoles **(5a-k)** followed by their *in vitro* and *in silico* screening against *α*-glucosidase. The envisaged structures of prepared compounds were confirmed through NMR and FTIR spectroscopy and mass spectrometry. All compounds were found to be more potent against *α*-glucosidase than the standard drug, acarbose (IC_50_ = 35.1 ± 0.14 *µ*M), with IC_50_ values ranging from 1.13 to 28.27 *µ*M. However, compound **5a** displayed the highest anti-diabetic activity (IC_50_ = 1.13 ± 0.06 *µ*M). Furthermore, *in silico* studies revealed the intermolecular interactions of most potent compounds (**5a** and **5b**), with active site residues reflecting the importance of pyrazole and sulfonamide moieties. This interaction pattern clearly manifests various structure–activity relationships, while the docking results correspond to the IC_50_ values of tested compounds. Hence, recent investigation reveals the medicinal significance of sulfonamide-clubbed pyrazole derivatives as prospective therapeutic candidates for treating type 2 diabetes mellitus (T2DM).

## Graphical Abstract

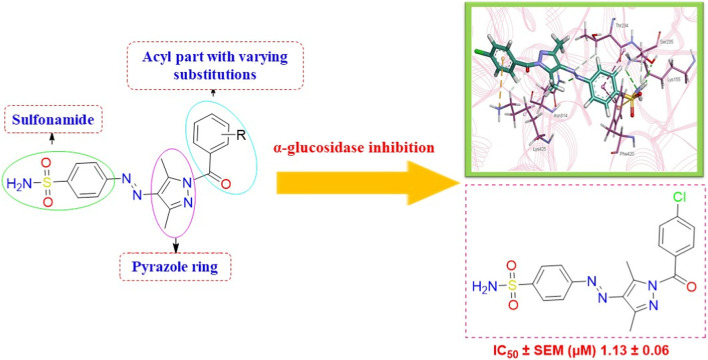

## 1 Introduction

Heterocyclic compounds generally hold a significant position in medicinal and organic chemistry with their diverse range of biological and pharmacological properties (Ansari and Lal, 2009; Shiro et al., 2015; Akhtar et al., 2017; Kakkar and Narasimhan, 2019). Owing to their importance, they contribute considerably to the development of potent and therapeutic drugs. Among the heterocyclic compounds that contain nitrogen and sulfur, sulfonamide derivatives with a pyrazole moiety hold a privileged position in organic chemistry (Kołaczek et al., 2014; Khan et al., 2016; Dhameja and Gupta, 2019; Faisal et al., 2019). They not only act as anticonvulsants (Abdul-Gafoor, 2016; Abdelgawad et al., 2018; Mishra et al., 2018), anti-stress and anxiogenics (Channar et al., 2018; Saha and Pal, 2020), antitumor (Channar et al., 2018), antiviral (Badampudi et al., 2014; Kołaczek et al., 2014; Kumar et al., 2015; Mustafa et al., 2022), antimicrobial, anticholinesterase (Mumtaz et al., 2019; Ghosh et al., 2020; Verma et al., 2020), antiulcer (Khan et al., 2018), and antimalarial (Thillainayagam et al., 2020) but also exhibit a variety of enzyme inhibition properties (Mumtaz et al., 2019; Bayrak, 2022; Chalkha et al., 2022; Mustafa et al., 2022). Additionally, some of the pyrazole-containing sulfonamide derivatives have been tested against *α*-glucosidase (Shu et al., 2016; Azimi et al., 2021). Others were found to be effective inhibitors of acetylcholinesterase, *β*-amyloid precursor protein cleavage enzyme 1 (BACE-1), and other metabolic enzymes (Tugrak et al., 2021; Gehlot et al., 2022).

Controlling diabetes is one of the greatest challenges of the 21st century (Kharroubi and Darwish, 2015; Bellary et al., 2021). Diabetes mellitus (DM), a multi-factorial syndrome, is a leading illness globally, and its prevalence is expected to double by 2030, with developing nations experiencing a 69% increase and industrialized countries a 20% increase. Due to being an endocrine disorder, diabetes is characterized by high blood glucose levels (hyperglycemia) (Kharroubi and Darwish, 2015; Bellary et al., 2021). These rising levels have been linked to serious health problems like cardiovascular diseases (O’gara et al., 2013; Kobayashi and Liang, 2015; Zhao et al., 2021), nephropathy (Mora-Fernández et al., 2014), retinopathy (Gispen and Biessels, 2000; Mensah and Kohner, 2002), encephalopathy (Veves and Malik, 2007), thrombosis (Vazzana et al., 2012), and Alzheimer’s disease (Kong et al., 2020). This is thus referred to as the *α*-Federation (IDF), with diabetes and its complications accounting for millions of deaths annually (Rogach et al., 2021). One of the most effective treatments for diabetics with postprandial hyperglycemia is to prevent the digestion of dietary carbohydrates (Tucci et al., 2010).

*β*-glucosidase is an enzyme that catalyzes the hydrolysis of cellobiose’s glycosidic bonds to produce glucose in the digestive system (Kumar et al., 2015). Similarly, *α*-glucosidase converts starch and other dietary carbohydrates into glucose (Joshi et al., 2015). After that, the breakdown of carbohydrates releases glucose into the bloodstream, causing hyperglycemia (Tucci et al., 2010; Joshi et al., 2015; Kumar et al., 2015). Consequently, the inhibition of *α*- and *β*-glucosidase enzymes can suppress the digestion of carbohydrates, causing a delay in the absorption of glucose and resulting in decreased blood sugar levels (Tucci et al., 2010; Joshi et al., 2015; Kumar et al., 2015). Nevertheless, drugs like acarbose, voglibose, and miglitol inhibit the glucosidase enzyme, although it is commonly reported that they have undesirable side effects such as diarrhea, abdominal pain, bloating, and flatulence (Lee et al., 2007; Fang et al., 2010; Derosa and Maffioli, 2012). Moreover, these drugs have been proven to reduce their effectiveness over time; hence, new and improved *α*- and *β*-glucosidase inhibitors are highly desired.

**FIGURE 1:**
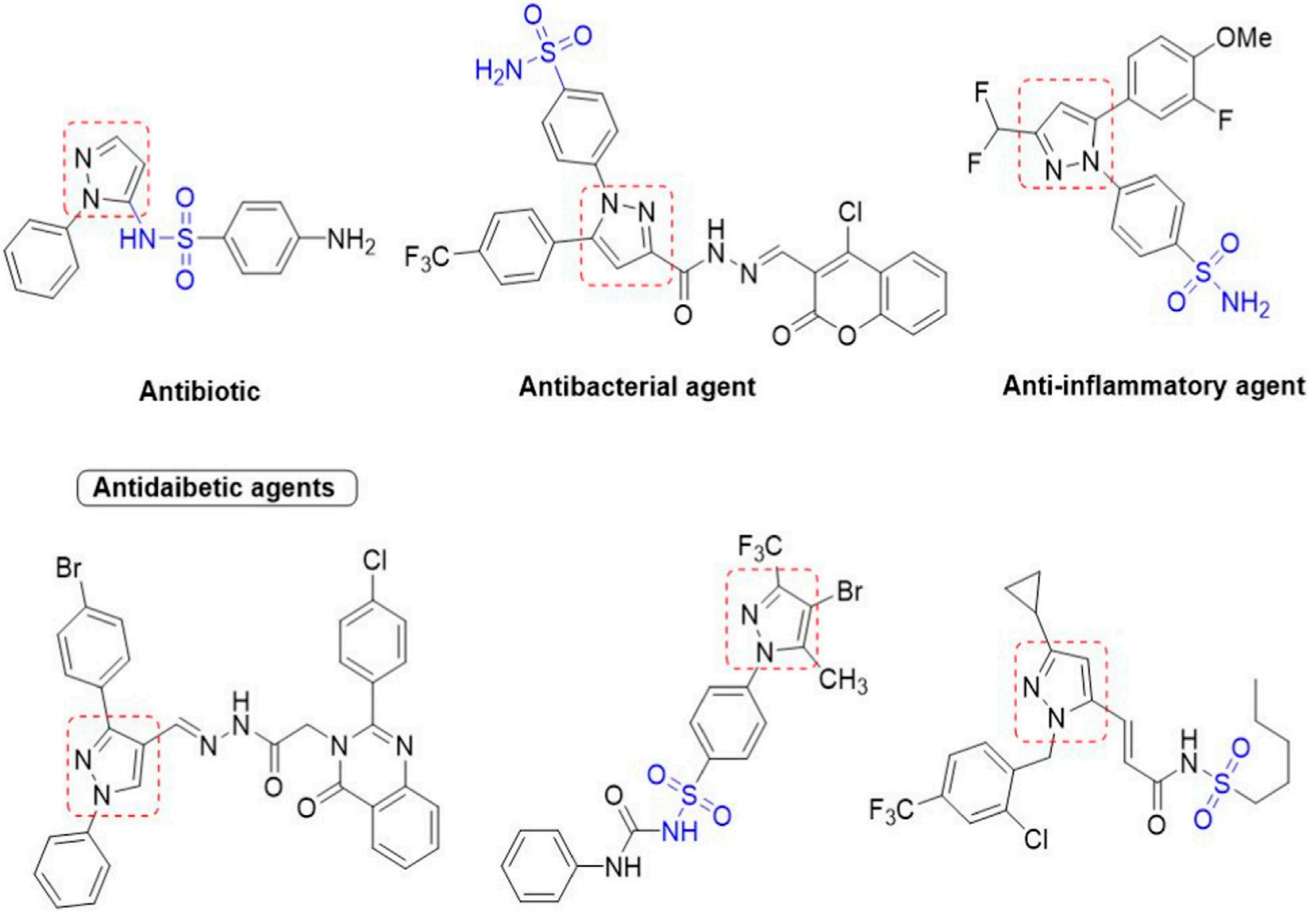
Sulfonamide compounds incorporating pyrazole with biological activities.

We here report a cheap and easy method for the synthesis of a novel series of sulfonamides linked with the pyrazole nucleus. Pyrazole and sulfonamides as individual moieties have a broad biological profile, including glucosidase inhibition. Various pyrazole-based sulfonamide compounds have been published with potent biological activities (Figure 1) (Dhameja and Gupta, 2019; Kausar et al., 2021; Ebenezer et al., 2022). Hence, considering their medicinal significance, we designed hybrid chemical entities by combining two different pharmacophoric cores in a single molecular architecture to address the health-related concerns and challenges in modern organic chemistry while also providing easy access to potent *α*-glucosidase inhibitors. To investigate the biological profile of the synthesized molecules, glucosidase inhibition analysis was conducted, and the data acquired were addressed using molecular docking analysis.

## 2 Material and methods

### 2.1 Experimental

#### 2.1.1 Procedure for the synthesis of acyl pyrazole sulfonamides (5a-j)

Sulfanilamide **1** was coupled with active methylene of acetyl acetone **2** by diazotization reaction to obtain intermediate **3**, which was further cyclized with different substituted phenyl hydrazides **4a-j** in ethanol for 30 min in the presence of few drops of conc. hydrochloric acid as a catalyst to furnish the final product, **5a-j**. The precipitated products were filtered off and washed with ethanol.

#### 2.1.2 (*E*)-4-((2,4-Dioxopentan-3-yl)diazenyl)benzenesulfonamide (3)

Yellow solid; yield: 91%; M.P.: 172 °C–174 °C; ^1^ **H NMR** (400 MHz, DMSO-*d*_*6*_) *δ* 13.66 (s, 1H, NH), 7.87–7.80 (m, 2H, Ar-H), 7.74–7.68 (m, 2H, Ar-H), 7.34 (s, 2H, NH_2_), 2.46 (s, 6H, 2CH_3_); ^**13**^ **C NMR** (101 MHz, DMSO-*d*_*6*_) *δ* 197.2, 196.3, 144.5, 139.7, 134.9, 127.2, 116.0, 31.2, 26.3.

#### 2.1.3 (*E*)-4-((1-(4-Chlorobenzoyl)-3,5-dimethyl-1*H*-pyrazol-4-yl)diazenyl)benzenesulfonamide (5a)

Yellow solid; yield: 86%; M.P.: 225 °C–227 °C; FT-IR (ATR) cm^-1^, 3366, 3207 (H–N, amine), 2986, 2958 (H–C, CH_3_), 1,693 (C=O, amide), 1,574 (C=C, Ar), 1,346 (S=O, sulfonamide), 1,290 (C-N); ^**1**^ **H NMR** (400 MHz, DMSO-*d*_*6*_) *δ* 8.05–7.97 (m, 4H, Ar-H), 7.97–7.91 (m, 2H, Ar-H), 7.68–7.62 (m, 2H, Ar-H), 7.53 (s, 2H, NH_2_), 2.98 (s, 3H, CH_3_), 2.44 (s, 3H, CH_3_); ^**13**^ **C NMR** (101 MHz, DMSO-*d*_*6*_) *δ* 167.13, 154.04, 147.03, 145.41, 144.17, 137.86, 137.11, 132.91, 131.08, 128.13, 127.00, 122.38, 14.86, 11.94; ESI-MS calc. for [C_18_H_16_ClN_5_O_3_S + H]^+^, m/z 417.0662; found, m/z 418.2 [M + H]^+^; RT = 2.973; purity: 95.10%.

#### 2.1.4 (*E*)-4-((1-(3-Chlorobenzoyl)-3,5-dimethyl-1*H*-pyrazol-4-yl)diazenyl)benzenesulfonamide (5b)

Yellow solid; yield: 82%; M.P.: 214 °C–216 °C; FT-IR (ATR) cm^-1^, 3363, 3206 (H–N, amine), 2987, 2959 (H–C, CH_3_), 1,692 (C=O, amide), 1,569 (C=C, Ar), 1,345 (S=O, sulfonamide), 1,225 (C-N); ^**1**^ **H NMR** (400 MHz, DMSO-*d*_*6*_) *δ* 8.06–7.96 (m, 4H, Ar-H), 7.94 (t, *J* = 1.9 Hz, 1H, Ar-H), 7.87 (dt, *J* = 7.8, 1.3 Hz, 1H, Ar-H), 7.75 (ddd, *J* = 8.1, 2.2, 1.1 Hz, 1H, Ar-H), 7.60 (t, *J* = 7.9 Hz, 1H, Ar-H), 7.54 (s, 2H, CH_3_), 2.98 (s, 3H, CH_3_), 2.44 (s, 3H, CH_3_); ^**13**^ **C NMR** (101 MHz, DMSO-*d*_*6*_) *δ* 166.8, 154.0, 147.0, 145.4, 144.3, 137.1, 134.4, 132.5, 132.4, 130.4, 129.9, 129.4, 127.0, 122.3, 14.8, 11.9; ESI-MS calc. for [C_18_H_16_ClN_5_O_3_S + H]^+^, m/z 417.0662; found, m/z 418.2 [M + H]^+^; RT = 2.974; purity: 95.16%.

#### 2.1.5 (*E*)-4-((1-(4-Methoxybenzoyl)-3,5-dimethyl-1*H*-pyrazol-4-yl)diazenyl)benzenesulfonamide (5c)

Yellow solid; yield: 80%; M.P.: 202 °C–205 °C FT-IR (ATR) cm^-1^, 3271, 3059 (H–N, amine), 2994, 2959 (H–C, CH_3_), 1,695 (C=O, amide), 1,570 (C=C, Ar), 1,339 (S=O, sulfonamide), 1,225 (C-N); ^**1**^ **H NMR** (400 MHz, DMSO-*d*_*6*_) *δ* 8.05–7.95 (m, 6H, Ar-H), 7.53 (s, 2H, NH_2_), 7.16–7.05 (m, 2H, Ar-H), 3.89 (s, 3H, OCH_3_), 2.95 (s, 3H, CH_3_), 2.46 (s, 3H, CH_3_); ^**13**^ **C NMR** (101 MHz, DMSO-*d*_*6*_) *δ* 166.9, 163.2, 163.1, 154.1, 146.7, 145.2, 143.4, 136.9, 133.9, 126.9, 123.8, 122.3, 113.5, 55.6, 14.8, 11.7; ESI-MS calc. for [C_19_H_19_N_5_O_4_S + H]^+^, m/z 414.1158; found, m/z 414.3 [M + H]^+^; RT = 2.705; purity: 94.98%.

#### 2.1.6 (*E*)-4-((1-(3,4-Dimethoxybenzoyl)-3,5-dimethyl-1*H*-pyrazol-4-yl)diazenyl)benzenesulfonamide (5d)

Yellow solid; yield: 81%; M.P.: 226 °C –228 °C; FT-IR (ATR) cm^-1^, 3289, 3232 (H–N, amine), 2959, 2914 (H–C, CH_3_), 1,673 (C=O, amide), 1,592 (C=C, Ar), 1,345 (S=O, sulfonamide), 1,226 (C-N); ^**1**^ **H NMR** (400 MHz, DMSO-*d*_*6*_) *δ* 8.05–7.95 (m, 4H, Ar-H), 7.67 (dd, *J* = 8.5, 2.1 Hz, 1H, Ar-H), 7.57 (d, *J* = 2.1 Hz, 1H, Ar-H), 7.53 (s, 2H, NH_2_), 7.13 (d, *J* = 8.6 Hz, 1H, Ar-H), 3.89 (s, 3H, OCH_3_), 3.82 (s, 3H, OCH_3_), 2.93 (s, 3H, CH_3_), 2.46 (s, 3H, CH_3_); ^**13**^ **C NMR** (101 MHz, DMSO-*d*_*6*_) *δ* 166.9, 154.1, 153.2, 147.9, 146.7, 145.2, 143.4, 136.8, 126.9, 126.9, 126.4, 123.6, 122.3, 114.2, 110.5, 55.8, 55.6, 14.7, 11.7; ESI-MS calc. for [C_20_H_21_N_5_O_5_S + H]^+^, m/z 444.1263; found, m/z 444.2 [M + H]^+^; RT = 2.526; purity: 95.53%.

#### 2.1.7 (*E*)-4-((3,5-Dimethyl-1-(4-nitrobenzoyl)-1*H*-pyrazol-4-yl)diazenyl)benzenesulfonamide (5e)

Yellow solid; yield: 71%; M.P.: 231 °C–233 °C; FT-IR (ATR) cm^-1^, 3249, 3217 (H–N, amine), 2993, 2958 (H–C, CH_3_), 1,658 (C=O, amide), 1,585 (C=C, Ar), 1,358 (S=O, sulfonamide), 1,222 (C-N); ^**1**^ **H NMR** (400 MHz, DMSO-*d*_*6*_) *δ* 8.43–8.35 (m, 2H, Ar-H), 8.17–8.09 (m, 2H, Ar-H), 8.02 (s, 4H, Ar-H), 7.54 (s, 2H, NH_2_), 3.03 (s, 3H, CH_3_), 2.44 (s, 3H, CH_3_); ^**13**^ **C NMR** (101 MHz, DMSO-*d*_*6*_) *δ* 167.0, 154.0, 149.3, 147.1, 145.5, 144.7, 138.4, 137.2, 131.9, 131.7, 127.0, 122.9, 122.9, 122.4, 120.4, 14.8, 12.0; ESI-MS calc. for [C_18_H_16_N_6_O_5_S + H]^+^, m/z 429.0903; found, m/z 444.2 [M + H]^+^; RT = 2.690; purity: 96.53%.

#### 2.1.8 (*E*)-4-((3,5-Dimethyl-1-(3-nitrobenzoyl)-1*H*-pyrazol-4-yl)diazenyl)benzenesulfonamide (5f)

Yellow solid; yield: 76%; M.P.: 227 °C –229 °C; FT-IR (ATR) cm^-1^, 3201, 3091 (H–N, amine), 2983, 2959 (H–C, CH_3_), 1,696 (C=O, amide), 1,575 (C=C, Ar), 1,333 (S=O, sulfonamide), 1,289 (C-N); ^**1**^ **H NMR** (400 MHz, DMSO-*d*_*6*_) *δ* 8.72 (t, *J* = 2.0 Hz, 1H, Ar-H), 8.52 (ddd, *J* = 8.3, 2.5, 1.1 Hz, 1H, Ar-H), 8.35 (dt, *J* = 7.8, 1.3 Hz, 1H, Ar-H), 8.02 (s, 4H, Ar-H), 7.88 (t, *J* = 8.0 Hz, 1H, Ar-H), 7.54 (s, 2H, NH_2_), 3.03 (s, 3H, CH_3_), 2.45 (s, 3H, CH_3_); ^**13**^ **C NMR** (101 MHz, DMSO-*d*_*6*_) *δ* 166.2, 154.0, 147.2, 147.0, 145.5, 144.6, 137.2, 136.9, 134.0, 129.7, 127.0, 126.9, 125.5, 122.4, 120.4, 14.8, 12.0; ESI-MS calc. for [C_18_H_16_N_6_O_5_S + H]^+^, m/z 429.0903; found, m/z 429.2 [M + H]^+^; RT = 2.675; purity: 92.50%.

#### 2.1.9 (*E*)-4-((3,5-Dimethyl-1-(2-methylbenzoyl)-1*H*-pyrazol-4-yl)diazenyl)benzenesulfonamide (5g)

Yellow solid; yield: 79%; M.P.: 222 °C –224 °C; FT-IR (ATR) cm^-1^, 3305, 3214 (H–N, amine), 2959, 2914 (H–C, CH_3_), 1,680 (C=O, amide), 1,573 (C=C, Ar), 1,337 (S=O, sulfonamide), 1,294 (C-N); ^**1**^ **H NMR** (400 MHz, DMSO-*d*_*6*_) *δ* 8.06–7.96 (m, 4H, Ar-H), 7.56–7.44 (m, 4H, NH_2,_ Ar-H), 7.39–7.29 (m, 2H, Ar-H), 3.03 (s, 3H, CH_3_), 2.38 (s, 3H, CH_3_), 2.28 (s, 3H, CH_3_); ^**13**^ **C NMR** (101 MHz, DMSO-*d*_*6*_) *δ* 169.8, 154.0, 146.4, 145.4, 144.5, 137.2, 136.1, 133.7, 130.8, 130.3, 128.8, 127.0, 125.2, 122.3, 120.3, 19.2, 14.78, 11.97; ESI-MS calc. for [C_19_H_19_N_5_O_3_S + H]^+^, m/z 398.1209; found, m/z 398.2 [M + H]^+^; RT = 2.750; purity: 96.15%.

#### 2.1.10 (*E*)-4-((3,5-Dimethyl-1-(4-methylbenzoyl)-1*H*-pyrazol-4-yl)diazenyl)benzenesulfonamide (5h)

Yellow solid; yield: 81%; M.P.: 218 °C –220 °C; FT-IR (ATR) cm^-1^, 3258, 3110 (H–N, amine), 2993, 2958 (H–C, CH_3_), 1,690 (C=O, amide), 1,568 (C=C, Ar), 1,331 (S=O, sulfonamide), 1,289 (C-N); ^**1**^ **H NMR** (400 MHz, DMSO-*d*_*6*_) *δ* 8.05–7.96 (m, 5H, Ar-H), 7.85 (d, *J* = 8.2 Hz, 2H, Ar-H), 7.53 (s, 2H, NH_2_), 7.38 (d, *J* = 8.0 Hz, 2H, Ar-H), 2.96 (s, 3H, CH_3_), 2.43 (2s, 6H, 2 × CH_3_); ^**13**^ **C NMR** (101 MHz, DMSO-*d*_*6*_) *δ* 167.8, 154.0, 146.8, 145.3, 143.7, 143.6, 137.0, 131.3, 131.1, 129.3, 128.5, 128.5, 127.0, 122.3, 21.2, 14.8, 11.8; ESI-MS calc. for [C_19_H_19_N_5_O_3_S + H]^+^, m/z 398.1209; found, m/z 398.2 [M + H]^+^; RT = 3.400; purity: 92.43%.

#### 2.1.11 (*E*)-4-((1-(4-Bromobenzoyl)-3,5-dimethyl-1*H*-pyrazol-4-yl)diazenyl)benzenesulfonamide (5i)

Yellow solid; yield: 85%; M.P.: 235 °C –237 °C ; FT-IR (ATR) cm^-1^, 3369, 3207 (H–N, amine), 2984, 2958 (H–C, CH_3_), 1,693 (C=O, amide), 1,568 (C=C, Ar), 1,345 (S=O, sulfonamide), 1,289 (C-N); ^**1**^ **H NMR** (400 MHz, DMSO-*d*_*6*_) *δ* 8.05–7.96 (m, 4H, Ar-H), 7.91–7.83 (m, 2H, Ar-H), 7.83–7.76 (m, 2H, Ar-H), 7.54 (s, 2H, NH_2_), 2.98 (s, 3H, CH_3_), 2.43 (s, 3H, CH_3_); ^**13**^ **C NMR** (101 MHz, DMSO-*d*_*6*_) *δ* 167.3, 154.0, 147.0, 145.4, 144.1, 137.1, 132.9, 131.4, 131.0, 127.0, 126.9, 122.3, 14.8, 11.9; ESI-MS calc. for [C_18_H_16_BrN_5_O_3_ + H]^+^, m/z 462.0157/464.0157 found, m/z 462.1/464.1 [M + H]^+^; RT = 3.023; purity: 95.73%.

#### 2.1.12 (*E*)-4-((1-(3-Methoxybenzoyl)-3,5-dimethyl-1*H*-pyrazol-4-yl)diazenyl)benzenesulfonamide (5j)

Yellow solid; yield: 78%; M.P.: 198 °C –201 °C; FT-IR (ATR) cm^-1^, 3269, 3015 (H–N, amine), 2993, 2958 (H–C, CH_3_), 1731 (C=O, amide), 1,568 (C=C, Ar), 1,370 (S=O, sulfonamide), 1,212 (C-N); ^**1**^ **H NMR** (400 MHz, DMSO-*d*_*6*_) *δ* 8.06–7.95 (m, 4H, Ar-H), 7.74–7.67 (m, 2H, Ar-H), 7.54 (s, 2H, NH_2_), 7.51–7.43 (m, 2H, Ar-H), 2.97 (s, 3H, OCH_3_), 2.43 (s, 3H, CH_3_), 2.40 (s, 3H, CH_3_); ^**13**^ **C NMR** (101 MHz, DMSO-*d*_*6*_) *δ* 168.2, 154.0, 146.8, 145.3, 143.8, 137.4, 137.0, 133.5, 132.2, 131.1, 128.2, 127.8, 126.9, 122.3, 20.8, 14.8, 11.8; ESI-MS calc. for [C_19_H_19_N_5_O_4_S + H]^+^, m/z 414.1158; found, m/z 414.11 [M + H]^+^; RT = 2.862; purity: 94.94%.

#### 2.1.13 (*E*)-4-((3,5-Dimethyl-1*H*-pyrazol-4-yl) diazenyl)benzenesulfonamide (5k)

Yellow solid; yield: 81%; M.P.: 188 °C –190 °C; ^**1**^ **H NMR** (400 MHz, DMSO-*d*_*6*_) *δ* 12.98 (s, 1H, NH), 7.95 (d, *J* = 8.6 Hz, 2H, Ar-H), 7.86 (d, *J* = 8.6 Hz, 2H, Ar-H), 7.46 (s, 2H, NH_2_), 2.48 (s, 6H, 2 × CH_3_).

### 2.2 *In vitro* biological assay

An already established method was employed to perform *α*- and *β*-glucosidase inhibitory assays in 96-well plates (Kazmi et al., 2017). In this assay, phosphate buffer (0.07 M pH 6.8) was used to prepare 2.5 U/mL of *α*-glucosidase, 2.0 U/mL of *β*-glucosidase, and substrate 4-nitrophenyl-*β*-d-glucopyranoside (*p*-NPG) (10 mM) solutions. In addition, 1 mM solution of each test compound was prepared in 10% DMSO to achieve an end concentration of 100 *µ*M in each well. Initially, 10 *µ*L of the respective enzyme was incubated with 10 *µ*L of test compounds for 5 min at 37 °C. Subsequently, 10 *µ*L of *p*-NPG was added in each well followed by an incubation period of 30 min at 37 °C before adding 80 *µ*L of Na_2_CO_3_ (200 mM) as a stop solution to each well. The absorbance was measured at 405 nm, and the percentage inhibition for each of the test compounds was determined by the following equation.

Percentage inhibition = 100–[Slope of test compound/Slope of enzyme control] × 100.

Acarbose was used as a standard drug for evaluating the efficacy of test compounds against *α*- and *β*-glucosidase. The inhibitory concentration 50 (IC_50_) for each of the test compound was determined by GraphPad prism version 10.0.

### 2.3 *In silico* investigation

#### 2.3.1 Protein model development and validation

Modeller 10.3 homology modeling software was used to construct the 3D structure of *α*-glucosidase (http://salilab.org/modeller/) taking the crystalline structure of *Saccharomyces cerevisiae* isomaltase (PDB id: 3AJ7) as a reference. The model was prepared by providing the amino acid sequence of *α*-glucosidase in FASTA format obtained from UniProt (access code P53341) (Kazmi et al., 2017). The quality of the model and its stereochemical features were then evaluated by the Ramachandran plot (LASKOWSKI et al., 2013) obtained from PROCHECK version 3.5 (https://saves.mbi.ucla.edu) (Hasan et al., 2015). Additionally, Verify3D (Eisenberg et al., 1997), ERRAT (Hasan et al., 2015), and ProSA-web (https://prosa.services.came.sbg.ac.at/prosa.php) (Zaib et al., 2023) were also utilized to validate the predicted 3D structure of *α*-glucosidase.

#### 2.3.2 Molecular docking

**FIGURE 2:**
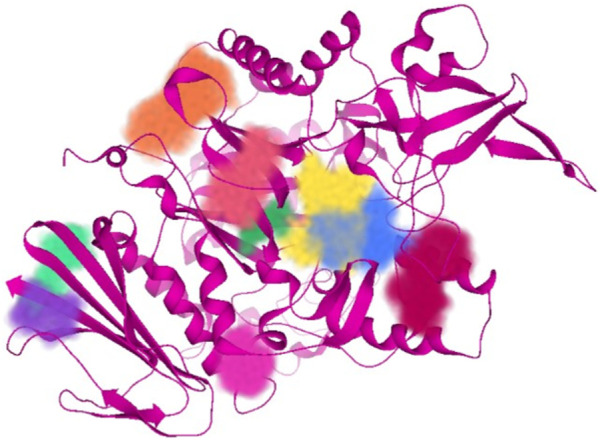
All unoccupied sites of *α*-glucosidase that can be probable druggable binding sites.

Subsequent to 3D structure prediction of the target protein (*α*-glucosidase), the binding affinities of the potent inhibitors were evaluated using the FlexX functionality of SeeSAR version 13.0 (www.biosolveit.de/SeeSAR) (BioSolveIT GmbH, 2023). The protein was uploaded in the protein mode followed by the selection of the binding site in the binding site mode. Alpha-glucosidase contains 09 unoccupied sites, as indicated by various colors in Figure 2, having varied amino acid residues, DoGSiteScore, volume, and surface area (Table 1). All these binding sites were evaluated individually to analyze the binding of potent inhibitors, and it was found that the second binding site (dark pink) was the optimum druggable binding site for pyrazole containing sulfonamide derivatives (Ibrar et al., 2016). Thence, the potent inhibitors were docked in the selected binding site *via* standard docking, and the best pose was selected based on the lowest binding energy and highest binding affinity (Dera et al., 2023).

**TABLE 1:** Illustration of properties of binding pockets of specific colors, as represented in Figure 2.

Pocket ID		Number of residues	DoGSiteScore	Number of donors	Number of acceptors	Hydrophobicity	Solvent accessible surface (Å^2^)	Total volume of the pocket (Å^3^)
	1	29	0.38	21	21	0.67	462.60	569.59
	2	17	0.36	10	11	0.74	260.28	304.99
	3	13	0.22	6	7	0.76	121.32	86.40
	4	20	0.20	12	10	0.66	254.16	354.24
	5	10	0.43	3	5	0.81	105.48	195.48
	6	11	0.27	5	4	0.76	123.12	159.41
	7	12	0.11	7	6	0.71	106.92	125.06
	8	26	0.10	16	21	0.62	308.52	402.84
	9	17	0.10	9	11	0.66	212.76	243.22

#### 2.3.3 Pharmacokinetic evaluation

The pharmacokinetic properties of the potent inhibitors were determined by SwissADME (swissadme.ch/index.php) which helps the determination of druggability, lipophilicity, skin permeability, gastrointestinal absorption, and the medicinal properties of the query compound. The results are interpreted in tabular form (Mahanthesh et al., 2020; Sravika et al., 2021).

#### 2.3.4 Intermolecular interaction visualization

Intermolecular interactions such as hydrogen bond and hydrophobic interactions formed between the potent inhibitors and *α*-glucosidase were visualized by BIOVIA Discovery Studio molecular visualizer 2021 (Baskaran et al., 2020).

#### 2.3.5 Hydrogen bond and Dehydration energy

The role of each atom of potent inhibitors in the binding affinity was determined by the Hydrogen bond and Dehydration energy (HYDE) calculated by SeeSAR version 13.0. The results are represented qualitatively by colors and quantitatively by energy scores (Zaib et al., 2023).

## 3 Results and discussion

### 3.1 Synthesis and characterization

**SCHEME 1:**
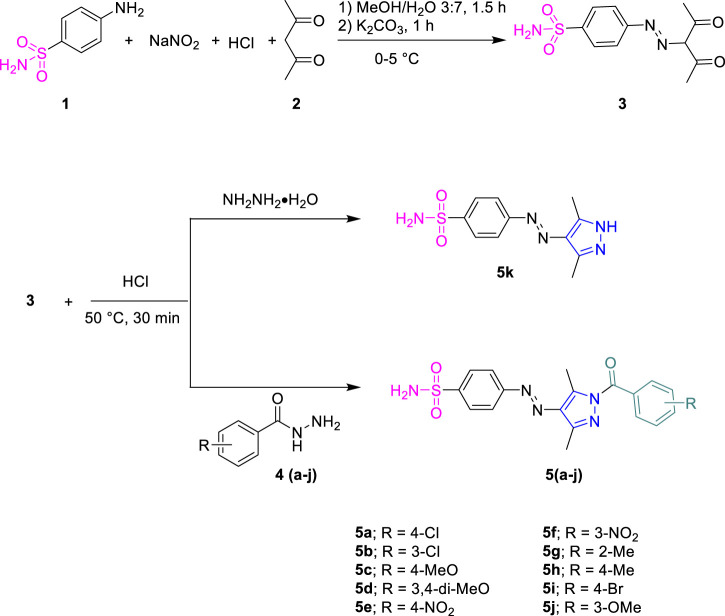
Synthetic pathway for acyl pyrazole sulfonamides (**5a-k**).

Sulfanilamide **1** was coupled with active methylene of acetylacetone **2** by the diazotization reaction to furnish intermediate **3**, which was reacted with different substituted hydrazides **4a-j** and hydrazine monohydrate in ethanol under acidic conditions (Scheme 1). The desired products were precipitated out during the reaction and were filtered and washed with ethanol.

The formation of sulfonamide-clubbed pyrazole derivatives **(5a-k)** was indicated by their FTIR spectral data, where characteristic absorptions were observed in the range of 3300–3000 cm^-1^ attributed to the N-H of the NH_2_ group, in addition to those of C-H stretching vibrations in the range of 2990–2870 cm^-1^. The peaks at 1,580 cm^-1^ and 1,370 cm^-1^ corresponded to the stretching frequency of the aromatic (C=C) and sulfonamide (S=O) moiety, respectively. Additionally, a strong absorption peak of 1700 cm^-1^ was attributed to carbonyl (C=O) of the amide linkage. In ^1^H NMR spectra, the N–H proton of the primary amine resonated at 7.53 ppm along with the additional aromatic protons in their respective regions. The appearance of two singlets for CH_3_ substituents at a pyrazole ring also confirmed the formation of synthesized derivatives. ^13^C NMR spectra also aided in the confirmation of compounds **5a-k**, where distinctive signals for carbonyl were observed around 167.3 ppm. The additional resonance for the carbon-related methyl alkyl part was also observed in the range of 11.94–14.86 ppm. The other aromatic signals appeared in the corresponding region with appropriate chemical shift values.

### 3.2 *In vitro* biological activity

Initially, the synthesized derivatives were investigated at a concentration of 1 mM, and percentage inhibition was employed as an indicator of activity against both enzymes. It was observed that all compounds were more effective against *α*-glucosidase than *β*-glucosidase (Table 2). IC_50_ values were calculated for compounds having a percentage inhibition higher than 50%. The synthesized compounds showed varying trends for inhibition by altering the substitutions on the phenyl ring. Among these, compound **5a** displayed the highest activity, being 35 times more potent than the positive control, acarbose (IC_50_ = 35.1 ± 0.14 *µ*M). The overall decreasing inhibitory trends within the series were found as **5a**>**5b** > **5f** > **5c** > **5g** > **5e** > **5d**> **5h** > **5j** > **5i** > **5k** (Table 2).

**TABLE 2:** Inhibitory potential of pyrazole-clubbed sulfonamide derivatives (**5a-k**) against *α*-glucosidase and *β*-glucosidase.

Compound	Structures	*α*-glucosidase inhibition	*β*-glucosidase inhibition
		IC_50_ ± SEM (*µ*M)	%age inhibition
**5a**	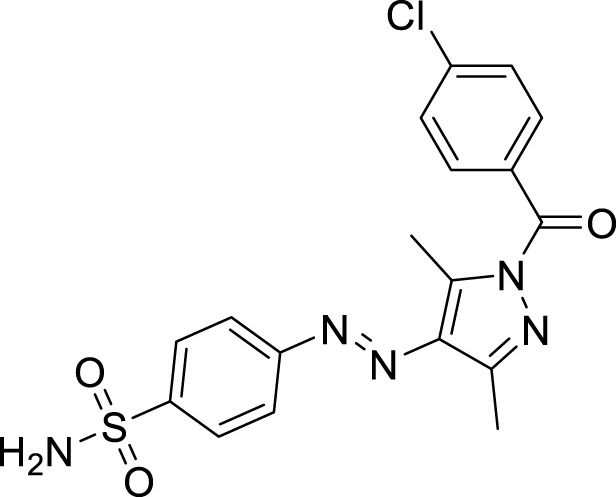	1.13 ± 0.06	34.3
**5b**	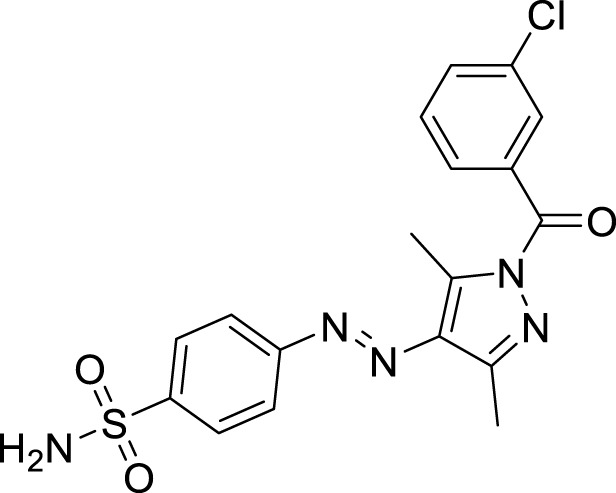	2.22 ± 0.11	32.1
**5c**	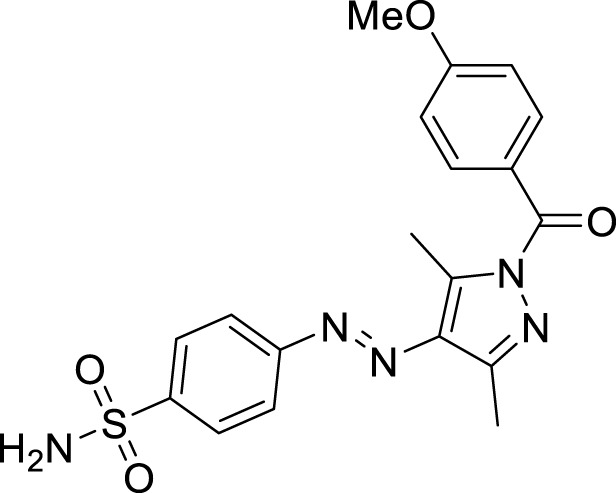	3.29 ± 0.09	12.9
**5d**	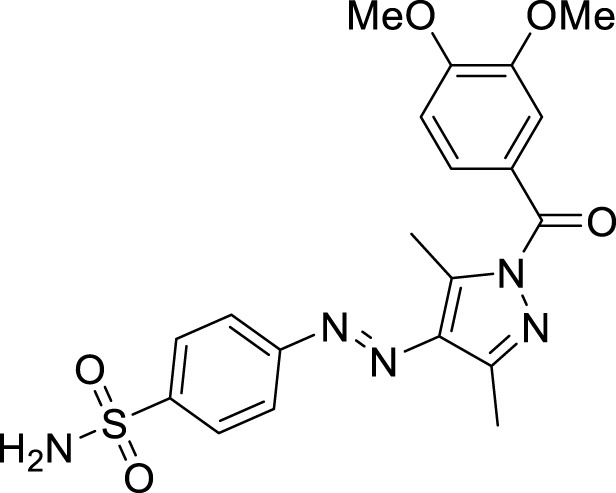	4.53 ± 0.12	18.5
**5e**	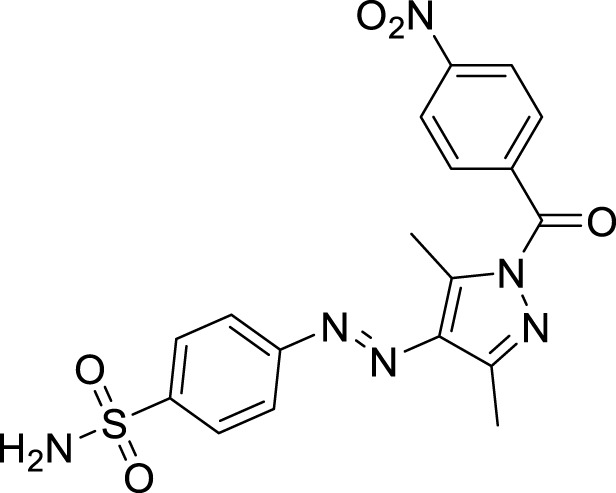	4.11 ± 0.19	11.7
**5f**	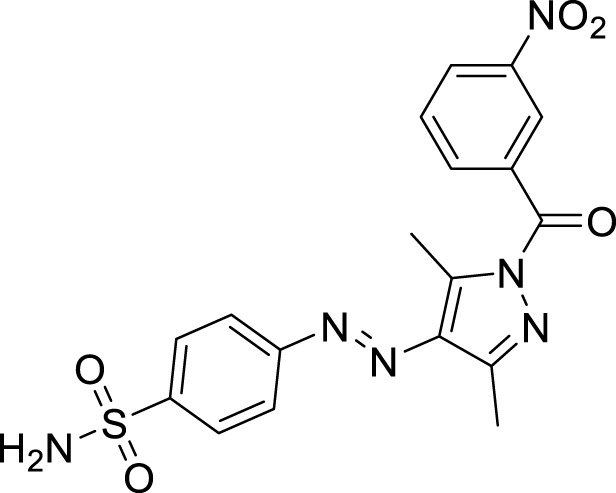	2.77 ± 0.24	27.4
**5g**	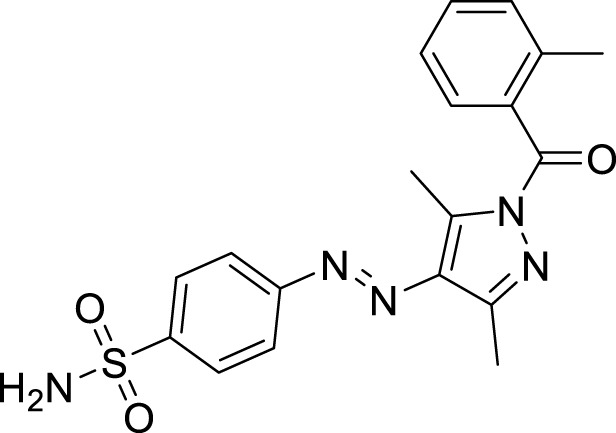	3.47 ± 0.43	21.0
**5h**	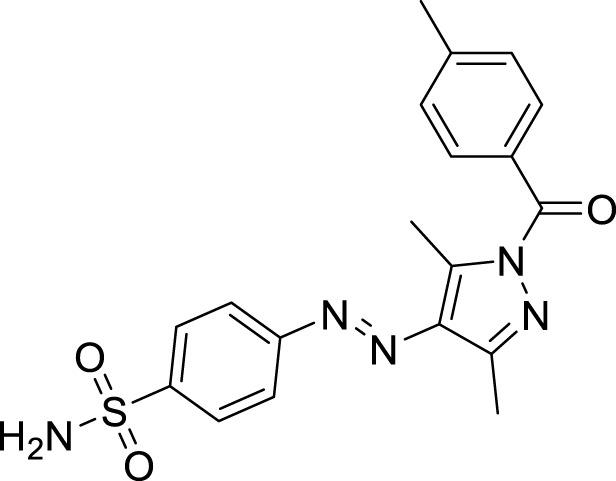	9.22 ± 0.45	14.6
**5i**	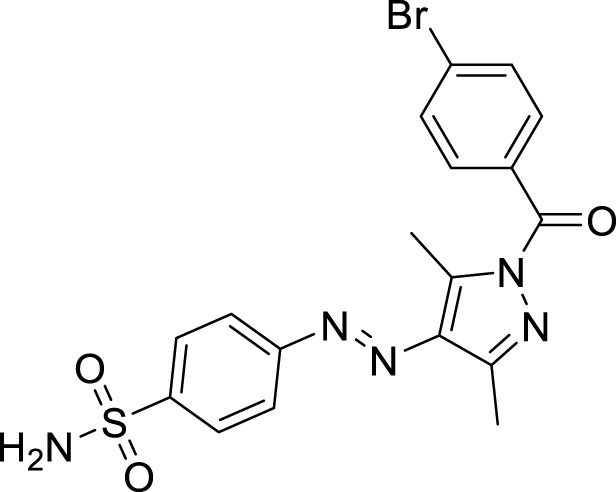	19.13 ± 1.07	11.2
**5j**	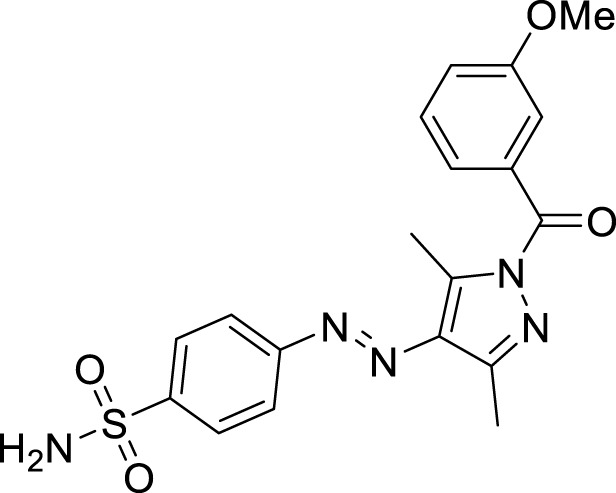	17.88 ± 1.16	7.55
**5k**	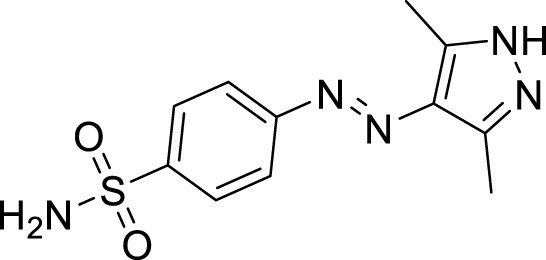	28.27 ± 1.45	25.1
**Acarbose**		35.1 ± 0.14	63.7

### 3.3 Structure–activity relationship studies

**FIGURE 3:**
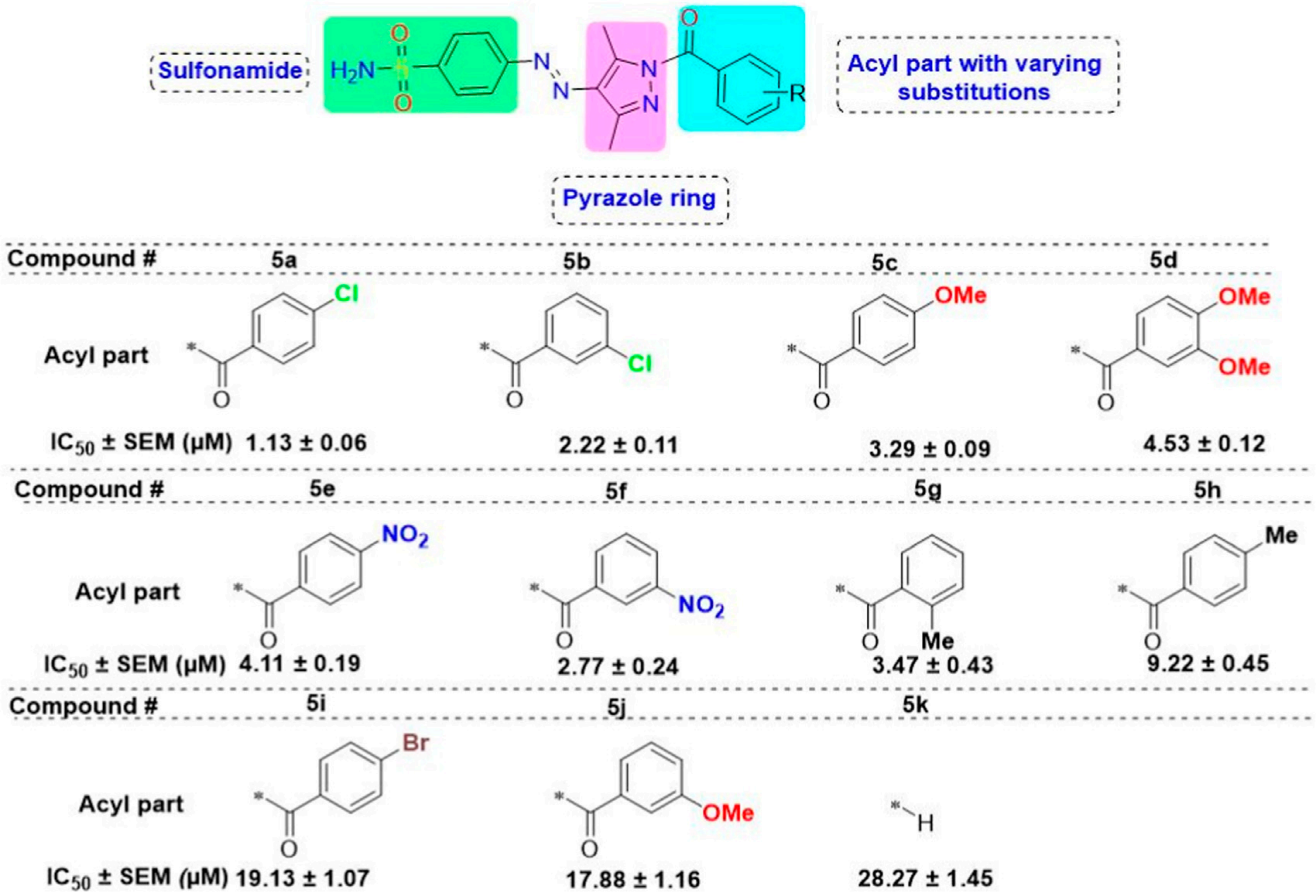
Target scaffold incorporating acyl pyrazole-clubbed sulfonamides **5(a–k)**; positive control: acarbose (IC_50_ = 35.1 ± 0.14 *µ*M).

The generic structure presented in Figure 3 comprises three components: 1) the terminal aryl sulfonamide moiety linked to the diazo unit; 2) central pyrazole heterocycle; 3) an acyl part attached to the pyrazole nucleus. The sulfonamide and pyrazole components remained unaltered, whereas a diverse variety of substituents was introduced on the acyl component. On evaluation of *in vitro* inhibitory data, various structure–activity relationships (SARs) revealed that the glucosidase inhibitory activity of pyrazole containing sulfonamide derivatives altered by varying the position and nature of the substituent on the acyl group (Figure 3). Among all the synthesized compounds, **5a** bearing a chlorine atom at the *para-*position of the phenyl ring was the most potent member of the developed library, with an IC_50_ value of 1.13 ± 0.06 *µ*M. This inhibitory efficacy of **5a** was 35-fold higher than the standard acarbose (IC_50_ = 35.1 ± 0.14 *µ*M). When switching the chlorine from the *para*- to *meta*-position (**5b**), a 50% reduction in activity was observed, although the inhibitory strength was still 17-fold higher than that of acarbose. The introduction of a bulky halogenated bromine atom instead of chlorine led to a drastic decrease in the inhibitory profile, as compound **5i** showed an IC_50_ value of 19.13 ± 1.07 *µ*M; however, the activity strength could be improved by introducing a strongly polarizable electron-withdrawing nitro substituent (**5e**). This compound displayed an IC_50_ value of 4.11 ± 0.19 *µ*M, an eight-fold stronger inhibition than the standard inhibitor. A further improvement in the inhibitory profile was noticed when an electron-withdrawing group (NO_2_) was replaced with an electron-donating group (OMe) in compound **5c**. Adding more electron-rich substituents such as the methoxy group in **5d** or removing the electron-rich character in **5h** led to a decrease in inhibitory efficacy. Both compounds exhibited IC_50_ values of 4.53 ± 0.12 and 9.22 ± 0.45 *µ*M, respectively. On the other hand, replacing the chloro substituent at a *meta*-position of **5b** with the nitro group produced a slight decrease in inhibition; however, methoxy-substituted derivative **5j** exhibited a sharp decline in glucosidase inhibition. The only *ortho*-substituted derivative, **5g**, showed a better inhibitory profile comparable to compound **5c**, with an IC_50_ value of 3.47 ± 0.43 *µ*M, whereas a drastic reduction in activity was observed when the acyl component was completely removed (compound **5k**; IC_50_ = 28.27 ± 1.45 *µ*M), making this derivative the least active member of the tested series. Overall, according to the behavior of these substituents in the active site of *α*-glucosidase, it was observed that the presence of an electron-withdrawing group (EWG) such as chlorine at *meta*- and *para*-positions increased the inhibitory potential. However, derivatives having an electron-donating group (EDG), such as methoxy at *meta*-position, showed a significant decrease in enzyme inhibitory activity. These observations also showed that the presence of an acyl component is necessary for the better induction of biological function.

On the contrary, none of the derivatives showed inhibitory efficacy against *β*-glucosidase enzyme. All the compounds showed inhibition in the range of 7.55%–34.3%.

### 3.4 *In silico* investigation

#### 3.4.1 Protein model development and validation

**FIGURE 4:**
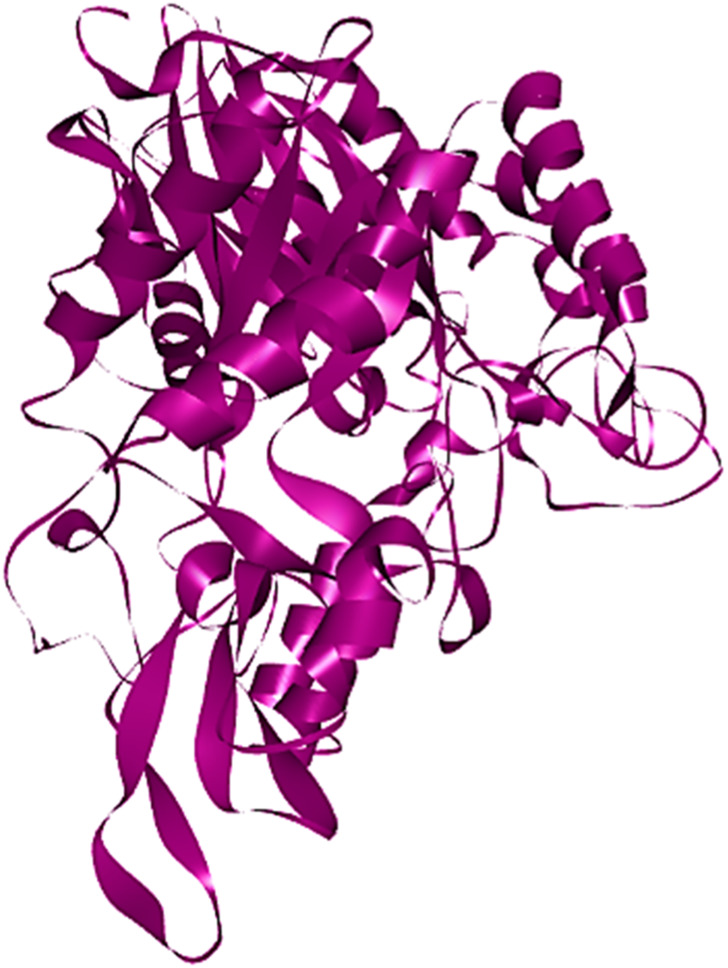
Visualization of the crystal structure of the best model of *α*-glucosidase predicted via Modeller.

The enzyme *α*-glucosidase sequence (583 amino acids) of *S. cerevisiae* was used for model development through Modeller version 10.3. It was observed that there was a good similar identity score with the protein having PDB ID of 3AJ7, so it was chosen as the template for model development. Five different models were developed, and the one with the best score and energy was taken for further evaluation. The crystallographic structure of the model is shown in Figure 4.

**FIGURE 5:**
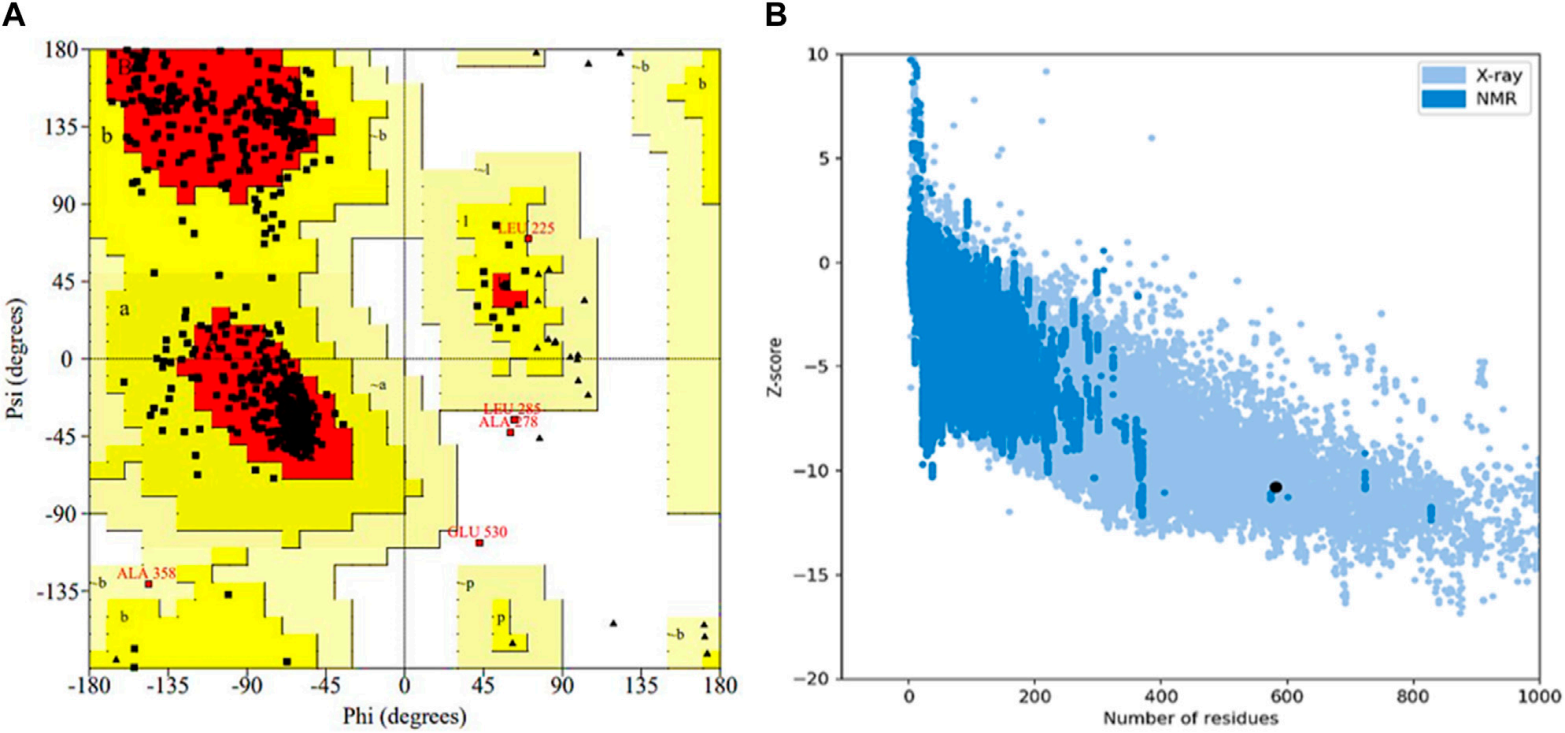
Validation of the 3D structure of α-glucosidase. **(A)** Ramachandran plot for the tertiary structure of *α*-glucosidase. A number of amino acids (83.9%) reside in the most favorable (red) region of the plot. However, a few lie in the acceptable (yellow), generously acceptable (pale yellow), and restricted region (white) of the graph. **(B)** Illustration of the model quality based on *z*-score. The higher the value of the *z*-score, the greater the model quality. The *z*-score for the predicted *α*-glucosidase model is −10.78.

The selected protein model was verified by Ramachandran plot analysis. It was predicted that 83.9% of amino acid residues (432 amino acids) would lie in the most favorable region (red color), while 15.1% pf amino acid residues (78 amino acids) would reside in the allowed region of the plot. However, only two amino acid residues (0.4%) were present in the generously allowed region (pale yellow), while three amino acid residues (0.6%) existed in the disallowed region (white color) (Figure 5A). Afterward, the *z*-score was determined by protein structure analysis (ProSA)-web which predicted the quality of the model (Figure 5B). The *z*-score of the final model was −10.78, which was similar to the *z*-score of the protein with similar amino acids (Lee et al., 2014).

**FIGURE 6:**
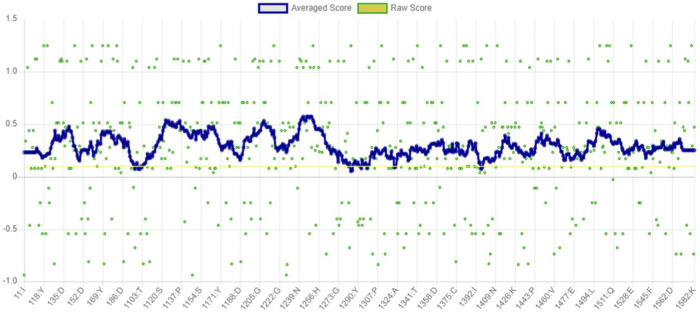
Graphical representation of averaged 3D-1D score of α-glucosidase obtained from VERIFY3D. The green dots in the graph indicate the amino acid residues of *α*-glucosidase, which are more aggregated in the region above 0.1.

The VERIFY3D evaluates the compatibility between the specific amino acid sequence and protein model. It determines the stability of the crystal structure of protein, identifies the most stable form of the folded protein assembly, and detects analogous protein sequences that exhibit a similar overall folding arrangement (Dym et al., 2006). According to the graph in Figure 6, nearly 97.94% of the residues have greater than or equal to 0.1 of mean 3D-1D score. Therefore, the predicted model of *α*-glucosidase passed the VERIFY3D validation.

**FIGURE 7:**
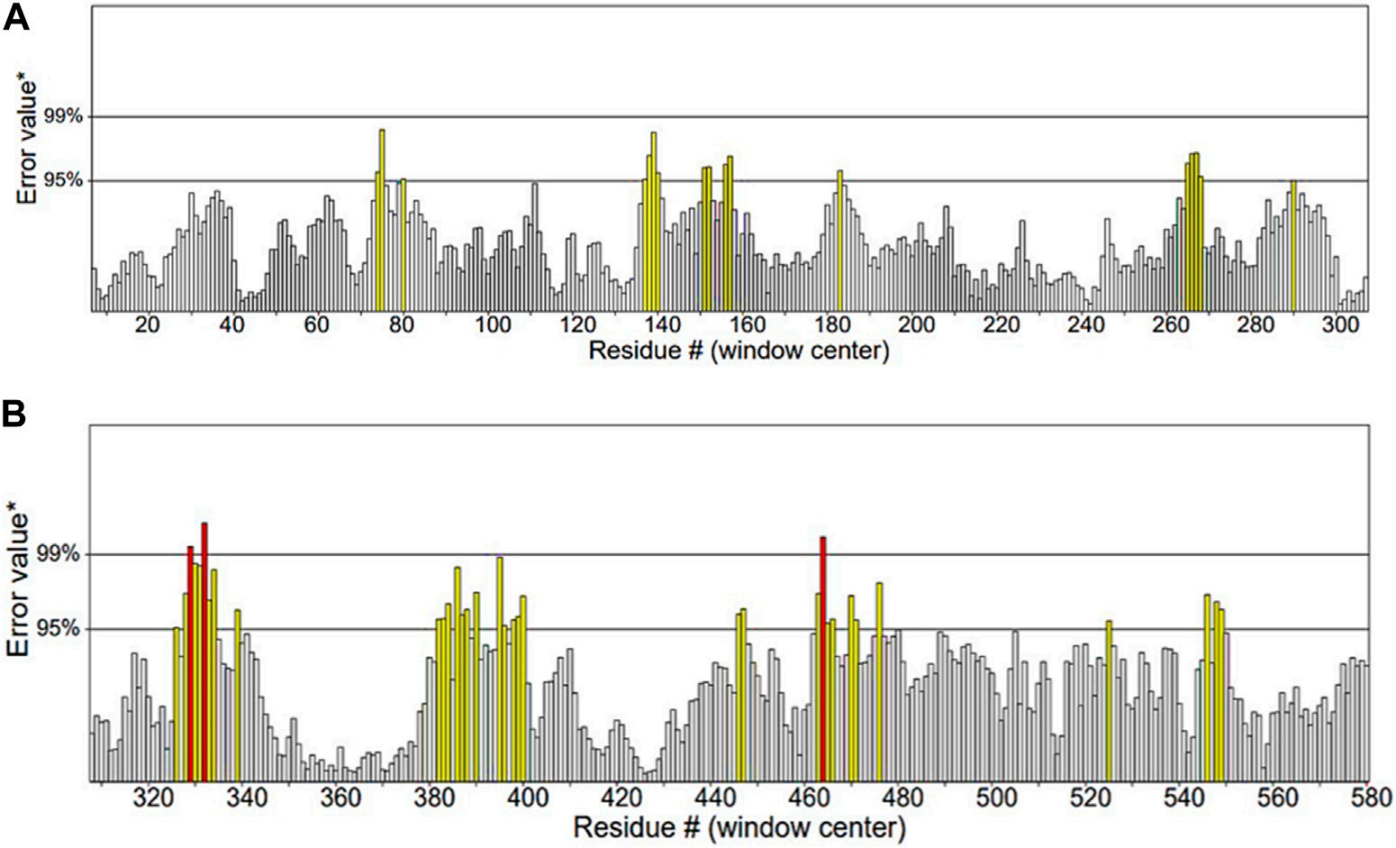
Quality factor plot of *α*-glucosidase obtained from ERRAT demonstrating that the overall peaks of error value for each of the graph are less than 95%. **(A)** Quality factor plot of 1 to 300 amino acid residues of *α*-glucosidase. **(B)** Quality factor plot of 300 to 580 amino acid residues of *α*-glucosidase.

ERRAT functions are based on the premise that various types of atoms within proteins will exhibit a non-random arrangement in relation to each other (Dym et al., 2006). It predicted that the overall quality factor of the *α*-glucosidase model was 91.115%. It also indicated that the resolution of the protein structure was between 2.5 and 3 Å (Figure 7).

#### 3.4.2 Molecular docking

**FIGURE 8:**
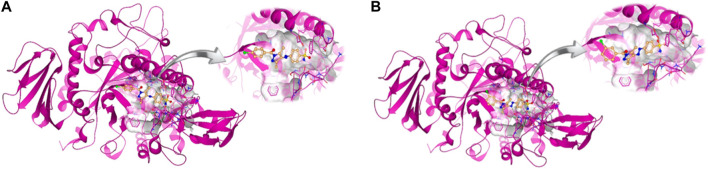
Docked complexes of **5a (A)** and **5b (B)** in the druggable active site of *α*-glucosidase.

**FIGURE 9:**
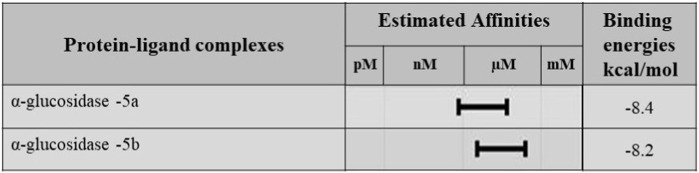
Binding affinities and binding energies of **5a** and **5b** in complex with *α*-glucosidase.

Molecular docking studies were performed for the *in silico* evaluation of protein–ligand binding *via* the FlexX feature of SeeSAR version 13.0 (www.biosolveit.de/SeeSAR) (BioSolveIT GmbH, 2023). The developed model was docked with two of the most potent inhibitors: compounds **5a** and **5b** (Figure 8). The calculated binding energies and affinities were congruent with the *in vitro* analysis. The binding affinity and energy of **5a** was in the nanomolar range and −8.4 kcal/mol, respectively. However, the binding energy and affinity of the docked complex of **5b** was slightly lower at −8.2 kcal/mol in the micromolar range (Figure 9).

#### 3.4.3 Pharmacokinetic analysis

The pharmacokinetic evaluation of **5a** and **5b** predicted that both isomers have the same physiochemical properties (Table 3). Their molecular weight is 417.87 g/mol with five rotatable bonds, seven hydrogen bond acceptors, one hydrogen bond donor, and 28 heavy atoms. The molar refractivity of both the inhibitors was 104.95 m^3^mol^−1,^ while their topological polar surface area (TPSA) was 128.15 Å^2^. They are moderately soluble, have low digestive tract absorption, and do not permeate the blood–brain barrier. In addition, they can exhibit minute hepatotoxicity by inhibiting two of the five cytochrome P450, such as CYP2C19 and CYP2C9. Both inhibitors do not permeate the skin as their log Kp is −6.34 cm/s and follow all druggable criteria.

**TABLE 3:** Pharamcokinetic analysis of potent inhibitors **5a** and **5b**.

Attributes	5a	5b
Formula	C_18_H_16_ClN_5_O_3_S	C_18_H_16_ClN_5_O_3_S
Molecular weight (g/mol)	417.87	417.87
Number of heavy atoms	28	28
Number of aromatic heavy atoms	17	17
Fraction C (sp^3^)	0.11	0.11
Number of rotatable bonds	5	5
Number of H-bond acceptors	7	7
Number of H-bond donors	1	1
Molar refractivity (m^3^mol^−1^)	104.95	104.95
TPSA (Å^2^)	128.15	128.15
Consensus log *P*_o/w_	3.29	3.28
Solubility	Moderately soluble	Moderately soluble
Digestive tract absorption	Low	Low
Blood–brain barrier	No	No
P-gp substrate	No	No
CYP1A2 inhibitor	No	No
CYP2C19 inhibitor	Yes	Yes
CYP2C9 inhibitor	Yes	Yes
CYP2D6 inhibitor	No	No
CYP3A4 inhibitor	No	No
Log Kp (cm/s)	−6.34	−6.34
Lipinski	Yes	Yes
Ghose	Yes	Yes
Veber	Yes	Yes
Egan	Yes	Yes
Muegge	Yes	Yes
Synthetic accessibility	3.11	3.15

#### 3.4.4 Intermolecular interaction visualization

**FIGURE 10:**
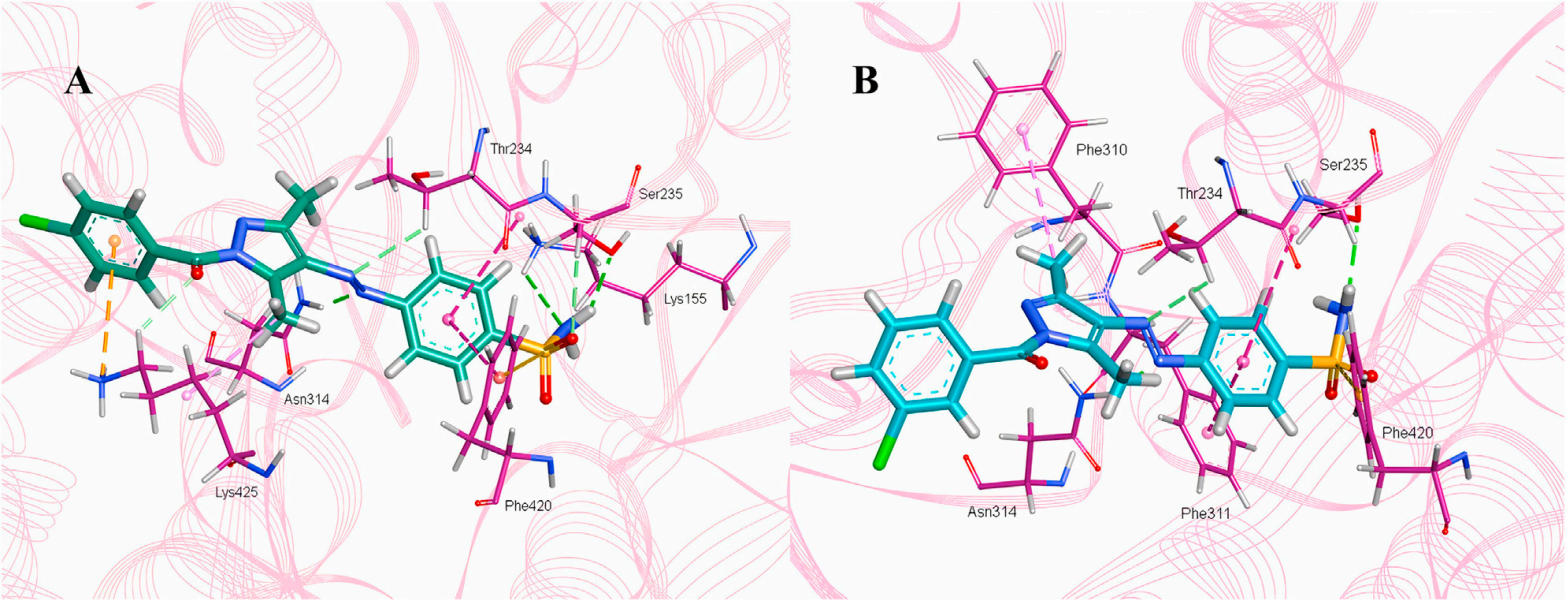
Intermolecular interactions between potent inhibitors and most druggable binding site of *α*-glucosidase. **(A)** Atoms of **5a** (green) interact with the binding pocket residues (dark pink) of the target protein. **(B)** The atoms of **5b** (light blue) interact with the active site residues (dark pink) of the target protein. In both figures, dotted lines indicate intermolecular interactions. Green represents the conventional hydrogen bond; light blue indicates the carbon hydrogen bond; light pink elucidates the alkyl and π–alkyl bond; π–π T-shaped and amide–π stacked bond are represented in dark pink. Orange color predicts π–cation and π–sulfur bonds.

The intermolecular interactions were predicted *via* Discovery Studio 2021 molecular visualization tool. Compounds **5a** and **5b** exhibit favorable interactions with the druggable active site of *α*-glucosidase (Table 4). Compound **5a** specifically develops hydrogen bonds, alkyl bonds, *π*–cation, *π*–sulfur, *π*–*π* T-shaped bond, and an amide–*π* stacked bond with the active site residues. Lys155 and Asn314 form conventional hydrogen bonds with N10 and N11 of **5a**, respectively. Another conventional hydrogen bond with H33 of **5a** is attributed to the presence of Ser235 in the active site, which also develops a carbon hydrogen bond with O8. Additionally, Thr234 and Lys425 also form carbon hydrogen bonds with N12 and O22 of the most potent ligand. As well as carbon hydrogen bonds, Thr234 also develops an amide-*π* stacked bond with the aromatic ring of the sulfonamide group of **5a**. Similarly, the same aromatic group is also involved in developing a *π*–*π* T-shaped bond with the Phe420 of the binding site. Along with the *π*–*π* T-shaped bond, Phe420 also contributes to the formation of a *π*–sulfur bond with the S7 of **5a**. Finally, Lys425 exhibits *π*–sulfur and alkyl bonds with the aromatic ring of the acyl group and C18 of the ligand (Figure 10A).

**TABLE 4:** Binding interactions of most potent inhibitors.

Most potent compounds	Binding interactions			
	Ligand atoms	Receptor residues	Interaction types	Distance (Å)
**5a**	N10	Lys155	H-bond	3.09
	N12	Thr234	H-bond	2.89
	O8	Ser235	H-bond	2.80
	H33	Ser235	H-bond	2.13
	N11	Asn314	H-bond	1.92
	O22	Lys425	H-bond	2.49
	C18	Lys425	Alkyl bond	4.35
	Aromatic ring	Lys425	*π*-cation	4.46
	S7	Phe420	*π*-sulfur	5.25
	Aromatic ring	Phe420	*π*-*π* T-shaped	5.13
	Aromatic ring	Thr234	Amide-*π* stacked bond	4.52
**5b**	N12	Thr234	H-bond	2.82
	H34	Ser235	H-bond	2.14
	N11	Asn314	H-bond	1.92
	C19	Phe310	*π*-alkyl	4.91
	S7	Phe420	*π*-sulfur	5.83
	Aromatic ring	Phe311	*π*-*π* T-shaped	4.23
	Aromatic ring	Thr234	Amide-*π* stacked bond	4.97

Compound **5b** develops conventional hydrogen bonds, carbon hydrogen bond, *π*–alkyl, *π*–sulfur, and*π*–*π* T-shaped and amide–*π* stacked bonds. The intermolecular interactions are simpler than **5a**. Two conventional hydrogen bonds are formed by Ser235 and another by Asn314 with H34 and N11 of **5b**. On the other hand, Thr234 forms a carbon hydrogen bond and an amide–*π* stacked bond with N12 and an aromatic ring of the sulfonamide group of **5b**. Phe310, Phe311, and Phe420 of the binding site exhibit *π*–alkyl, and *π*–*π* T-shaped and *π*–sulfur interaction with C19, aromatic ring, S7 of **5b**, respectively (Figure 10B).

#### 3.4.5 HYdrogen bond and DEhydration energy

**FIGURE 11:**
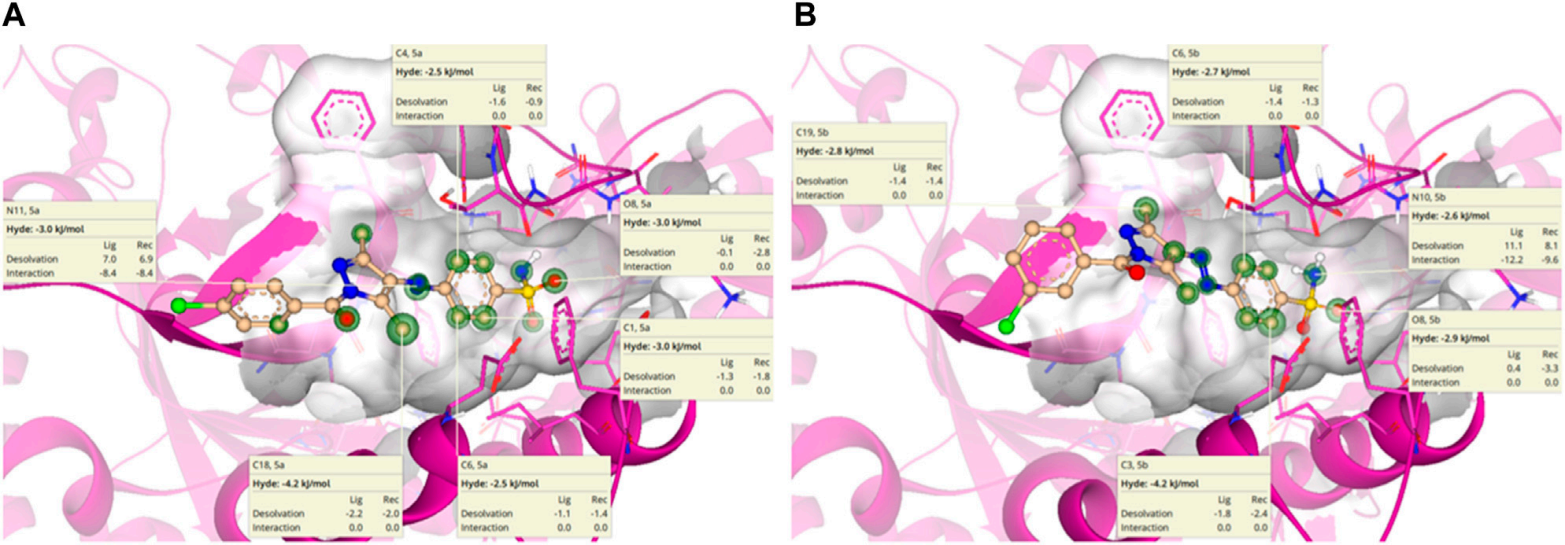
HYDE estimation of **5a** **(A)** and **5b** **(B)** in which green circles represent good HYDE scores. The greater the width of the green circle, the more the contribution of the atom in the interaction with *α*-glucosidase.

HYdrogen bond and DEhydration energy (HYDE) for **5a** and **5b** are predicted by SeeSAR version 13.0 (Figure 11). According to Figure 11A, atoms such as C1, C4, C6, O8, N14, and C18 of **5a** have a HYDE score less than −2.5 kJ/mol; among these, C18 has the lowest HYDE. The lower the value of HYDE, the greater the involvement of the atom in the binding affinity.

In the case of **5b** (Figure 11B), the lowest HYDE is exhibited by C3, which was −4.2 kJ/mol. However, other atoms such as C6, O8, N10, and C19 also have contributions to the binding affinity of **5b** with *α*-glucosidase.

## 4 Conclusion

This research demonstrated an efficient method for producing structurally diverse pyrazole–sulfonamide conjugates **(5a–k)** by a diazotization reaction of sulfonamide amine with acetylacetone followed by a cyclization reaction with a diverse array of substituted hydrazides and hydrazine monohydrate in ethanol. This approach was beneficial since it led to the formation of a pyrazole ring, an important medicinal heterocycle, while simultaneously circumventing extensive purification methods and minimizing chemical waste. The structural variability of the synthesized analogs was assured by using a diverse range of substituents on the aromatic hydrazides. The glucosidase inhibition potential was evaluated for synthesized conjugates (**5a–k**) where most of the compounds were prominently active and selective against the *α*-glucosidase enzyme. Among them, **5a** containing chlorine at a *para*-position was identified as the most potent and selective inhibitor, with an IC_50_ value of 1.13 ± 0.06 *µ*M (acarbose: IC_50_ = 35.1 ± 0.14 *µ*M). Consequently, based on the activity findings and docking studies, these compounds could potentially be developed as a novel series of structurally varied, potent, efficacious, and highly selective *α*-glucosidase inhibitors.

## Data Availability

The original contributions presented in the study are included in the article/Supplementary Material; further inquiries can be directed to the corresponding authors.

## References

[B1] Abdelgawad M. A. Labib M. B. Ali W. A. M. Kamel G. Azouz A. A. El-Nahass E. S. (2018). Design, synthesis, analgesic, anti-inflammatory activity of novel pyrazolones possessing aminosulfonyl pharmacophore as inhibitors of COX-2/5-LOX enzymes: histopathological and docking studies. Bioorg Chem. 78, 103–114. 10.1016/j.bioorg.2018.03.011 29550530

[B2] Abdul-Gafoor H. S. (2016). Synthesis and *in vitro* antitumor activity of some new fused pyrazole ring system containing sulfonamide, sulfonylurea and thiourea pharmacophores as anti-cancer agents. Dr. Diss. King Abdulaziz University–Jeddah.

[B3] Akhtar J. Khan A. A. Ali Z. Haider R. Shahar Yar M. (2017). Structure-activity relationship (SAR) study and design strategies of nitrogen-containing heterocyclic moieties for their anticancer activities. Eur. J. Med. Chem. 125, 143–189. 10.1016/j.ejmech.2016.09.023 27662031

[B4] Ansari K. F. Lal C. (2009). Synthesis and biological activity of some heterocyclic compounds containing benzimidazole and beta-lactam moiety. J. Chem. Sci. 121, 1017–1025. 10.1007/s12039-009-0114-8

[B5] Azimi F. Azizian H. Najafi M. Hassanzadeh F. Sadeghi-Aliabadi H. Ghasemi J. B. (2021). Design and synthesis of novel quinazolinone-pyrazole derivatives as potential α-glucosidase inhibitors: structure-activity relationship, molecular modeling and kinetic study. Bioorg. Chem. 114, 105127. 10.1016/j.bioorg.2021.105127 34246971

[B6] Badampudi S. K. Aluru R. G. P. Papammagari R. R. Rao R. L. R. K. (2014). Synthesis, characterization and pharmacological evaluation of certain sulfonamide containing heterocyclic motifs. Pol. Ann. Med. 21 (2), 75–81. 10.1016/j.poamed.2014.07.006

[B7] Baskaran K. P. Arumugam A. Kandasamy R. Alagarsamy S. (2020). *In silico* method for prediction of maximum binding affinity and ligand-protein interaction studies on Alzheimer’s disease. Int. J. Res. Granthaalayah. 8, 362–370. 10.29121/granthaalayah.v8.i11.2020.2472

[B8] Bayrak C. (2022). Synthesis and aldose reductase inhibition effects of celecoxib derivatives containing pyrazole linked-sulfonamide moiety. Bioorg. Chem. 128, 106086. 10.1016/j.bioorg.2022.106086 35973306

[B9] Bellary S. Kyrou I. Brown J. E. Bailey C. J. (2021). Type 2 diabetes mellitus in older adults: clinical considerations and management. Nat. Rev. Endocrinol. 17 (9), 534–548. 10.1038/s41574-021-00512-2 34172940

[B10] Chalkha M. Nakkabi A. Hadda T. B. Berredjem M. Moussaoui A. E. Bakhouch M. (2022). Crystallographic study, biological assessment and POM/Docking studies of pyrazoles-sulfonamide hybrids (PSH): identification of a combined Antibacterial/Antiviral pharmacophore sites leading to *in-silico* screening the anti-Covid-19 activity. J. Mol. Struct. 1267, 133605. 10.1016/j.molstruc.2022.133605 35782312 PMC9237569

[B11] Channar P. A. Afzal S. Ejaz S. A. Saeed A. Larik F. A. Mahesar P. A. (2018). Exploration of carboxy pyrazole derivatives: synthesis, alkaline phosphatase, nucleotide pyrophosphatase/phosphodiesterase and nucleoside triphosphate diphosphohydrolase inhibition studies with potential anticancer profile. Eur. J. Med. Chem. 156, 461–478. 10.1016/j.ejmech.2018.07.002 30015078

[B12] Dera A. A. Zaib S. Hussain N. Rana N. Javed H. Khan I. (2023). Identification of potent inhibitors targeting EGFR and HER3 for effective treatment of chemoresistance in non-small cell lung cancer. Molecules 28 (12), 4850. 10.3390/molecules28124850 37375404 PMC10304665

[B13] Derosa G. Maffioli P. (2012). *α*-Glucosidase inhibitors and their use in clinical practice. Arch. Med. Sci. 8 (5), 899–906. 10.5114/aoms.2012.31621 23185202 PMC3506243

[B14] Dhameja M. Gupta P. (2019). Synthetic heterocyclic candidates as promising α-glucosidase inhibitors: an overview. Eur. J. Med. Chem. 176, 343–377. 10.1016/j.ejmech.2019.04.025 31112894

[B15] Dym O. Eisenberg D. Yeates T. O. (2006). VERIFY3D. In: Rossmann M. G. Arnold E., editors. International tables for crystallography, crystallography of biological macromolecules, Springer, China, F vol. p 521. 10.1107/97809553602060000106

[B16] Ebenezer O. Shapi M. Tuszynski J. A. (2022). A review of the recent development in the synthesis and biological evaluations of pyrazole derivatives. Biomedicines 10 (5), 1124. 10.3390/biomedicines10051124 35625859 PMC9139179

[B17] Eisenberg D. Lüthy R. Bowie J. U. (1997). VERIFY3D: assessment of protein models with three-dimensional profiles. Methods Enzymol. 277, 396–404. 10.1016/s0076-6879(97)77022-8 9379925

[B18] Faisal M. Saeed A. Hussain S. Dar P. Larik F. A. (2019). Recent developments in synthetic chemistry and biological activities of pyrazole derivatives. J. Chem. Sci. 131, 70–30. 10.1007/s12039-019-1646-1

[B19] Fang Z. Fang W. Liu J. Hong Y. Peng H. Zhang X. (2010). Cloning and characterization of a β-glucosidase from marine microbial metagenome with excellent glucose tolerance. J. Microbiol. Biotechnol. 20 (9), 1351–1358. 10.4014/jmb.1003.03011 20890102

[B20] Gehlot P. Kumar S. Kumar Vyas V. Singh Choudhary B. Sharma M. Malik R. (2022). Guanidine-based β amyloid precursor protein cleavage enzyme 1 (BACE-1) inhibitors for the Alzheimer's disease (AD): a review. Bioorg. Med. Chem. 74, 117047. 10.1016/j.bmc.2022.117047 36265268

[B21] Ghosh A. K. Brindisi M. Shahabi D. Chapman M. E. Mesecar A. D. (2020). Drug development and medicinal chemistry efforts toward SARS-coronavirus and covid-19 therapeutics. ChemMedChem 15 (11), 907–932. 10.1002/cmdc.202000223 32324951 PMC7264561

[B22] Gispen W. H. Biessels G. J. (2000). Cognition and synaptic plasticity in diabetes mellitus. Trends Neurosci. 23 (11), 542–549. 10.1016/s0166-2236(00)01656-8 11074263

[B23] Hasan M. A. Khan M. A. Datta A. Mazumder M. H. Hossain M. U. (2015). A comprehensive immunoinformatics and target site study revealed the corner-stone toward Chikungunya virus treatment. Mol. Immunol. 65 (1), 189–204. 10.1016/j.molimm.2014.12.013 25682054 PMC7172456

[B24] Hasan M. A. Mazumder M. H. Chowdhury A. S. Datta A. Khan M. A. (2015). Molecular-docking study of malaria drug target enzyme transketolase in Plasmodium falciparum 3D7 portends the novel approach to its treatment. Source Code Biol. Med. 10, 7. 10.1186/s13029-015-0037-3 26089981 PMC4472393

[B25] Ibrar A. Zaib S. Jabeen F. Iqbal J. Saeed A. (2016). Unraveling the alkaline phosphatase inhibition, anticancer, and antileishmanial potential of coumarin-triazolothiadiazine hybrids: design, synthesis, and molecular docking analysis. Arch. Pharm. 349 (7), 553–565. 10.1002/ardp.201500392 27214743

[B26] Joshi S. R. Standl E. Tong N. Shah P. Kalra S. Rathod R. (2015). Therapeutic potential of α-glucosidase inhibitors in type 2 diabetes mellitus: an evidence-based review. Expert Opin. Pharmacother. 16 (13), 1959–1981. 10.1517/14656566.2015.1070827 26255950

[B27] Kakkar S. Narasimhan B. (2019). A comprehensive review on biological activities of oxazole derivatives. BMC Chem. 13 (1), 16. 10.1186/s13065-019-0531-9 31384765 PMC6661760

[B28] Kausar N. Ullah S. Khan M. A. Zafar H. Wahab Atia-Tul Choudhary M. I. (2021). Celebrex derivatives: synthesis, α-glucosidase inhibition, crystal structures and molecular docking studies. Bioorg. Chem. 106, 104499. 10.1016/j.bioorg.2020.104499 33288319

[B29] Kazmi M. Zaib S. Amjad S. T. Khan I. Ibrar A. Saeed A. (2017). Exploration of aroyl/heteroaroyl iminothiazolines featuring 2,4,5-trichlorophenyl moiety as a new class of potent, selective, and *in vitro* efficacious glucosidase inhibitors. Bioorg. Chem. 74, 134–144. 10.1016/j.bioorg.2017.07.012 28780150

[B30] Khan F. A. Mushtaq S. Naz S. Farooq U. Zaidi A. Bukhari S. M. (2018). Sulfonamides as potential bioactive scaffolds. Curr. Org. Chem. 22 (8), 818–830. 10.2174/1385272822666180122153839

[B31] Khan M. F. Alam M. M. Verma G. Akhtar W. Akhter M. Shaquiquzzaman M. (2016). The therapeutic voyage of pyrazole and its analogs: a review. Eur. J. Med. Chem. 120, 170–201. 10.1016/j.ejmech.2016.04.077 27191614

[B32] Kharroubi A. T. Darwish H. M. (2015). Diabetes mellitus: the epidemic of the century. World J. Diabetes. 6 (6), 850–867. 10.4239/wjd.v6.i6.850 26131326 PMC4478580

[B33] Kobayashi S. Liang Q. (2015). Autophagy and mitophagy in diabetic cardiomyopathy. Biochim. Biophys. Acta. 1852 (2), 252–261. 10.1016/j.bbadis.2014.05.020 24882754

[B34] Kołaczek A. Fusiarz I. Ławecka J. Branowska D. (2014). Biological activity and synthesis of sulfonamide derivatives: a brief review. Chemik 68 (7), 620–628.

[B35] Kong Y. Wang F. Wang J. Liu C. Zhou Y. Xu Z. (2020). Pathological mechanisms linking diabetes mellitus and Alzheimer's disease: the receptor for advanced glycation end products (RAGE). Front. Aging Neurosci. 12, 217. 10.3389/fnagi.2020.00217 32774301 PMC7388912

[B36] Kumar H. V. Kumar P. Rangaswamy J. Sindhu K. U. Naik N. (2015). 5H-Dibenz [b, f] azepine based pyrazole sulphonamides: a privileged platform for probing the antimicrobial and antioxidative properties. Eur. J. Chem. 6 (4), 394–403. 10.5155/eurjchem.6.4.394-403.1297

[B37] Kumar Verma S. Verma R. Xue F. Kumar Thakur P. Girish Y. R. Rakesh K. P. (2020). Antibacterial activities of sulfonyl or sulfonamide containing heterocyclic derivatives and its structure-activity relationships (SAR) studies: a critical review. Bioorg. Chem. 105, 104400. 10.1016/j.bioorg.2020.104400 33128966

[B38] Laskowski R. A. Furnham N. Thornton J. M. (2013). The Ramachandran plot and protein structure validation. Biomol. forms Funct. a celebration 50 years ramachandran map, 62–75. 10.1142/9789814449144_0005

[B39] Lee D. S. Lee J. M. Kim S. U. Chang K. T. Lee S. H. (2007). Ceftezole, a cephem antibiotic, is an alpha-glucosidase inhibitor with *in vivo* anti-diabetic activity. Int. J. Mol. Med. 20 (3), 379–383. 10.3892/ijmm.20.3.379 17671744

[B40] Lee Y. Kim S. Kim J. Y. Arooj M. Kim S. Hwang S. (2014). Binding mode analyses and pharmacophore model development for stilbene derivatives as a novel and competitive class of α-glucosidase inhibitors. PloS One 9 (1), e85827. 10.1371/journal.pone.0085827 24465730 PMC3897524

[B41] Mensah E. Kohner E. (2002). Diagnosis and management of diabetic retinopathy. Top. Endocrinol. 19, 14–18.

[B42] Mishra C. B. Kumari S. Angeli A. Bua S. Buonanno M. Monti S. M. (2018). Discovery of potent anti-convulsant carbonic anhydrase inhibitors: design, synthesis, *in vitro* and *in vivo* appraisal. Eur. J. Med. Chem. 156, 430–443. 10.1016/j.ejmech.2018.07.019 30015076

[B43] Mora-Fernández C. Domínguez-Pimentel V. de Fuentes M. M. Górriz J. L. Martínez-Castelao A. Navarro-González J. F. (2014). Diabetic kidney disease: from physiology to therapeutics. J. Physiol. 592 (18), 3997–4012. 10.1113/jphysiol.2014.272328 24907306 PMC4198010

[B44] Mumtaz A. Majeed A. Zaib S. Ur Rahman S. Hameed S. Saeed A. (2019). Investigation of potent inhibitors of cholinesterase based on thiourea and pyrazoline derivatives: synthesis, inhibition assay and molecular modeling studies. Bioorg. Chem. 90, 103036. 10.1016/j.bioorg.2019.103036 31271943

[B45] Mustafa G. Zia-ur-Rehman M. Sumrra S. H. Ashfaq M. Zafar W. Ashfaq M. (2022). A critical review on recent trends on pharmacological applications of pyrazolone endowed derivatives. J. Mol. Struct. 1262, 133044. 10.1016/j.molstruc.2022.133044

[B46] O'Gara P. T. Kushner F. G. Ascheim D. D. Casey D. E. Jr Chung M. K. de Lemos J. A. (2013). 2013 ACCF/AHA guideline for the management of ST-elevation myocardial infarction: executive summary: a report of the American College of cardiology foundation/American heart association task force on practice guidelines. Circulation 127 (4), 529–555. 10.1161/CIR.0b013e3182742c84 23247303

[B47] Rogach I. M. Reho O. Y. Havrosh N. V. Kachur M. M. (2021). SOCIO–ECONOMIC ASPECT OF TYPE 2 DIABETES IMPACT ON THE LIVES OF PATIENTS. Bull. Exp. Biol. Med. 355, 355–358. 10.29254/2077-4214-2021-1-159-355-358

[B48] Saha S. Pal D. (2020). Role of pyrazole ring in neurological drug Discovery. Pyrazole Prep. Uses, 245–264.

[B49] Santosh Kumar B. Raghavendra Guru Prasad A. Madhu G. Raveendra Reddy P. Ravindranath L. K. (2014). Synthesis and *in silico* studies of pyrrolidine sulfonamide based dipeptides as β-gluscosidase inhibitors. Ann. Pharm. Fr. 72 (4), 256–266. 10.1016/j.pharma.2014.02.002 24997887

[B50] SAR (2023).SeeSAR. Sankt Augustin, Germany: BioSolveIT GmbH. Available at: www.biosolveit.de/SeeSAR.

[B51] Shiro T. Fukaya T. Tobe M. (2015). The chemistry and biological activity of heterocycle-fused quinolinone derivatives: a review. Eur. J. Med. Chem. 97, 397–408. 10.1016/j.ejmech.2014.12.004 25532473

[B52] Shu S. Cai X. Li J. Feng Y. Dai A. Wang J. (2016). Design, synthesis, structure-activity relationships, and docking studies of pyrazole-containing derivatives as a novel series of potent glucagon receptor antagonists. Bioorg. Med. Chem. 24 (12), 2852–2863. 10.1016/j.bmc.2016.04.053 27161876

[B53] Sravika N. Priya S. Divya N. Jyotsna P. M. S. Anusha P. Kudumula N. (2021). Swiss ADME properties screening of the phytochemical compounds present in Bauhinia acuminata. J. Pharmacogn. Phytochem. 10 (4), 411–419. 10.22271/phyto.2021.v10.i4e.14193

[B54] Thillainayagam M. Ramaiah S. Anbarasu A. (2020). Molecular docking and dynamics studies on novel benzene sulfonamide substituted pyrazole-pyrazoline analogues as potent inhibitors of *Plasmodium falciparum* Histo aspartic protease. J. Biomol. Struct. Dyn. 38 (11), 3235–3245. 10.1080/07391102.2019.1654923 31411122

[B55] Tucci S. A. Boyland E. J. Halford J. C. (2010). The role of lipid and carbohydrate digestive enzyme inhibitors in the management of obesity: a review of current and emerging therapeutic agents. Diabetes Metab. Syndr. Obes. 3, 125–143. 10.2147/dmsott.s7005 21437083 PMC3047983

[B56] Tugrak M. Gul H. I. Demir Y. Levent S. Gulcin I. (2021). Synthesis and *in vitro* carbonic anhydrases and acetylcholinesterase inhibitory activities of novel imidazolinone-based benzenesulfonamides. Arch. Pharm. 354 (4), e2000375. 10.1002/ardp.202000375 33283898

[B57] Vazzana N. Ranalli P. Cuccurullo C. Davì G. (2012). Diabetes mellitus and thrombosis. Thromb. Res. 129 (3), 371–377. 10.1016/j.thromres.2011.11.052 22197180

[B58] Veves A. Malik R. A. (2007). Diabetic neuropathy: clinical management (Germany: Humana Press). 10.1007/978-1-59745-311-0

[B59] Zaib S. Rana N. Hussain N. Alrbyawi H. Dera A. A. Khan I. (2023). Designing multi-epitope monkeypox virus-specific vaccine using immunoinformatics approach. J. Infect. Public Health. 16 (1), 107–116. 10.1016/j.jiph.2022.11.033 36508944 PMC9724569

[B60] Zaib S. Rana N. Hussain N. Ogaly H. A. Dera A. A. Khan I. (2023). Identification of potential inhibitors for the treatment of alkaptonuria using an integrated *in silico* computational strategy. Molecules 28 (6), 2623. 10.3390/molecules28062623 36985595 PMC10058836

[B61] Zhao X. Wang Y. Chen R. Li J. Zhou J. Liu C. (2021). Triglyceride glucose index combined with plaque characteristics as a novel biomarker for cardiovascular outcomes after percutaneous coronary intervention in ST-elevated myocardial infarction patients: an intravascular optical coherence tomography study. Cardiovasc. Diabetol. 20 (1), 131. 10.1186/s12933-021-01321-7 34183007 PMC8240222

