# Supplementary material for: Acyl pyrazole sulfonamides as new antidiabetic agents: synthesis, glucosidase inhibition studies, and molecular docking analysis
Source: Front Chem. 2024 Apr 17;12:1380523. doi: 10.3389/fchem.2024.1380523 (PMC11061460; doi:10.3389/fchem.2024.1380523)

Supplementary Material

Acyl Pyrazole Sulfonamides as New Antidiabetic Agents: Synthesis, Glucosidase Inhibition Studies and Molecular Docking Analysis

**Atteeque Ahmed^1^, Sumera Zaib^2^*, Mashooq Ahmad Bhat^3^, Aamer Saeed^1^*, Muhammad Zain Altaf^2^, Fatima Tuz Zahra^1^, Ghulam Shabir^1^, Nehal Rana^2^, Imtiaz Khan^4^**

^1^Department of Chemistry, Quaid-I-Azam University, Islamabad 45320, Pakistan

^2^Department of Basic and Applied Chemistry, Faculty of Science and Technology, University of Central Punjab, Lahore 54590, Pakistan

^3^Department of Pharmaceutical Chemistry, College of Pharmacy, King Saud University, Riyadh 11451, Saudi Arabia

^4^Department of Chemistry and Manchester Institute of Biotechnology, The University of Manchester, 131 Princess Street, Manchester M1 7DN, United Kingdom

*** Correspondence:** Prof. Dr. Aamer Saeed (asaeed@qau.edu.pk); Dr Sumera Zaib ([sumera.zaib@ucp.edu.pk](mailto:sumera.zaib@ucp.edu.pk))

*(E)-4-((2,4-Dioxopentan-3-yl)diazenyl)benzenesulfonamide (****3****)*


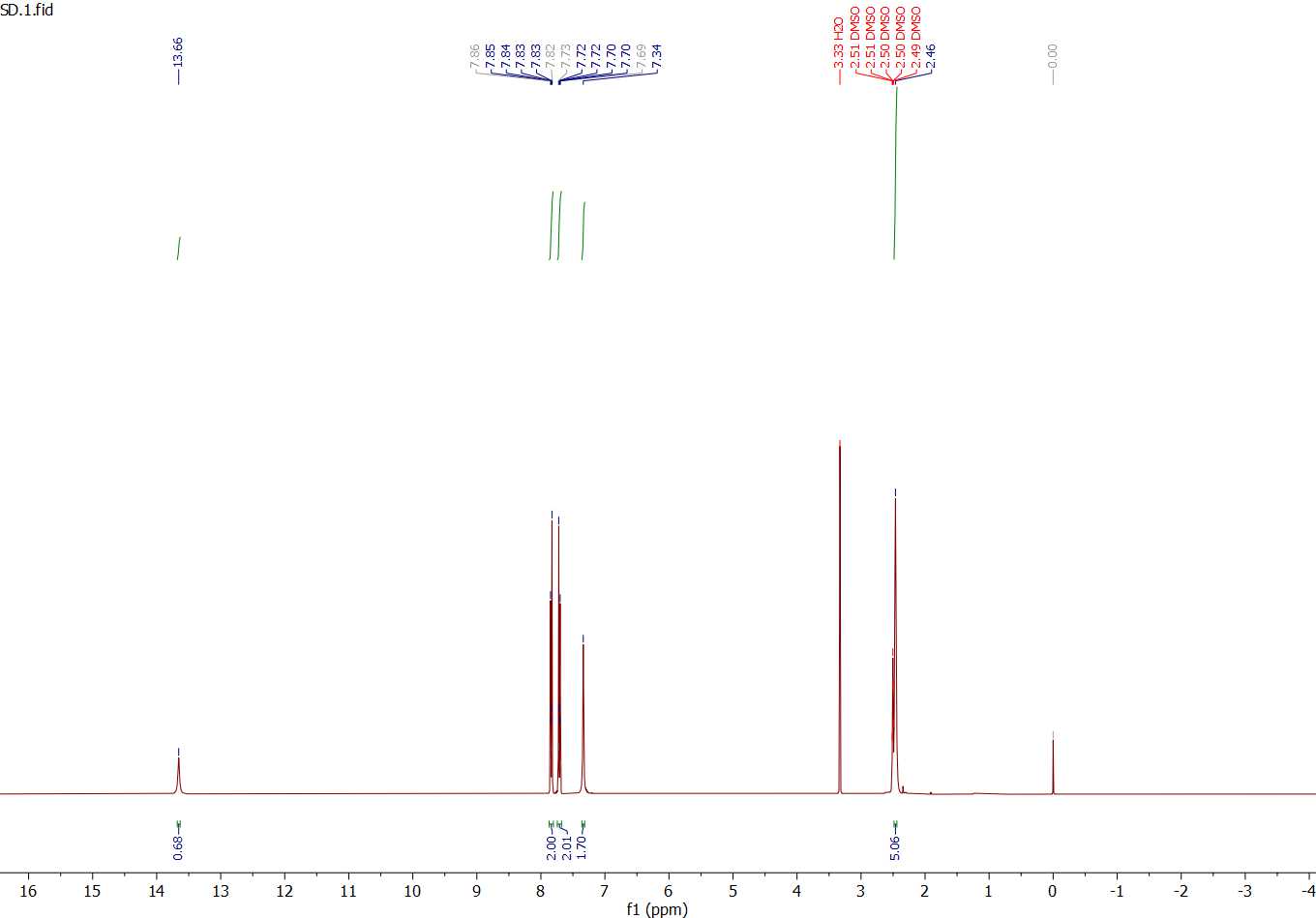


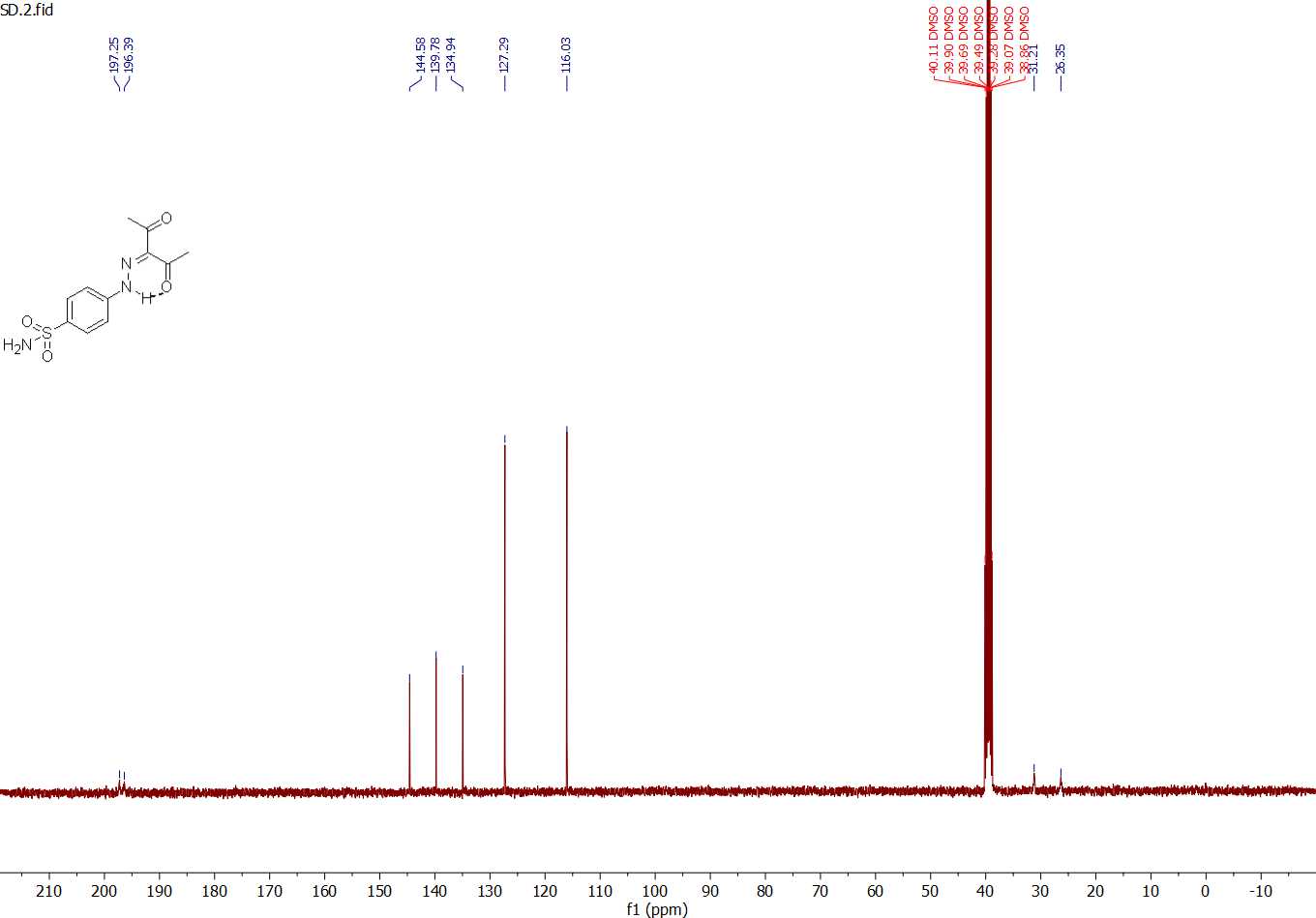


*(E)-4-((1-(4-Chlorobenzoyl)-3,5-dimethyl-1H-pyrazol-4-yl)diazenyl)benzenesulfonamide (****5a****)*


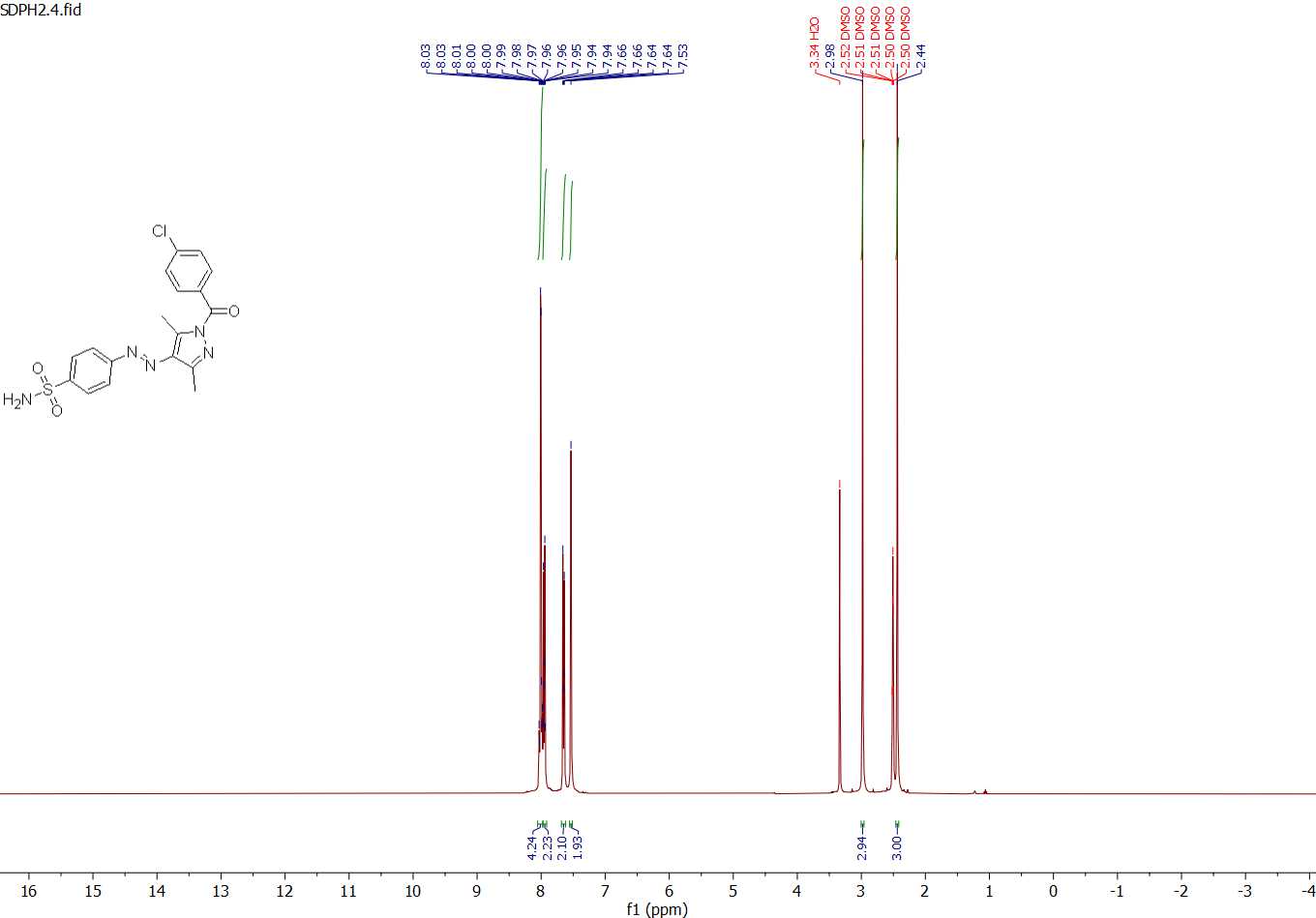


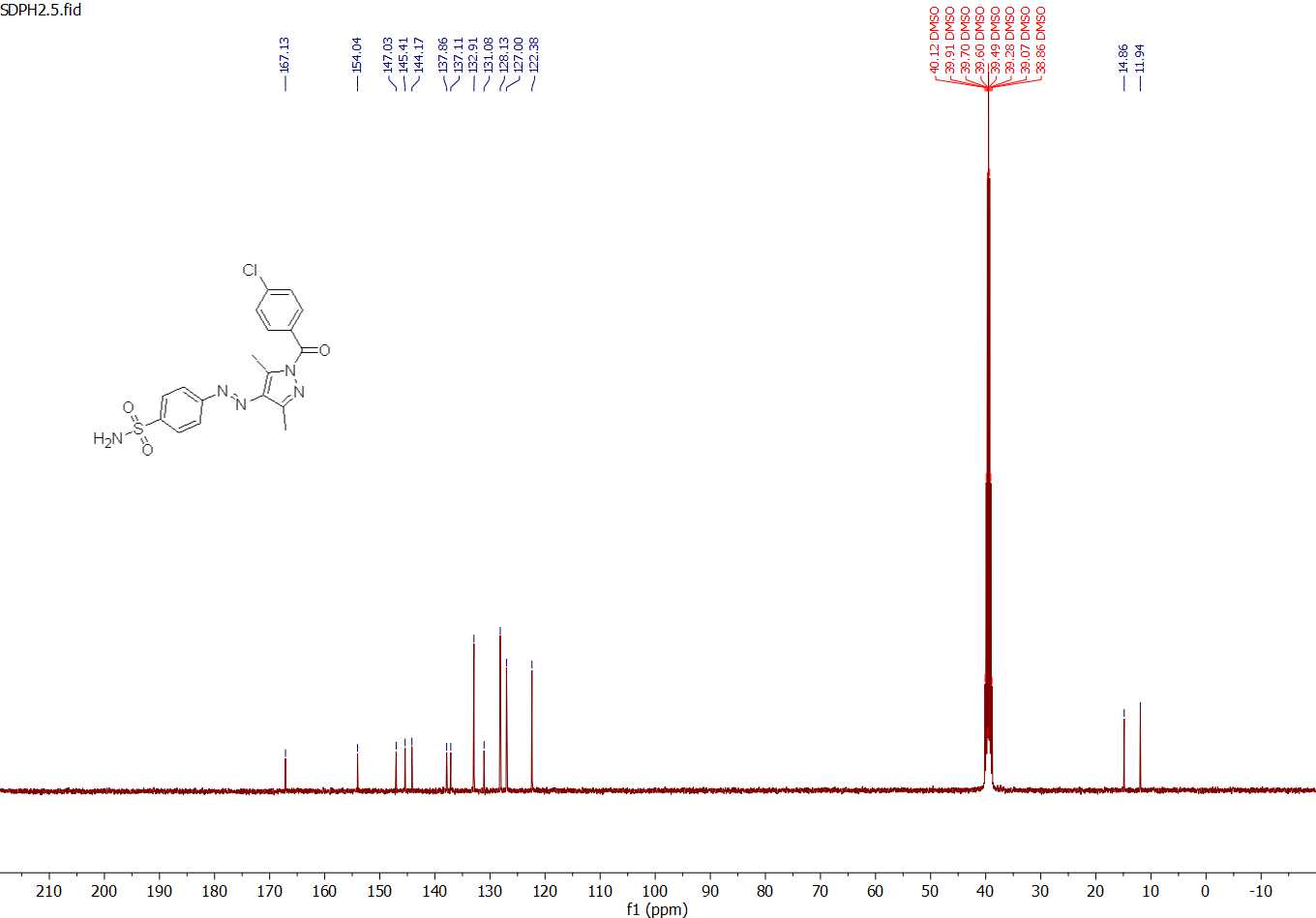


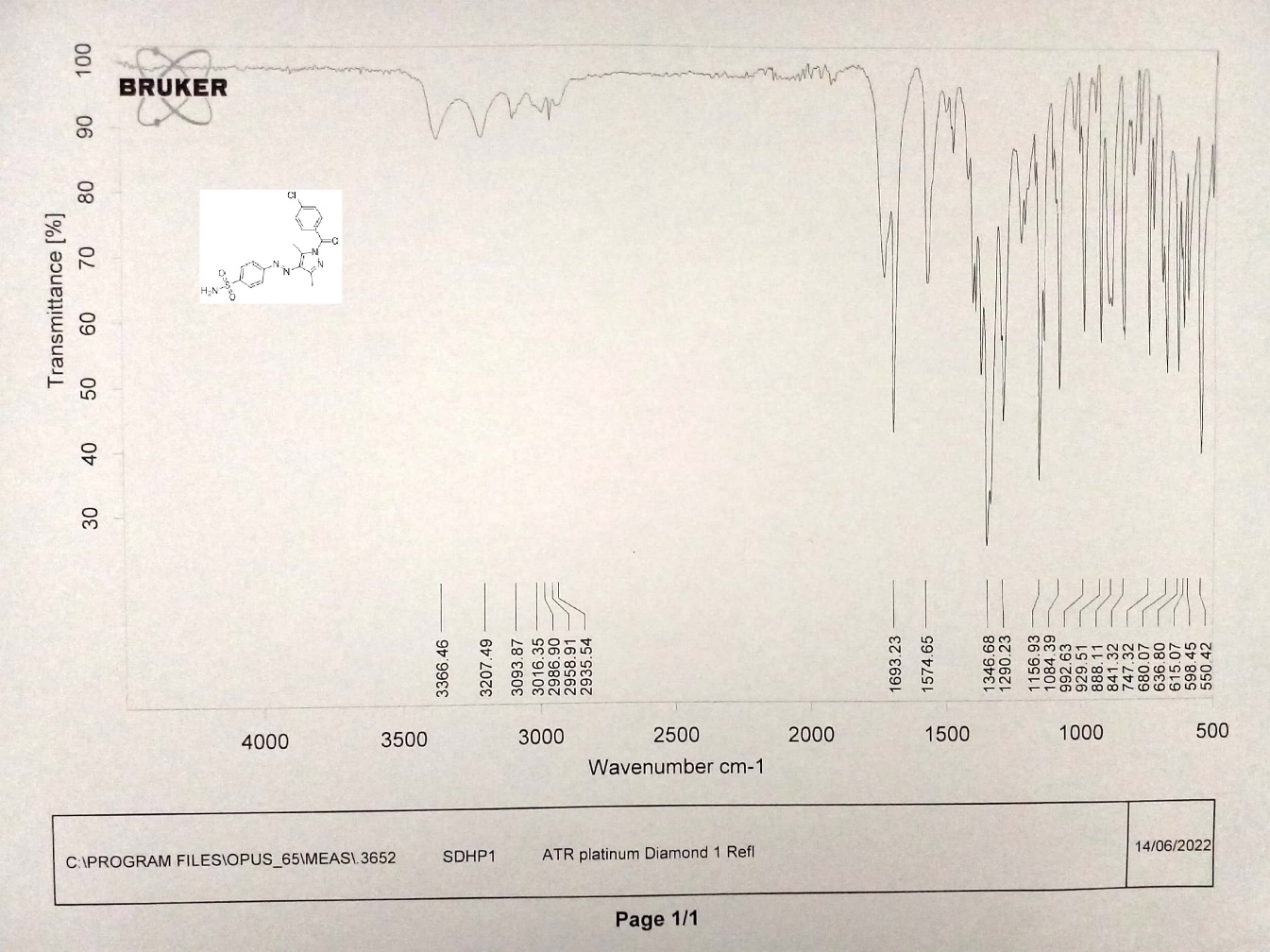


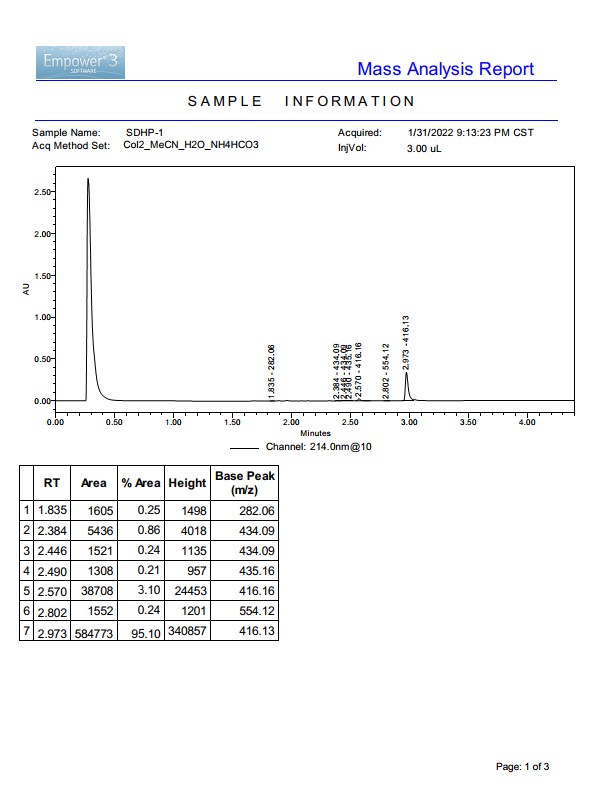

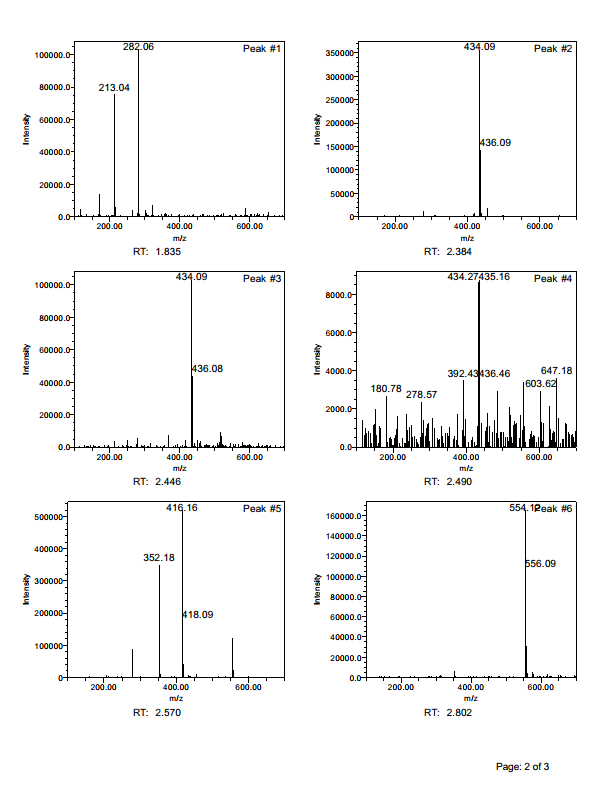

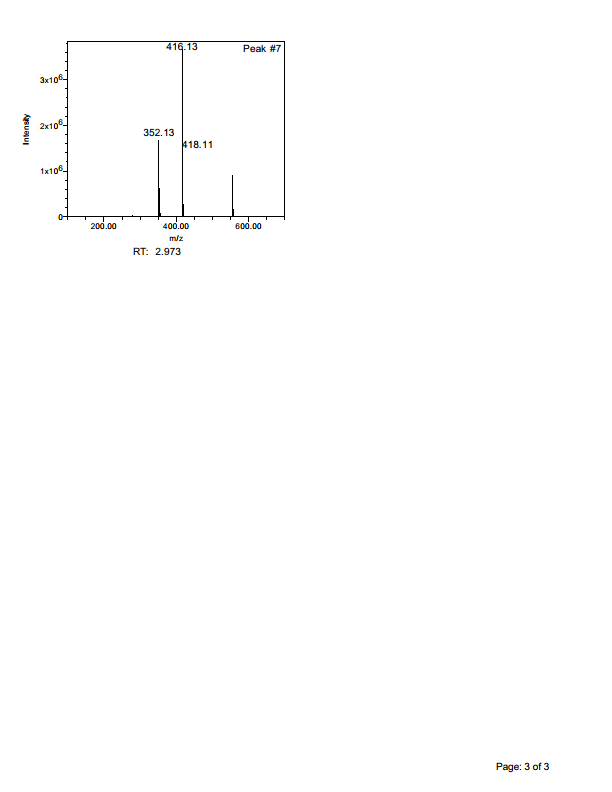


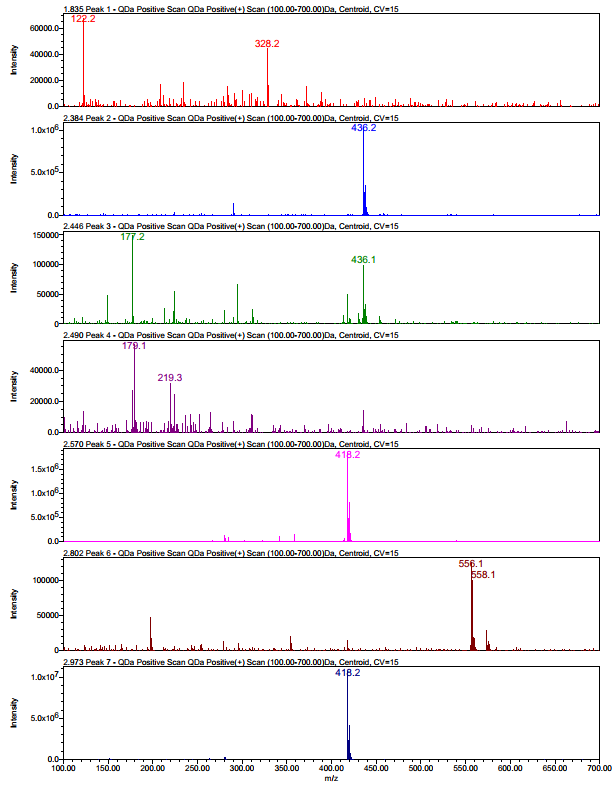


*(E)-4-((1-(3-Chlorobenzoyl)-3,5-dimethyl-1H-pyrazol-4-yl)diazenyl)benzenesulfonamide (****5b****)*


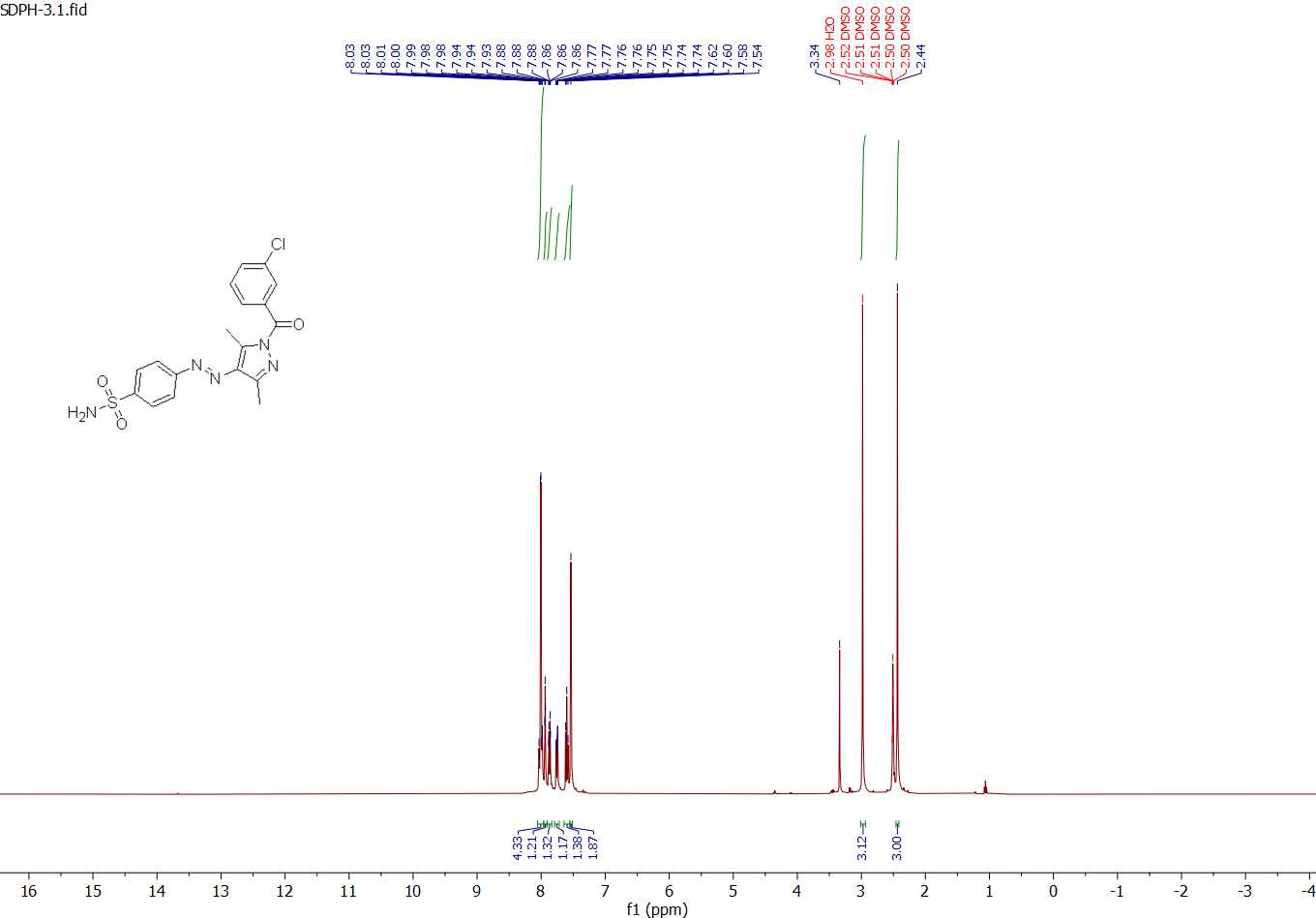


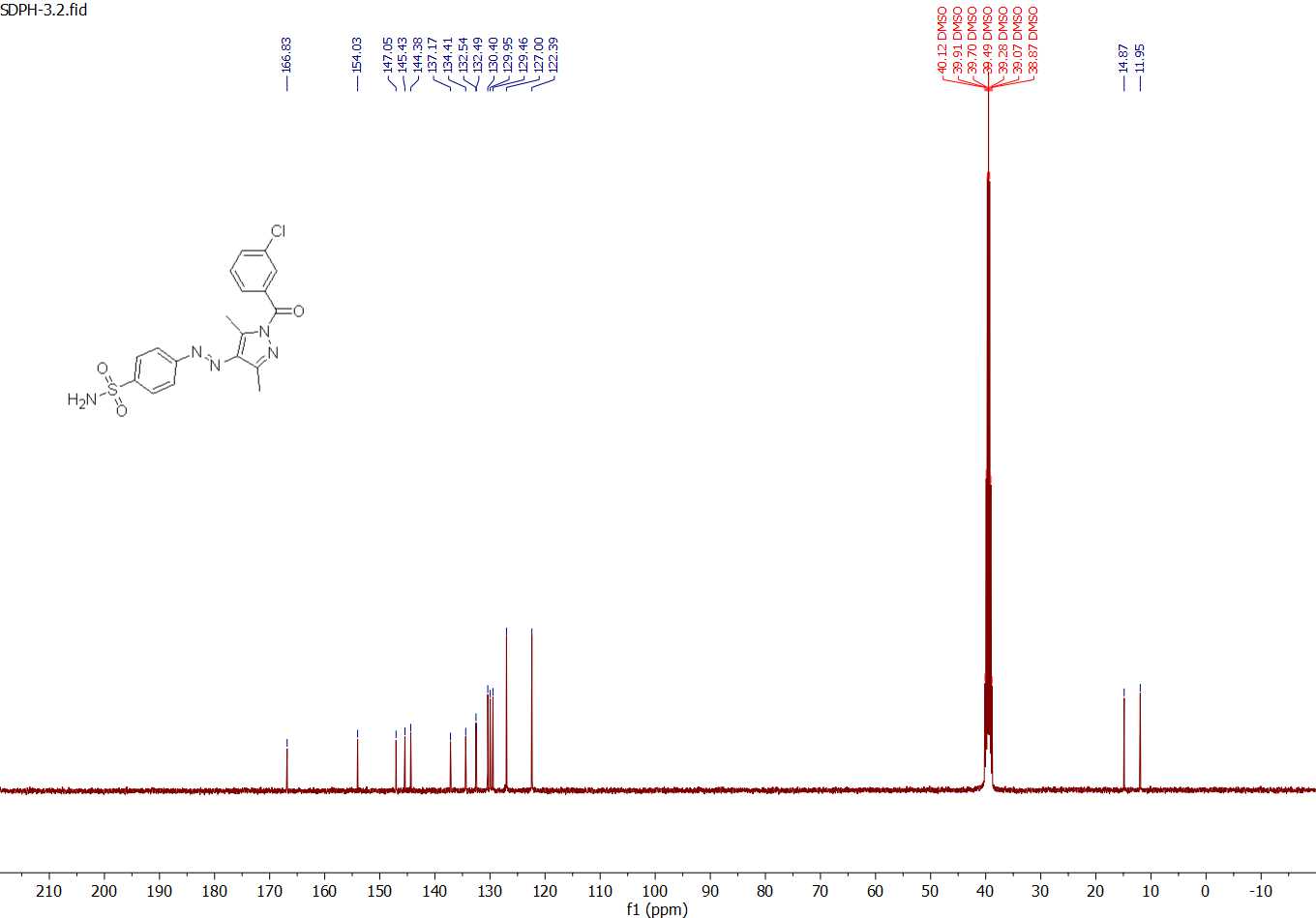


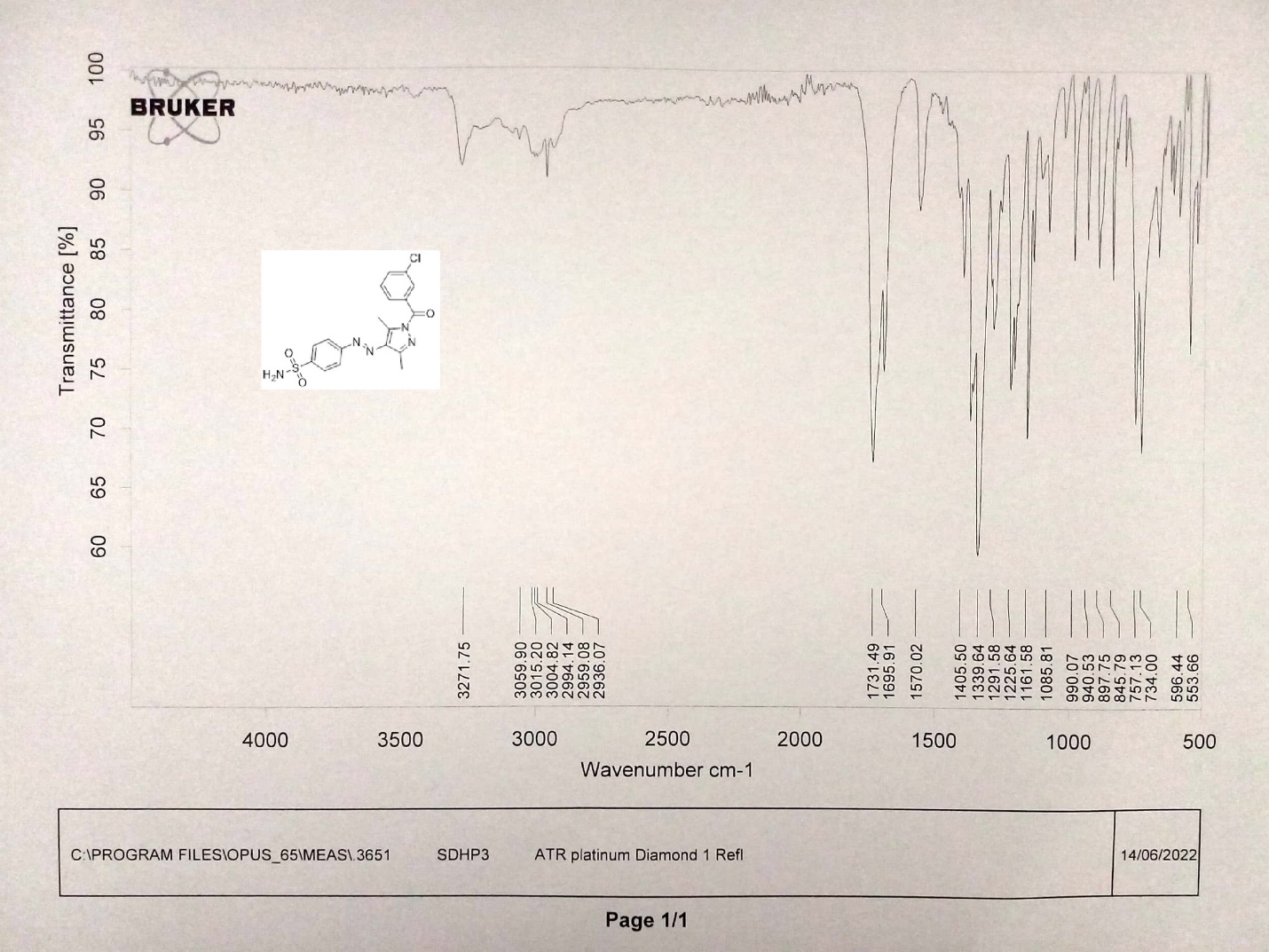


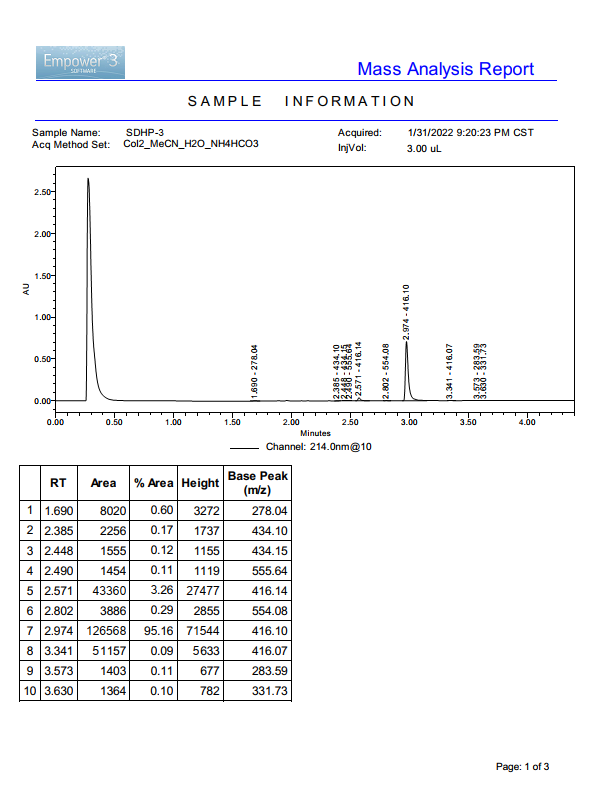


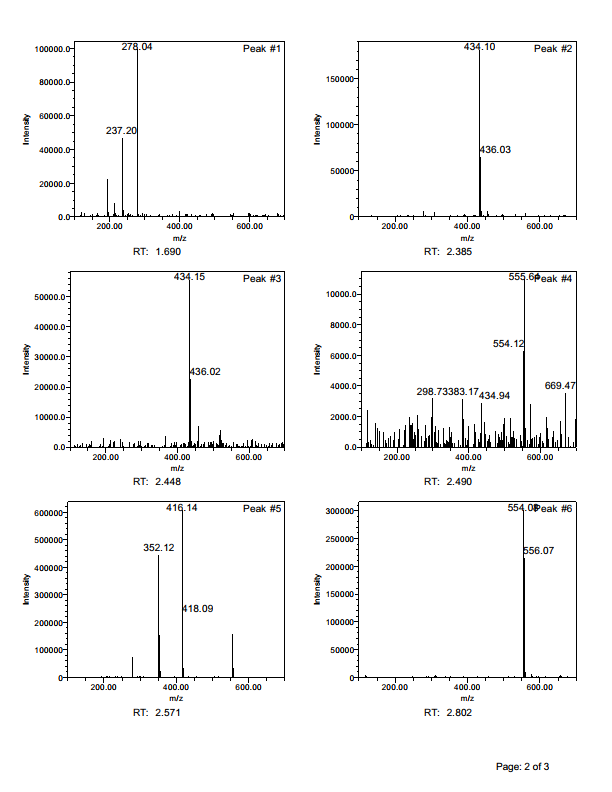


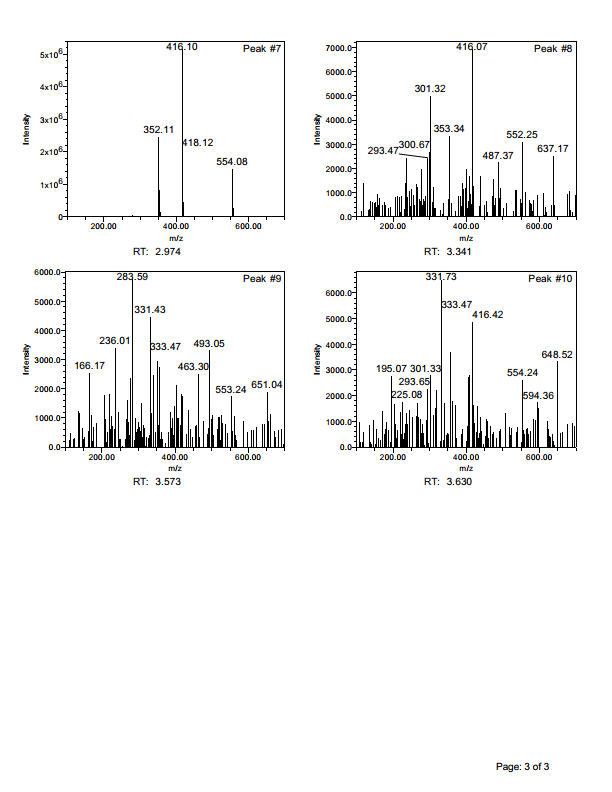


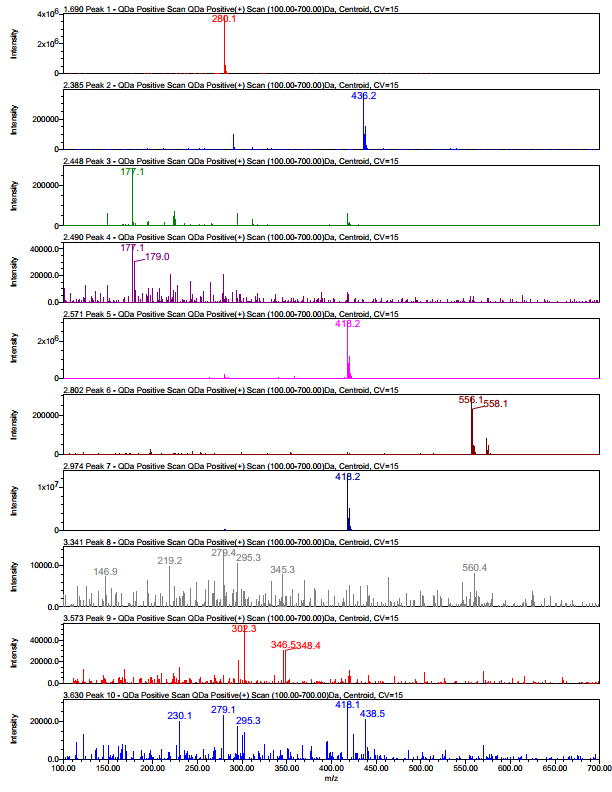


*(E)-4-((1-(4-Methoxybenzoyl)-3,5-dimethyl-1H-pyrazol-4-yl)diazenyl)benzenesulfonamide (****5c****)*


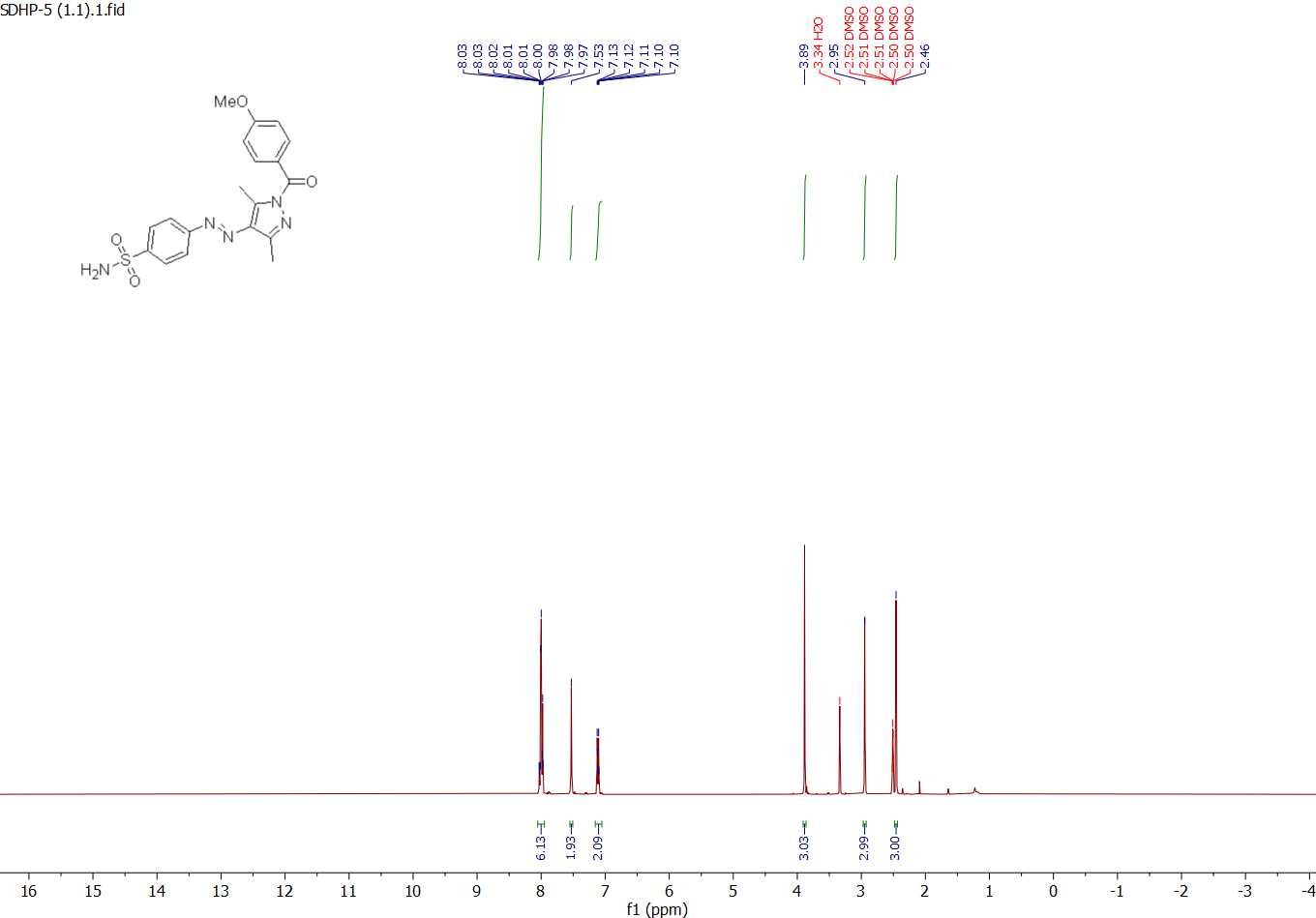


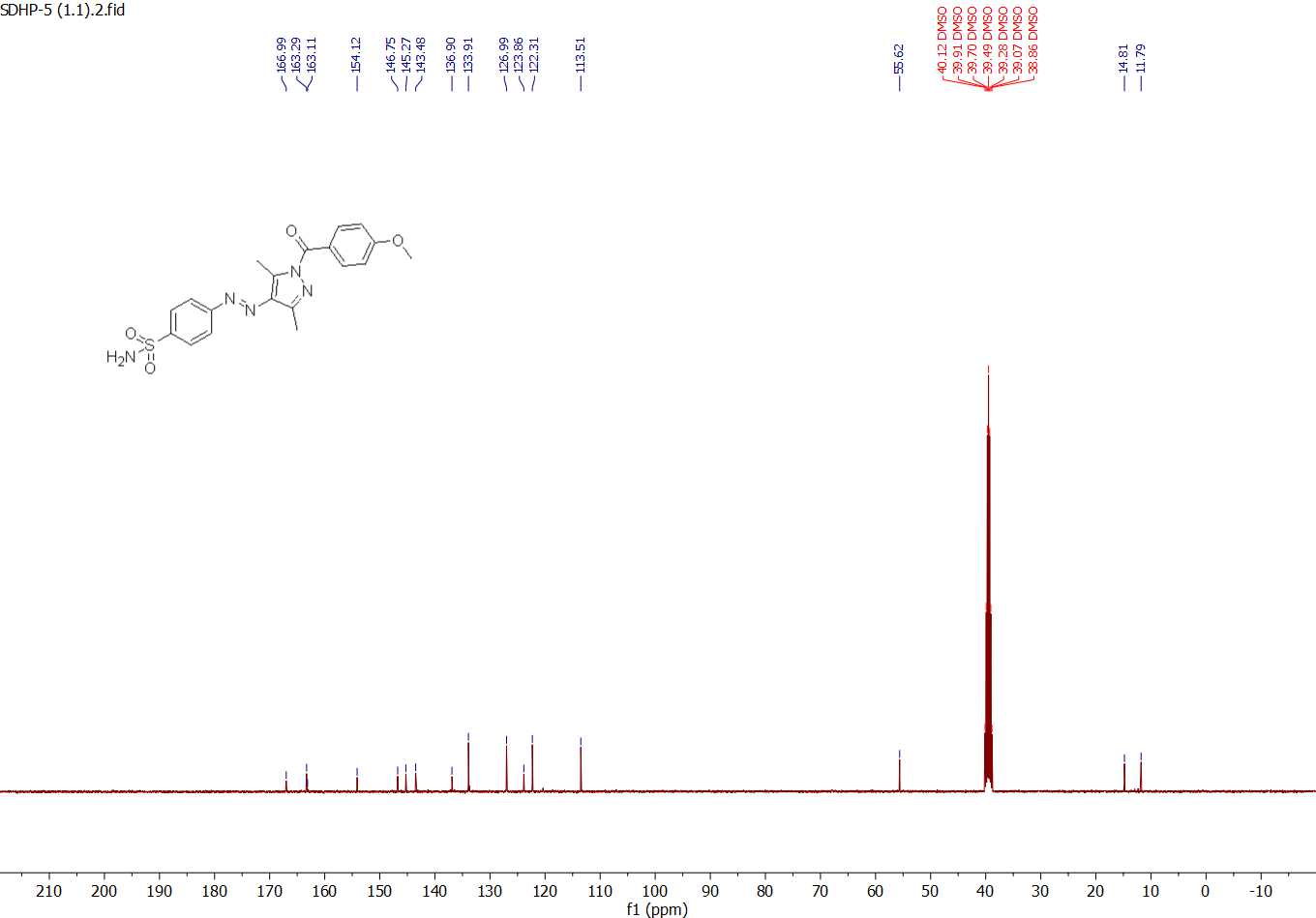


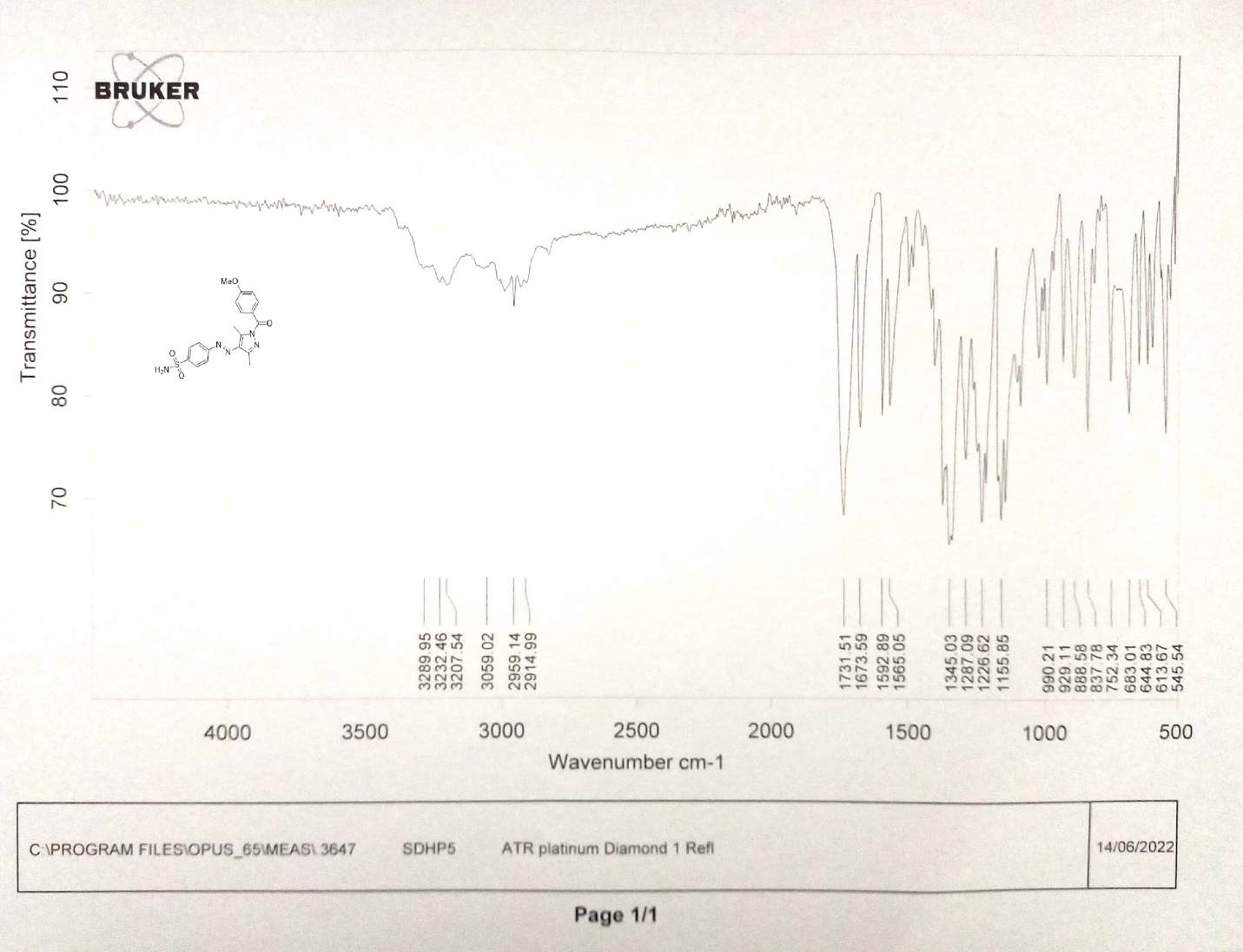


*
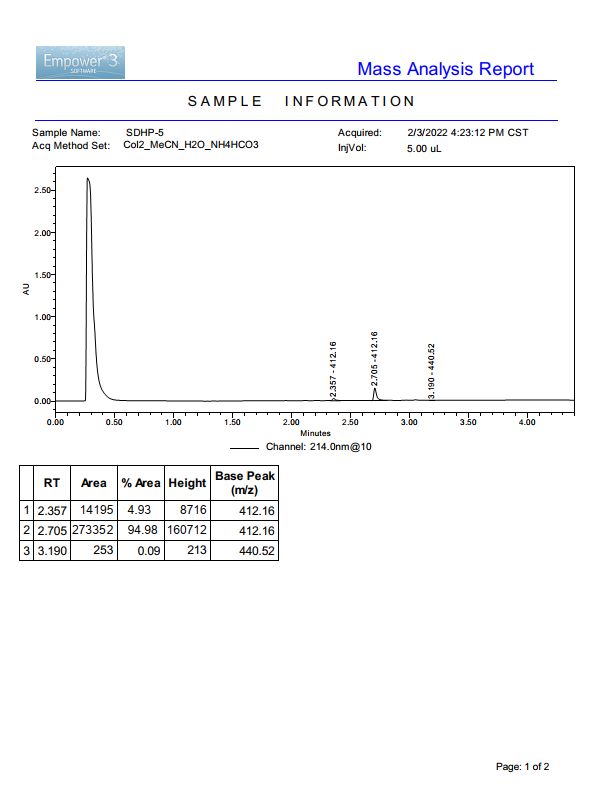
*

*
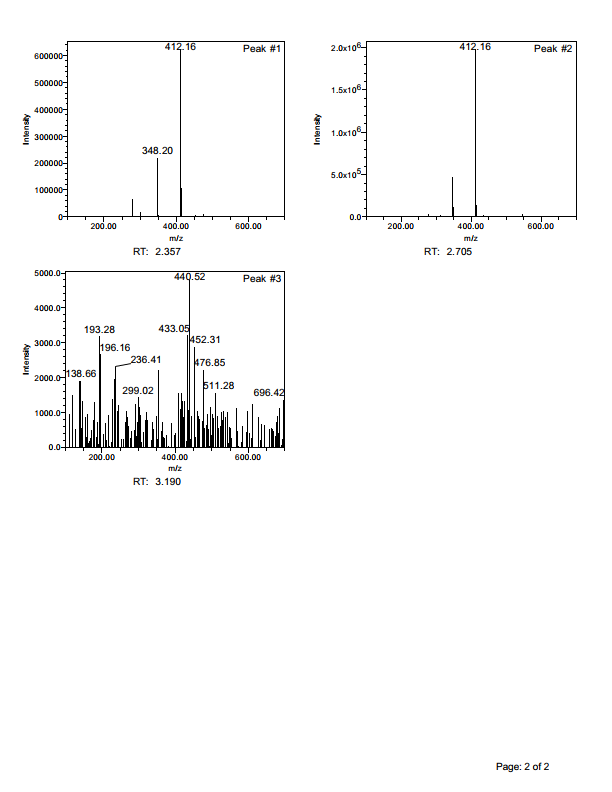
*

*
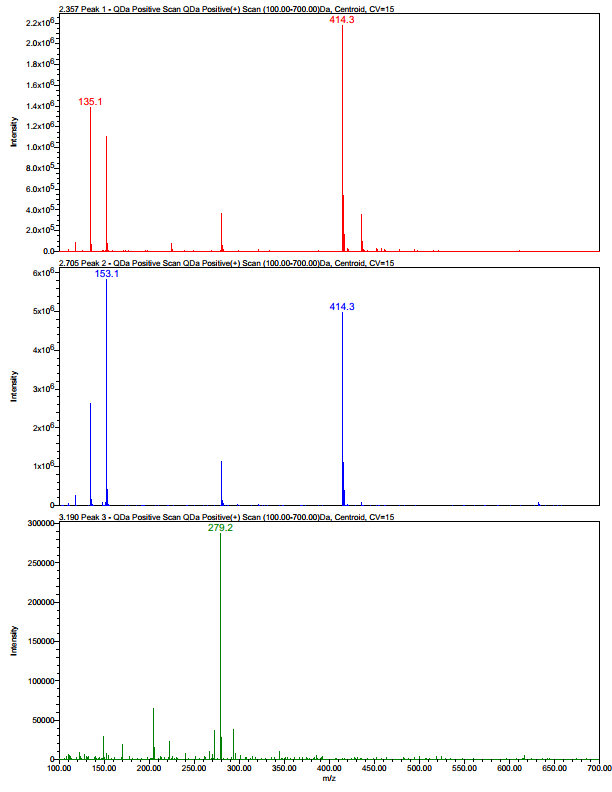
*

*(E)-4-((1-(3,4-Dimethoxybenzoyl)-3,5-dimethyl-1H-pyrazol-4-yl)diazenyl)benzenesulfonamide (****5d****)*


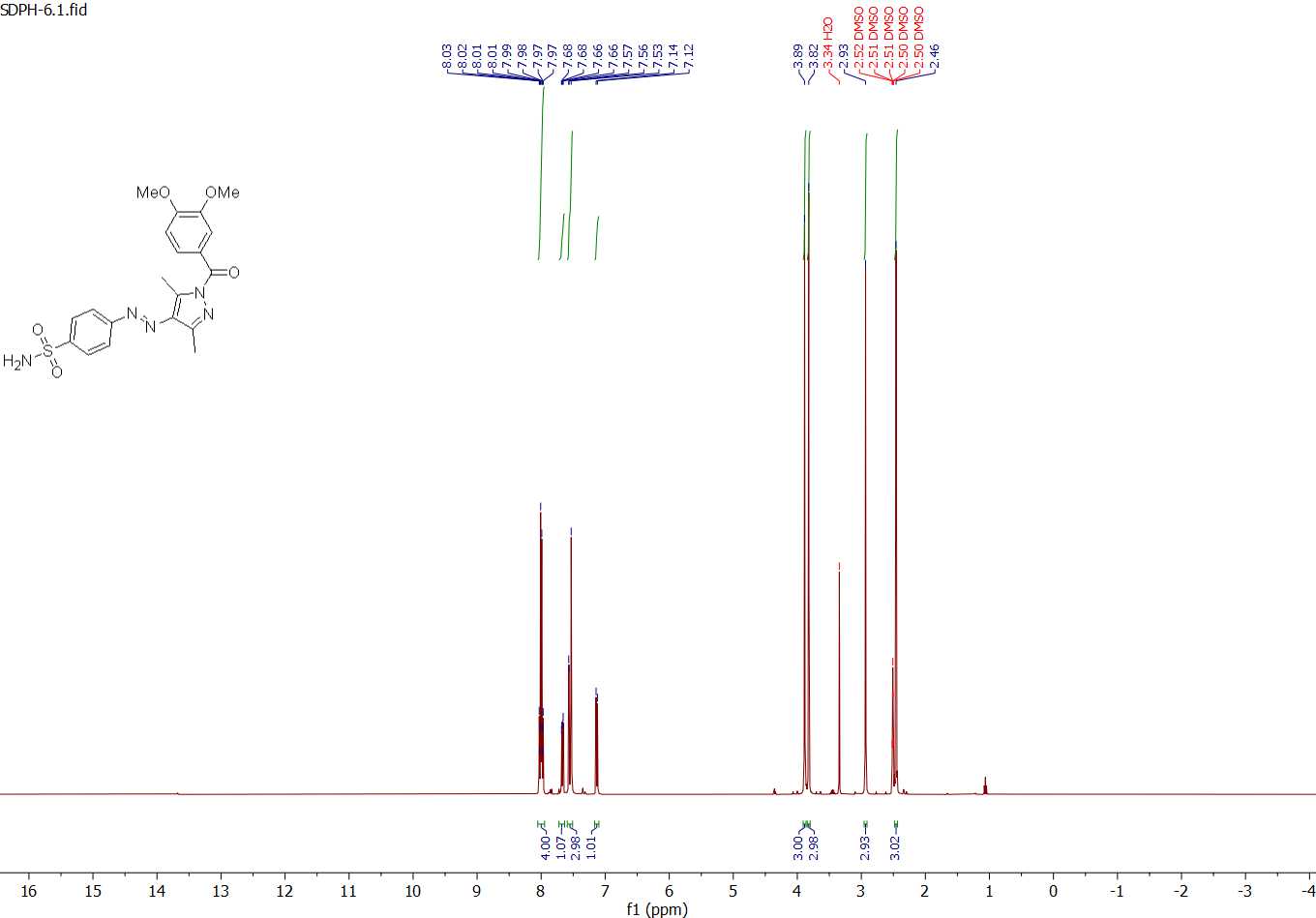

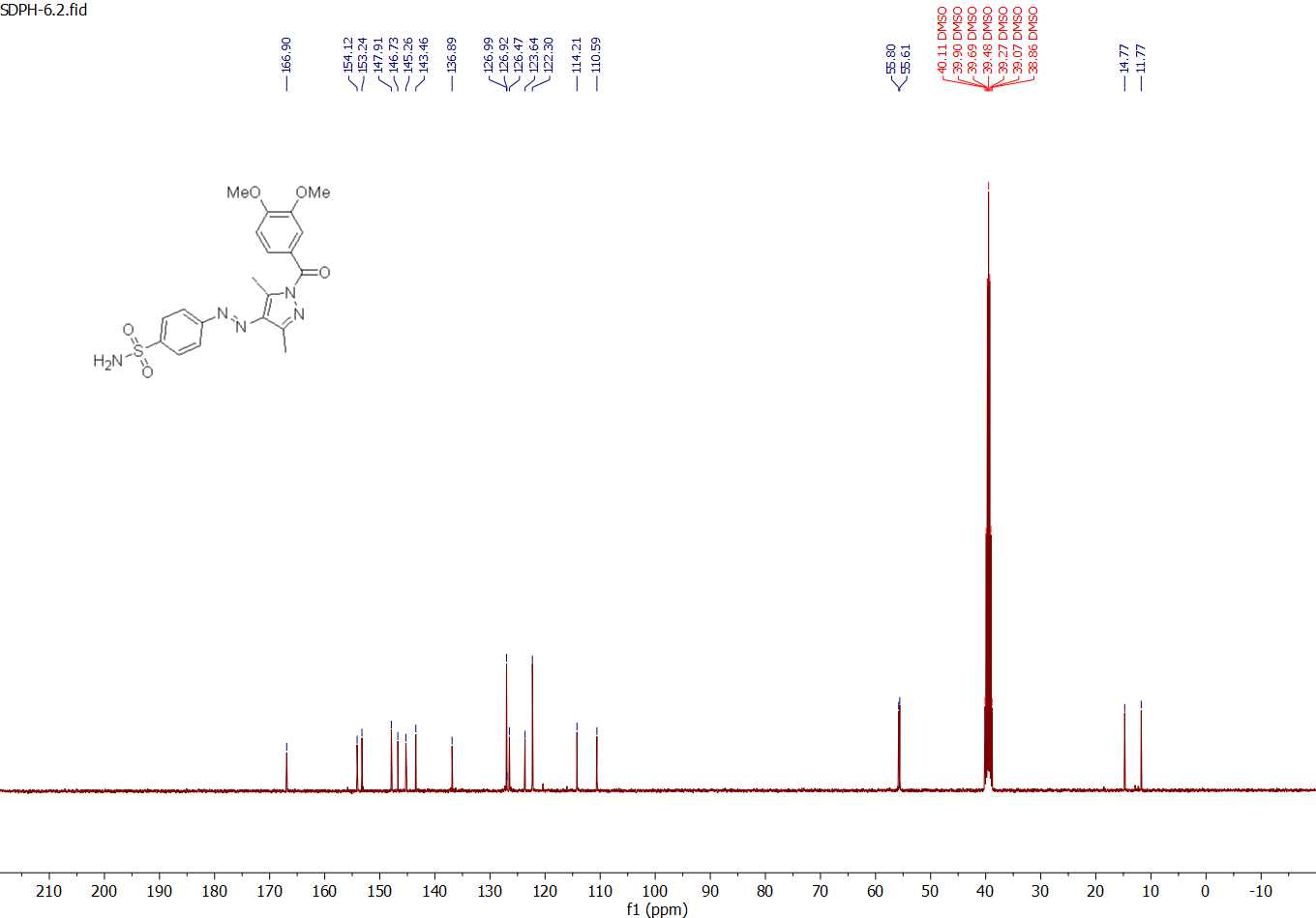


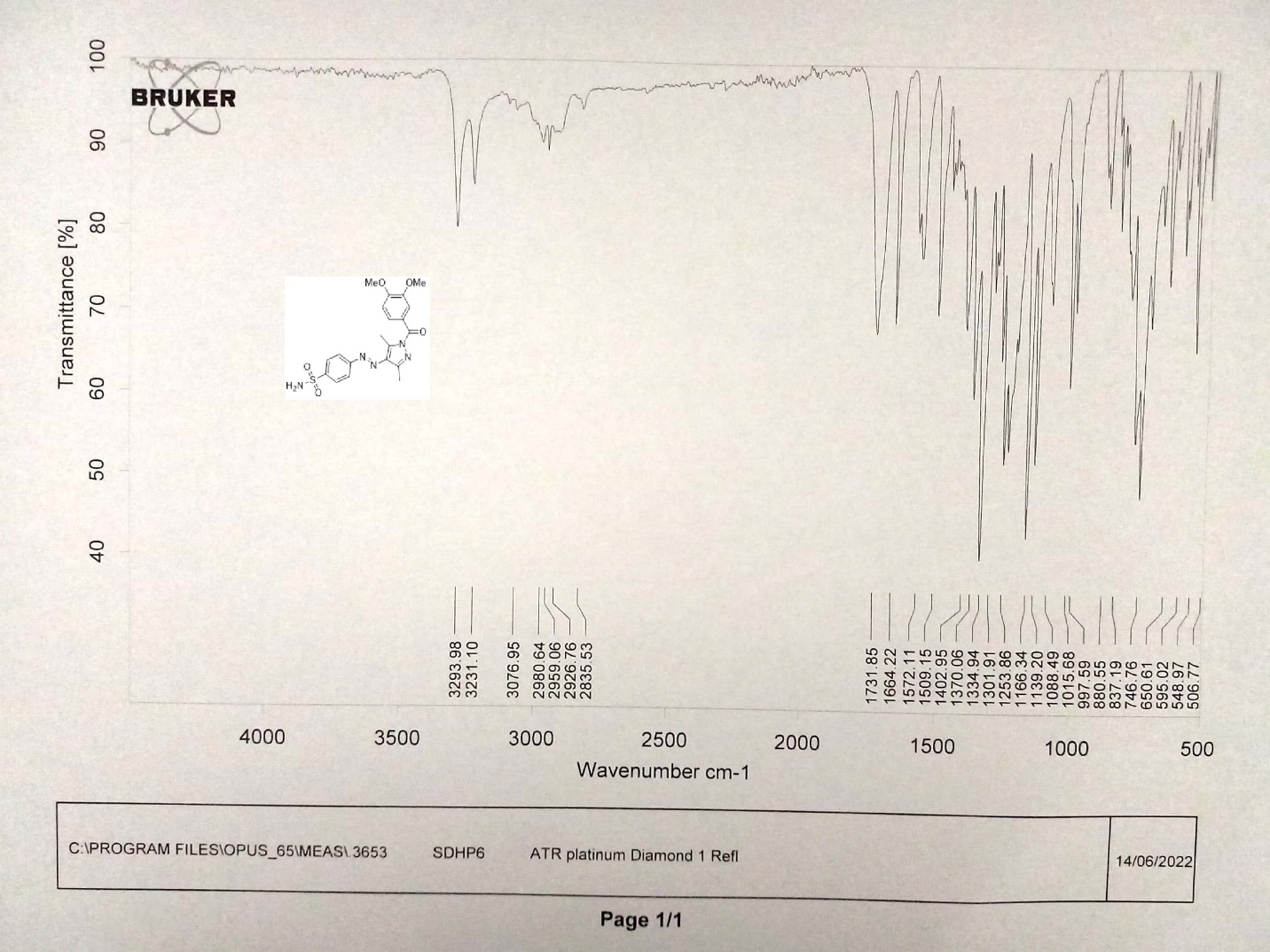


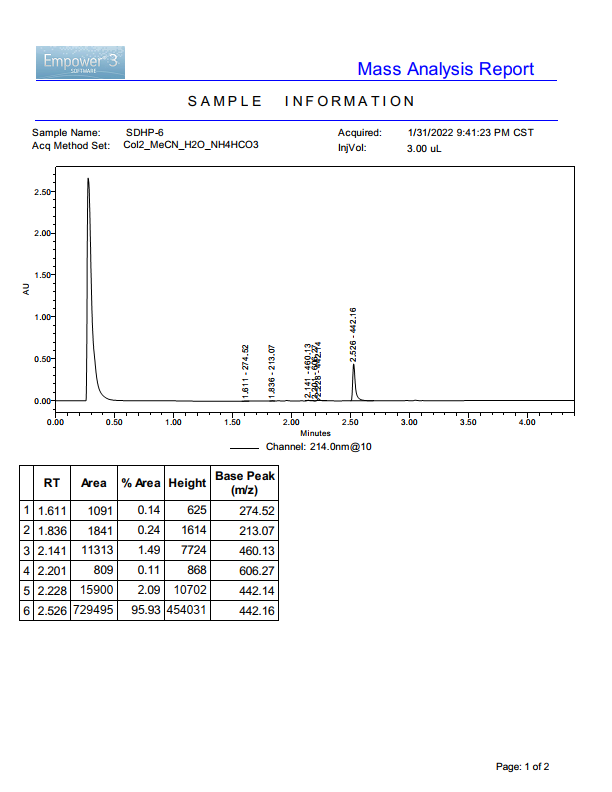


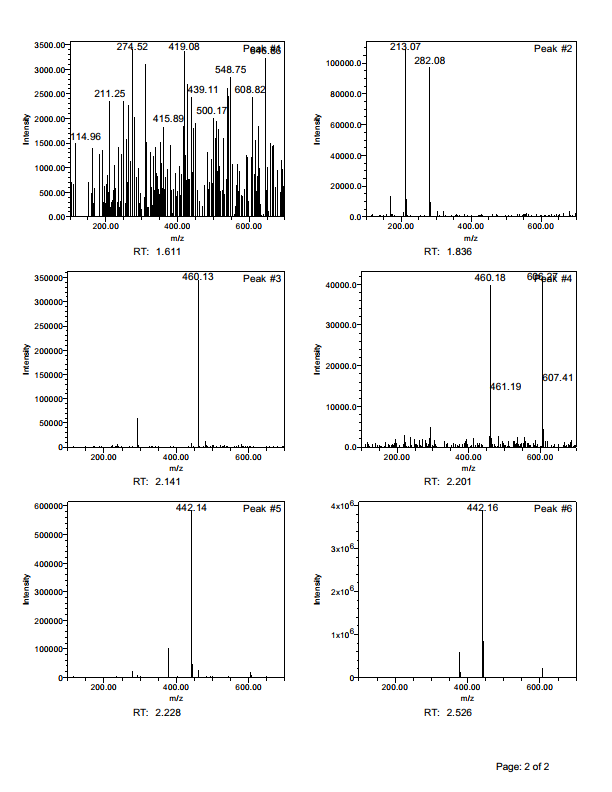


*
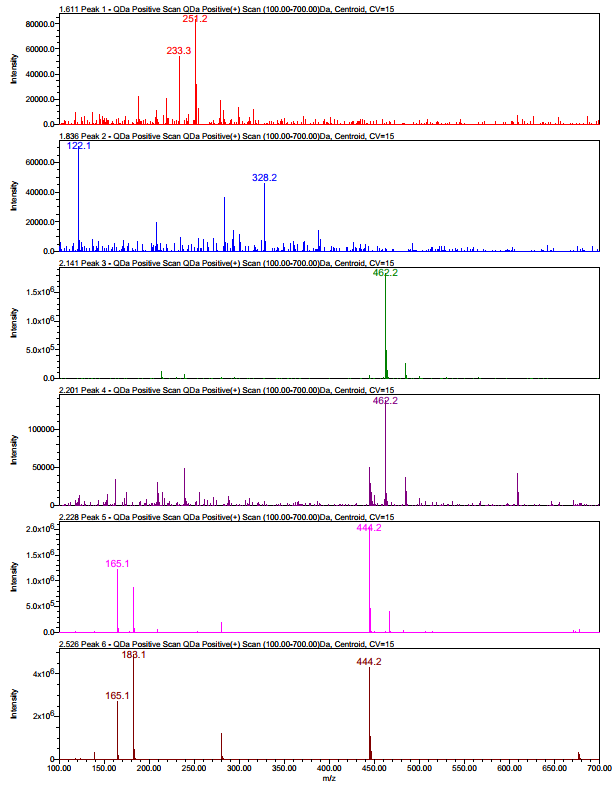
*

*(E)-4-((3,5-Dimethyl-1-(4-nitrobenzoyl)-1H-pyrazol-4-yl)diazenyl)benzenesulfonamide (****5e****)*


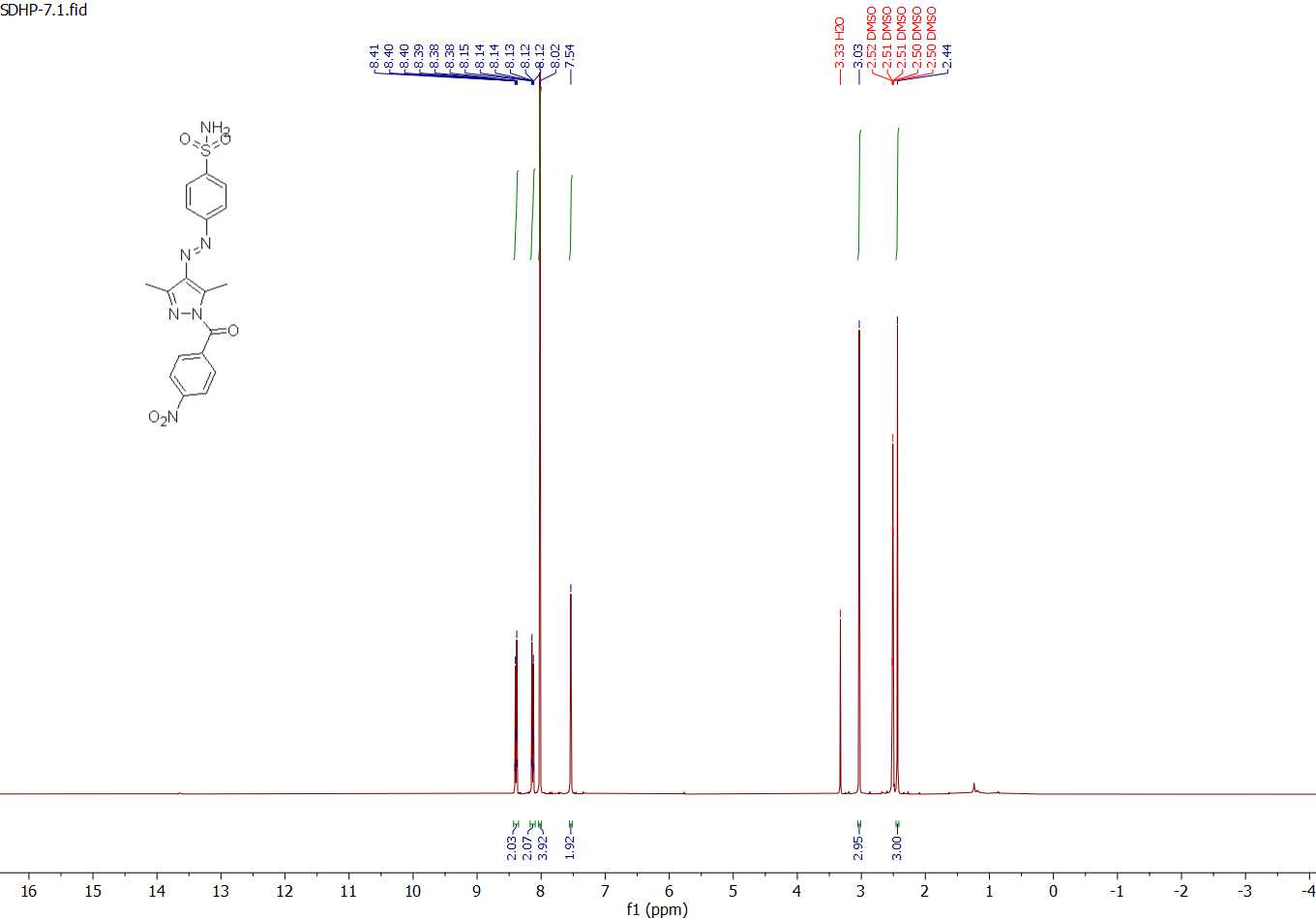

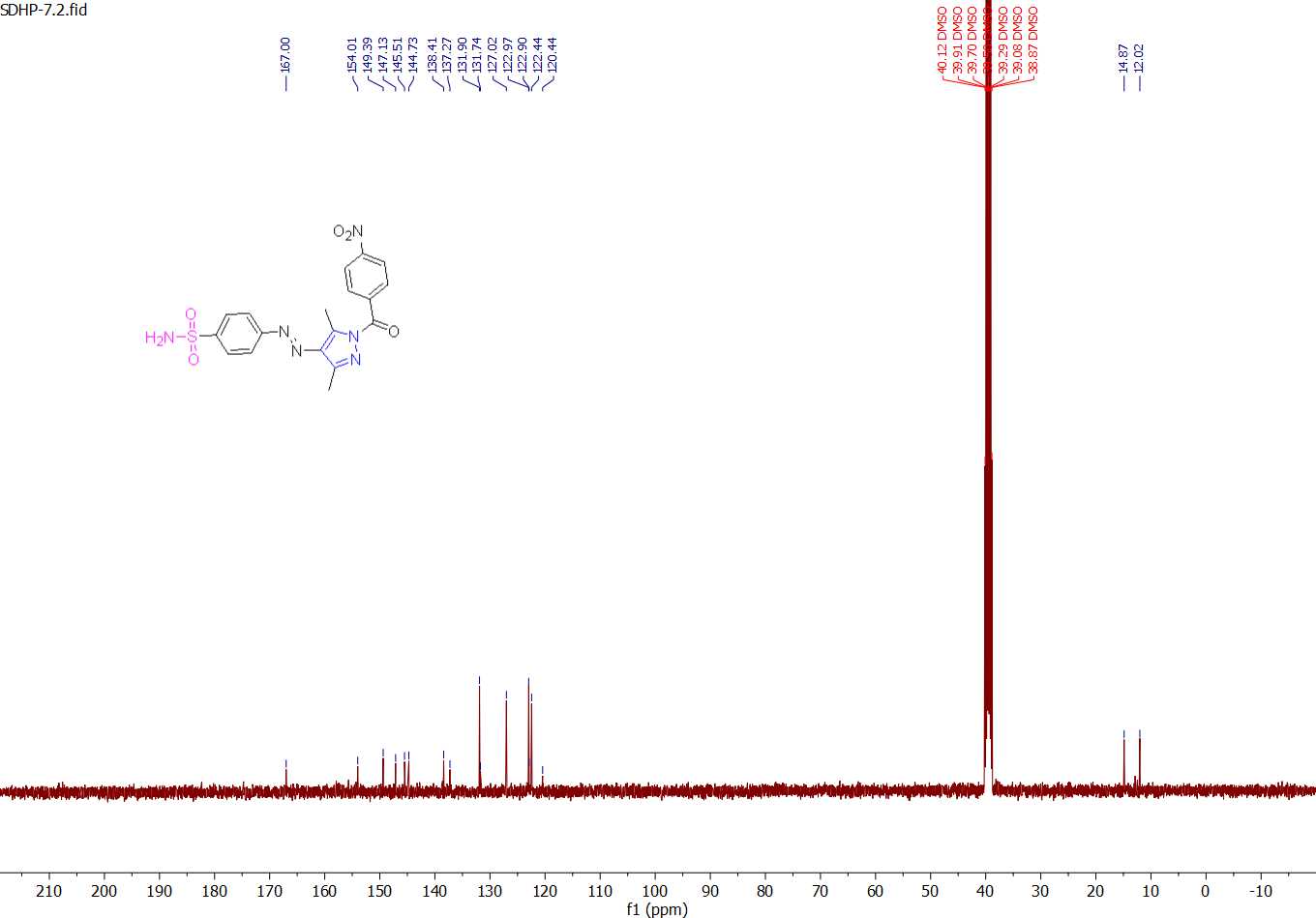


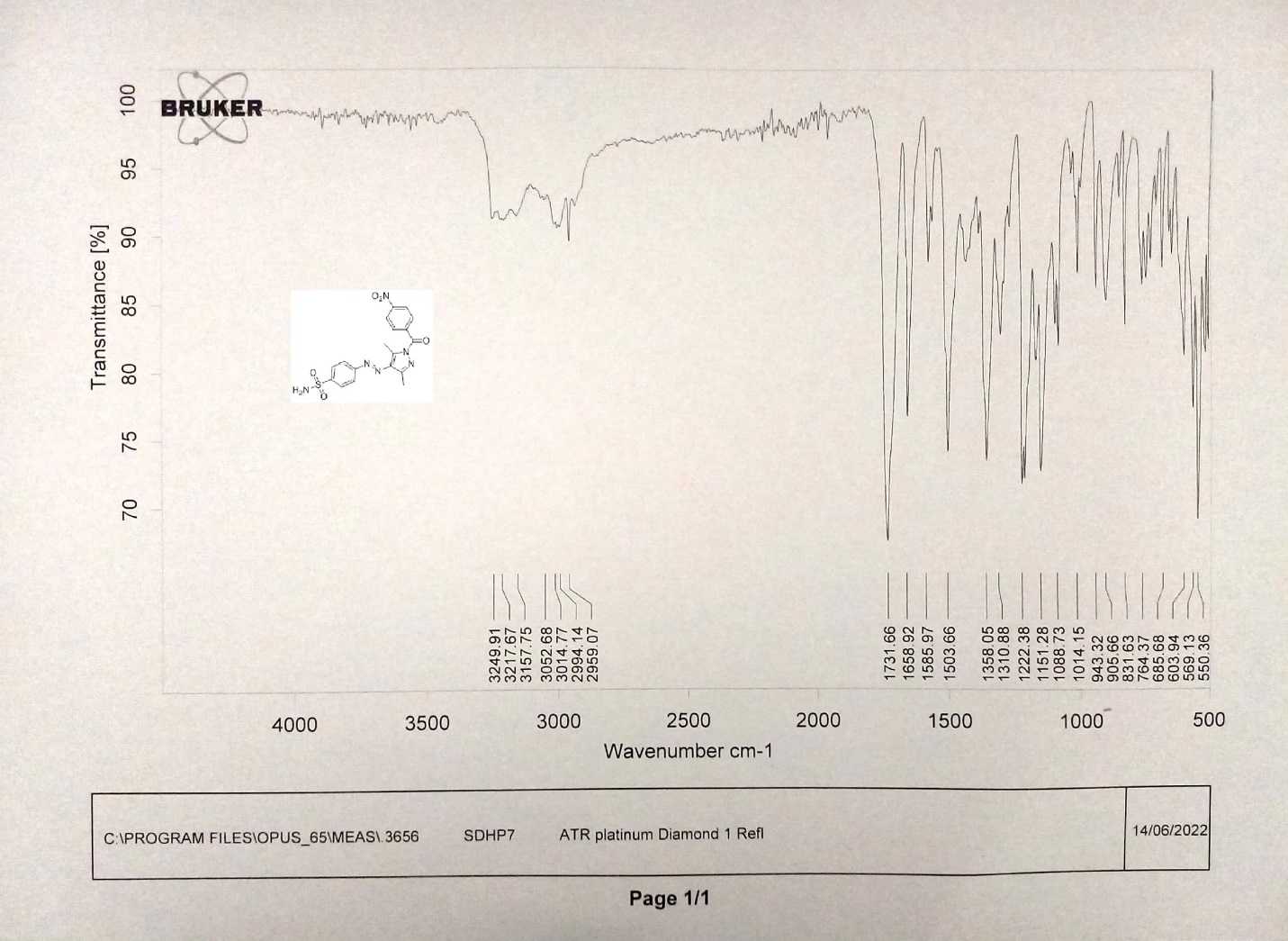


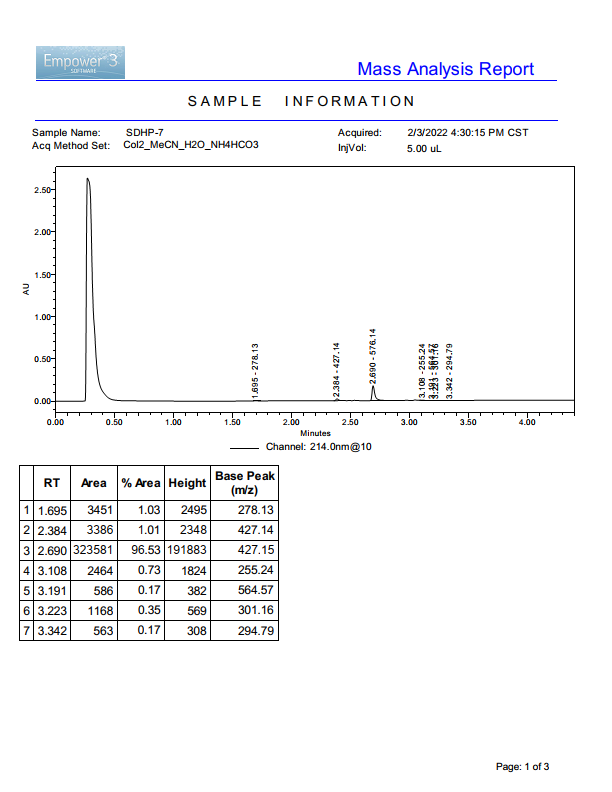


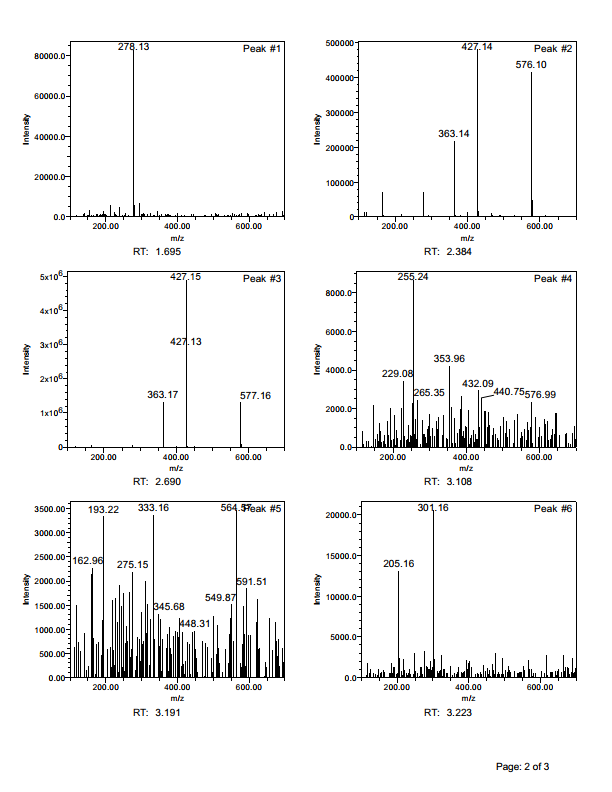


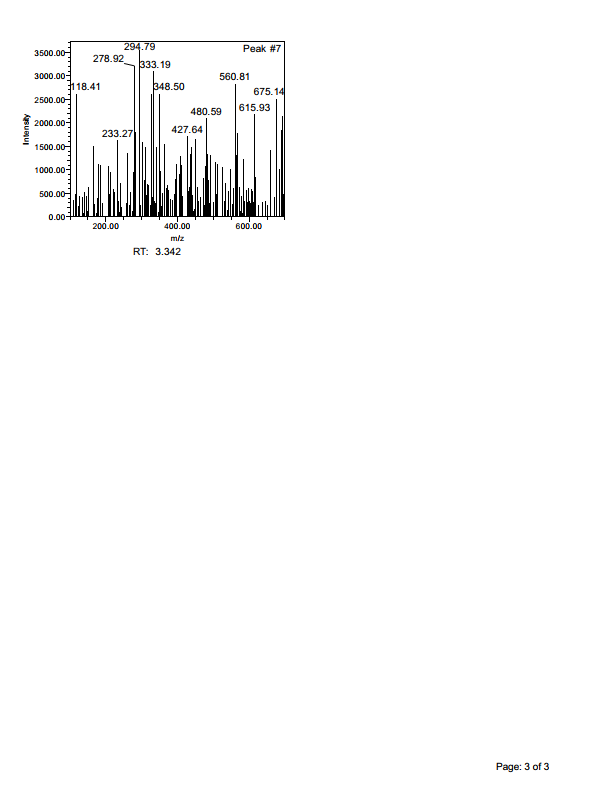


*(E)-4-((3,5-Dimethyl-1-(3-nitrobenzoyl)-1H-pyrazol-4-yl)diazenyl)benzenesulfonamide (****f****)*


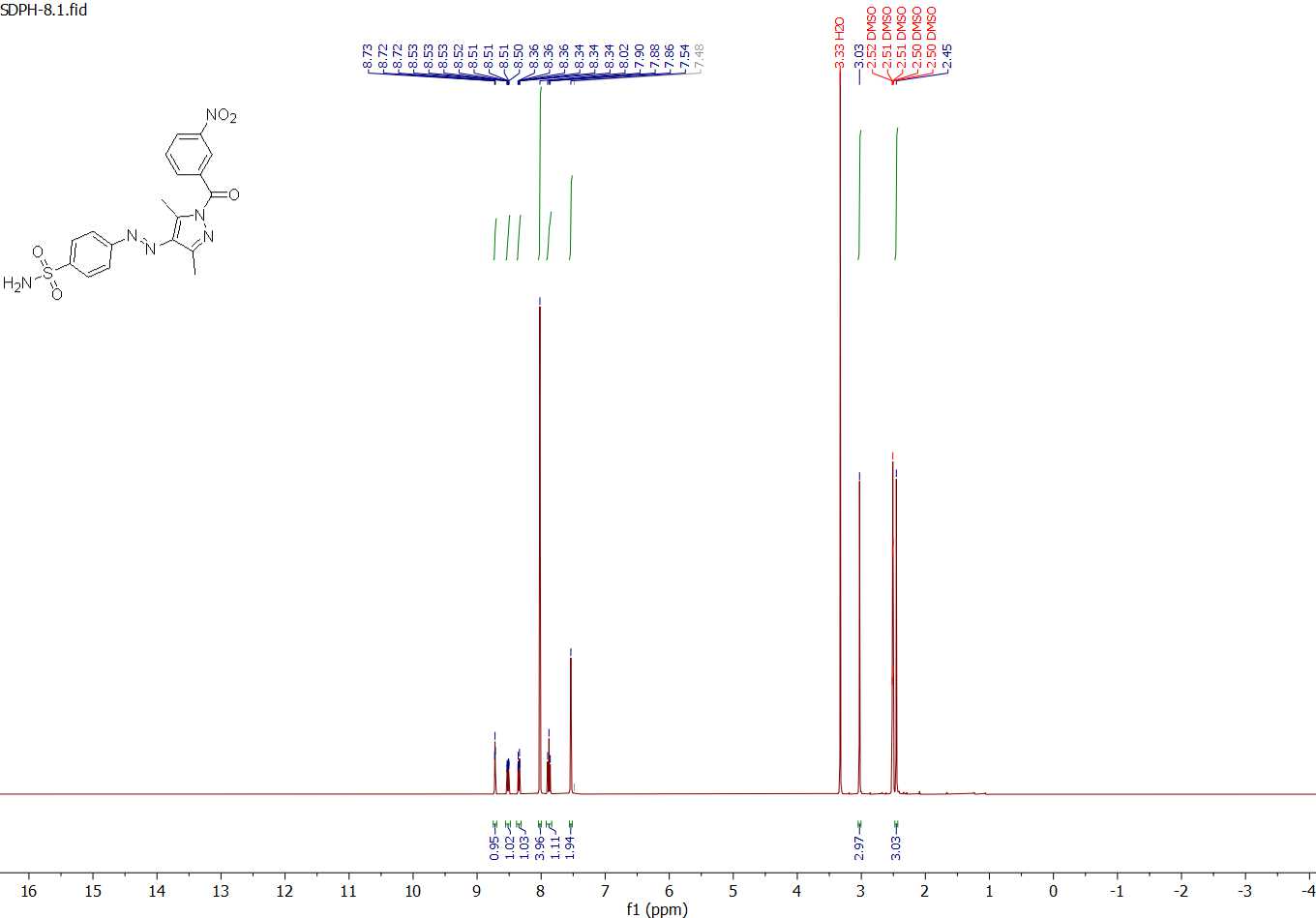

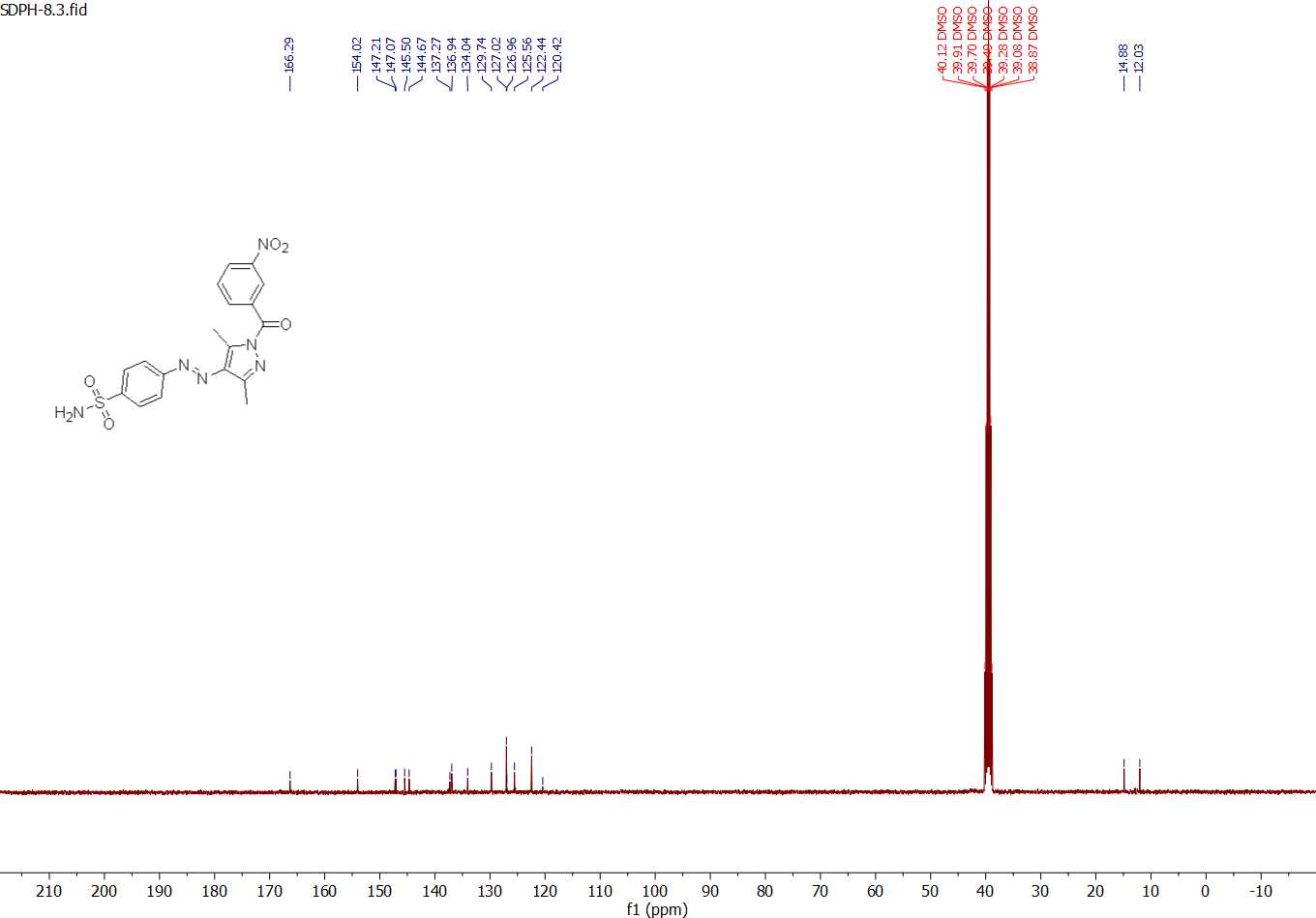


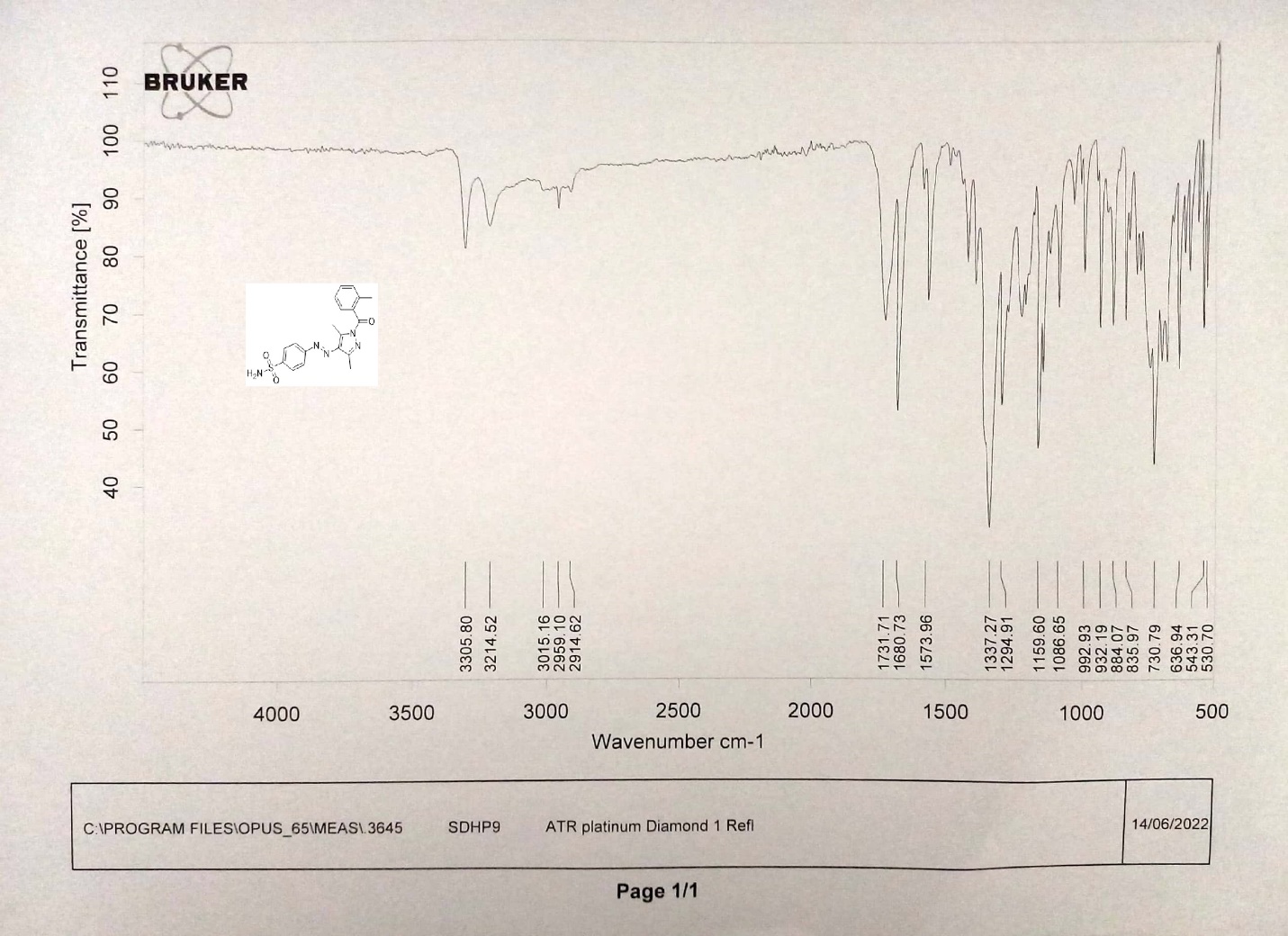


*(E)-4-((3,5-Dimethyl-1-(2-methylbenzoyl)-1H-pyrazol-4-yl)diazenyl)benzenesulfonamide (****5g*)**


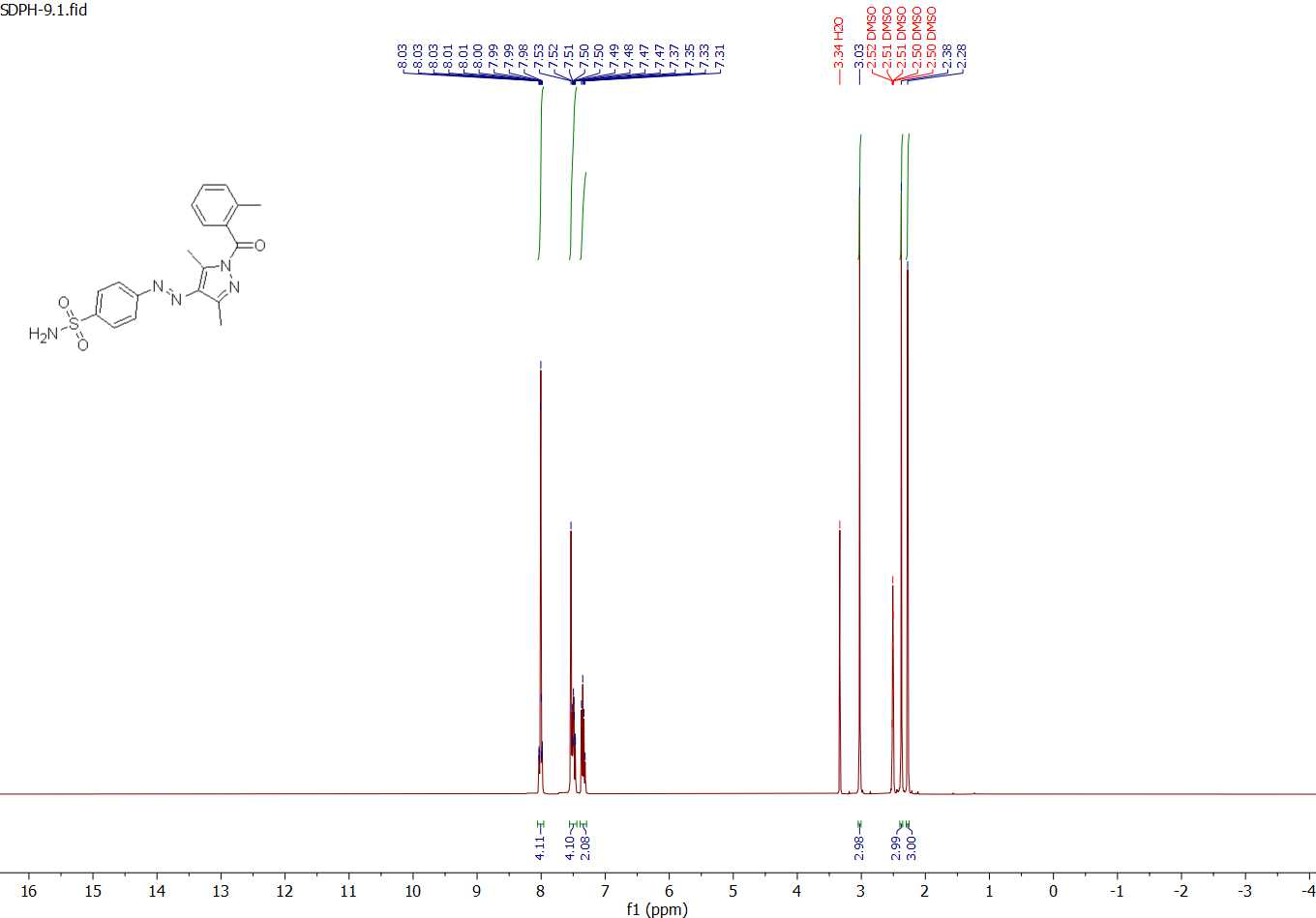

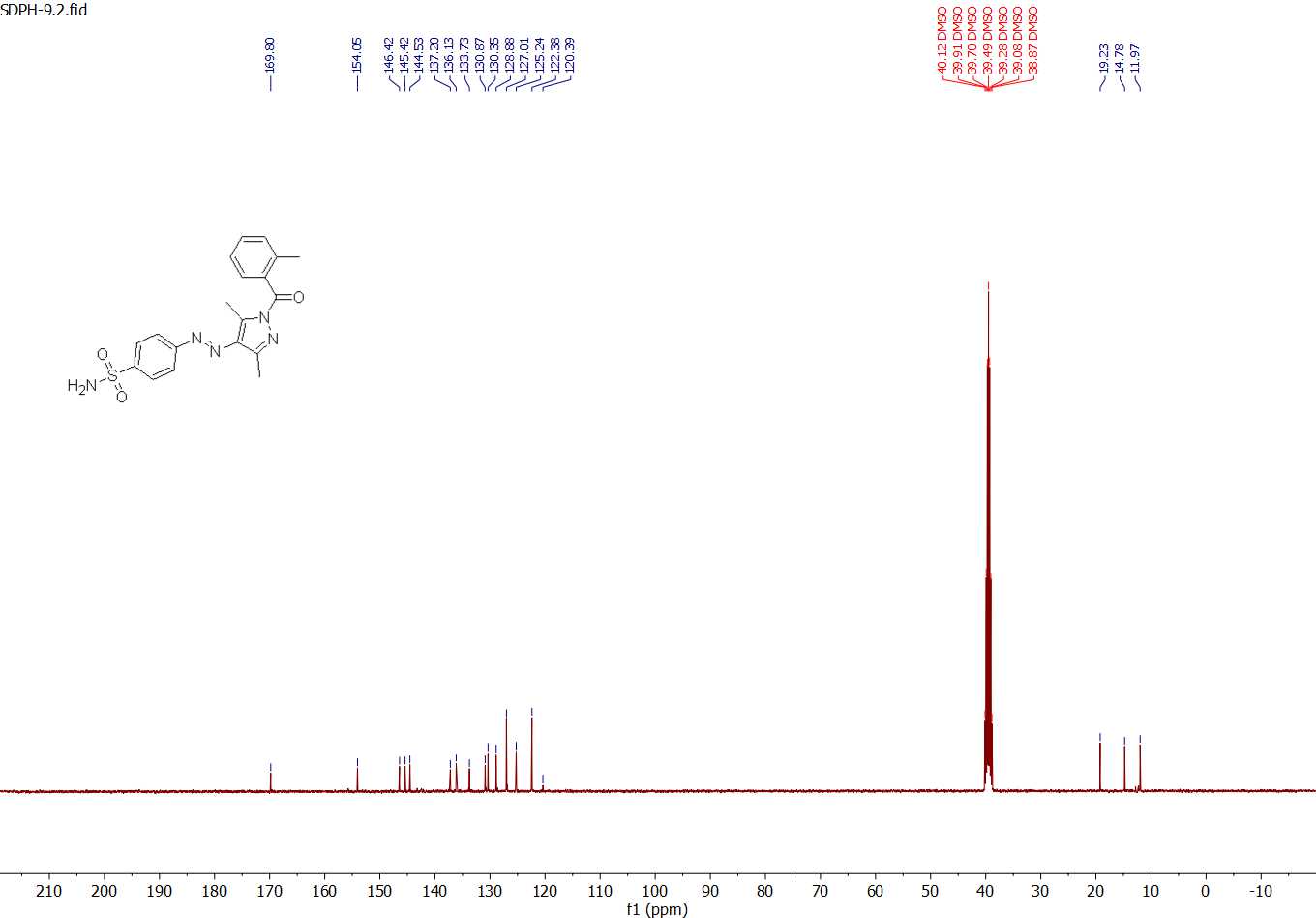


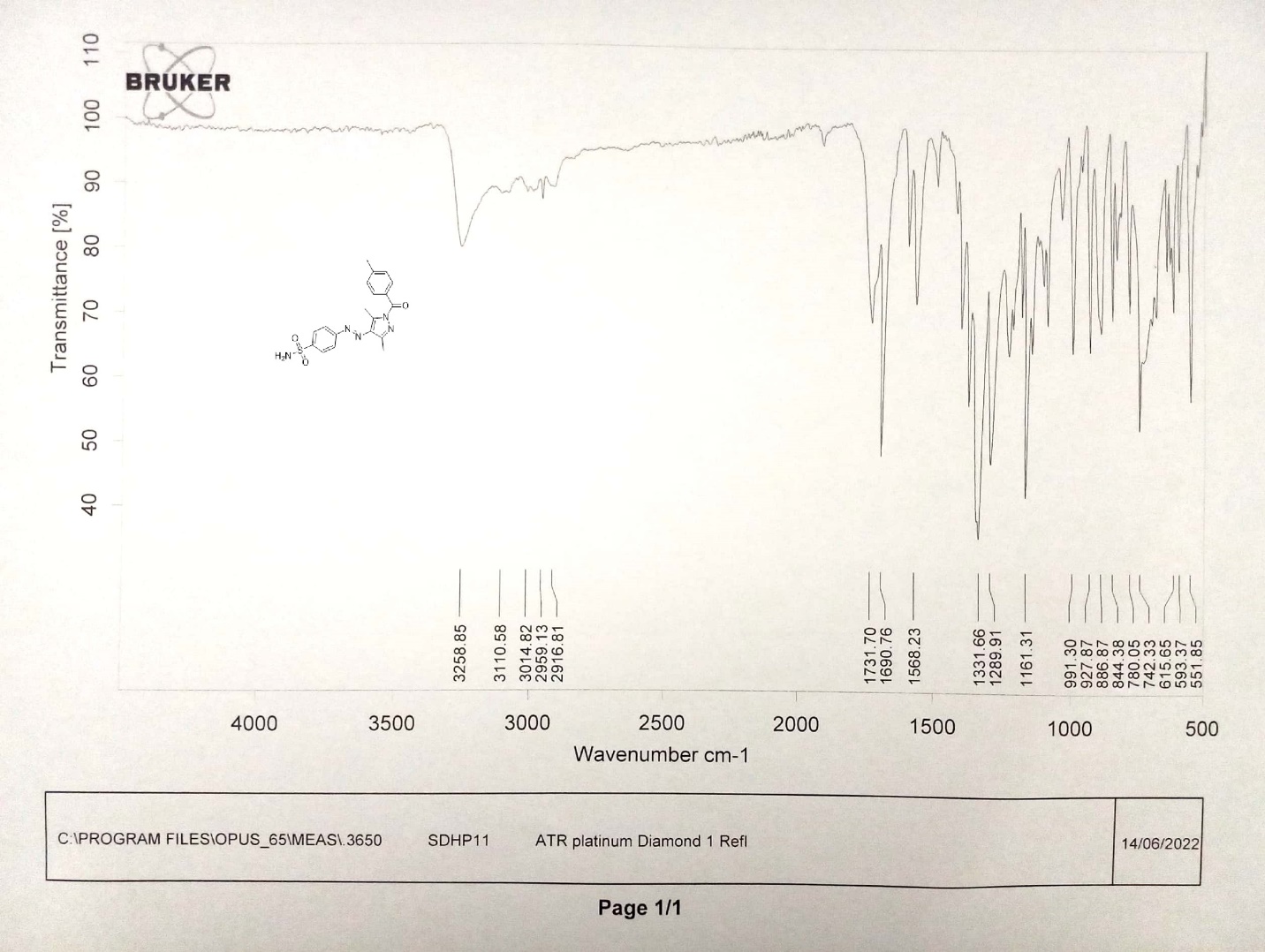


*(E)-4-((3,5-Dimethyl-1-(4-methylbenzoyl)-1H-pyrazol-4-yl)diazenyl)benzenesulfonamide (****5h****)*


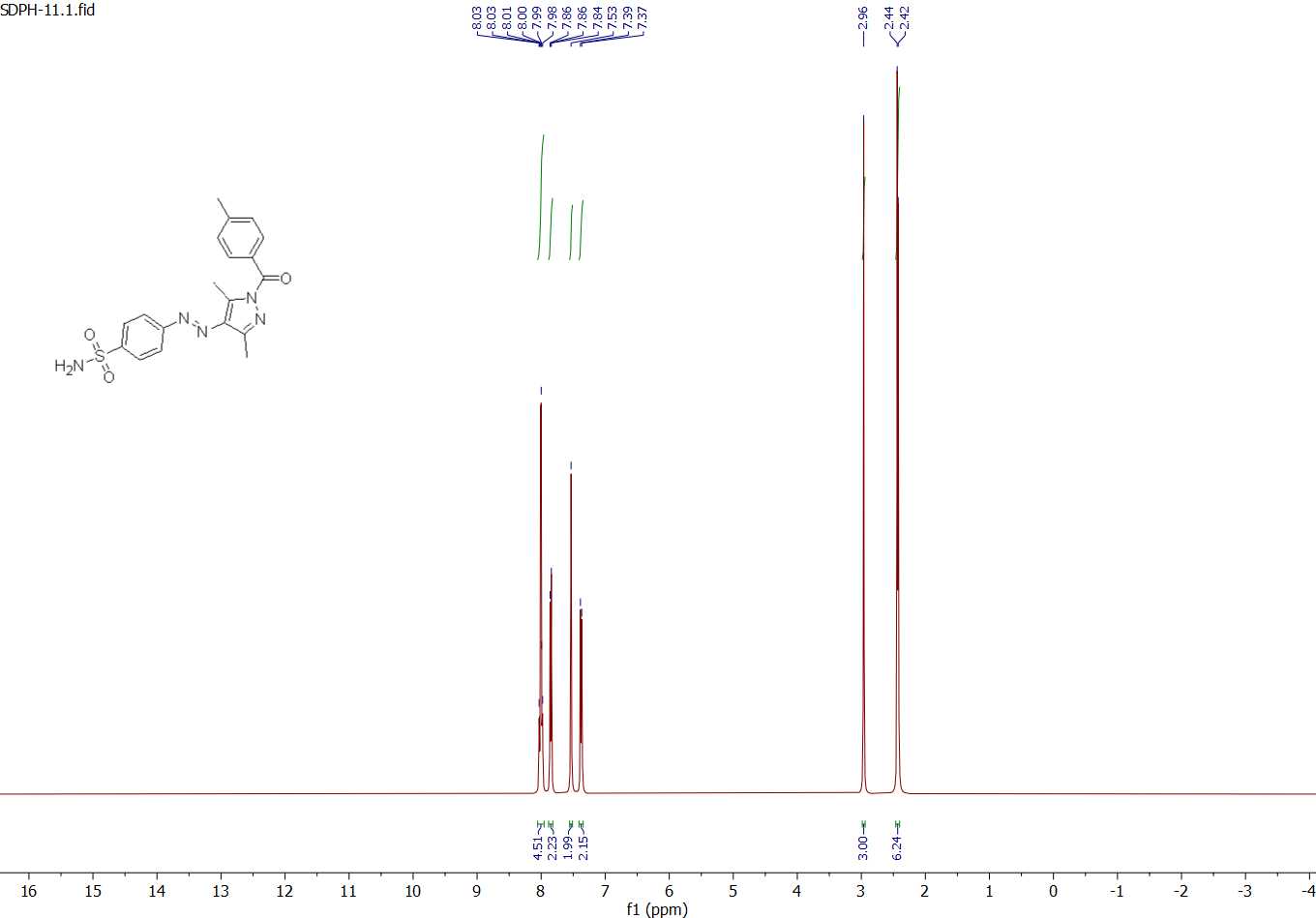

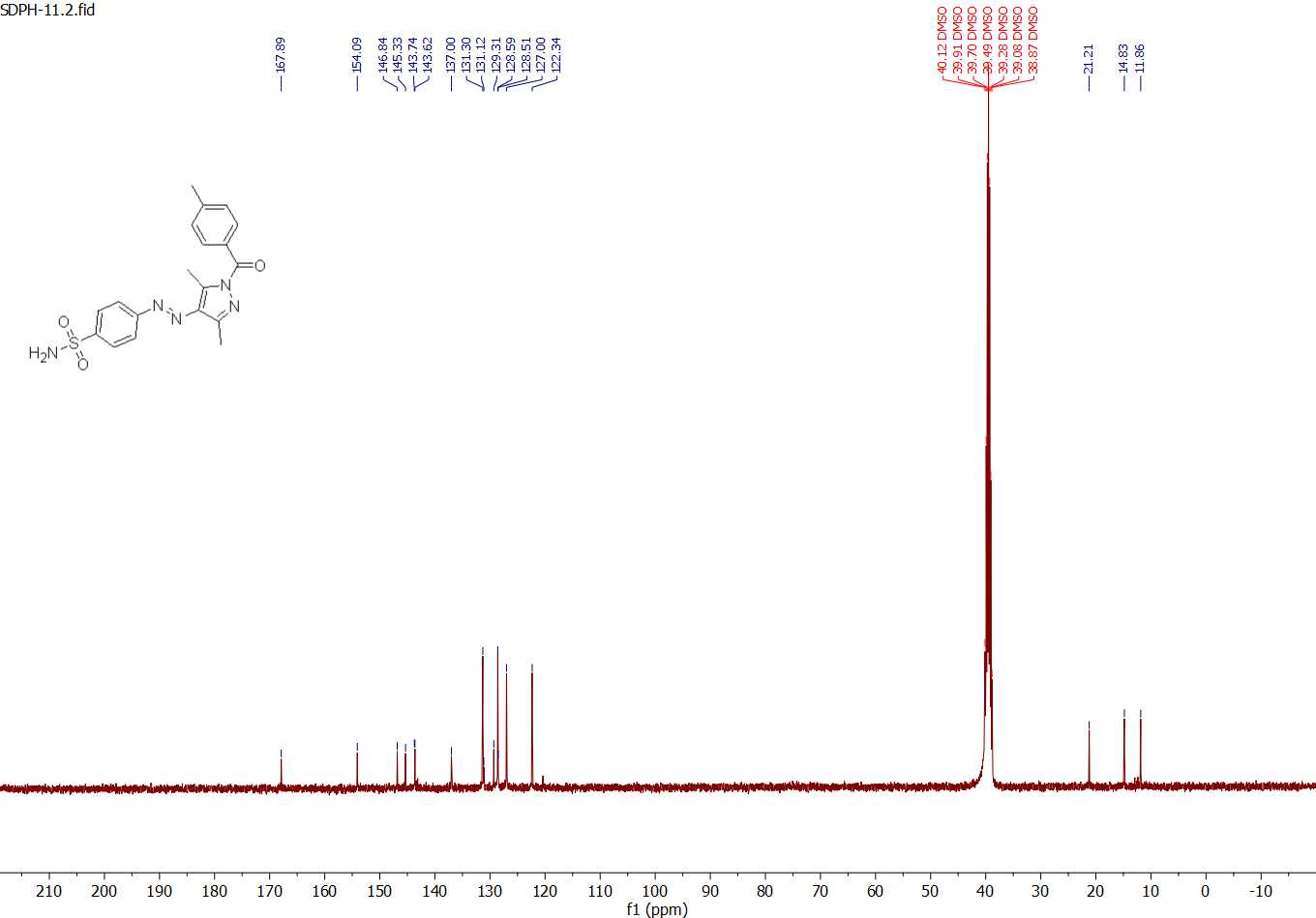


*(E)-4-((1-(4-Bromobenzoyl)-3,5-dimethyl-1H-pyrazol-4-yl)diazenyl)benzenesulfonamide (****i****)*


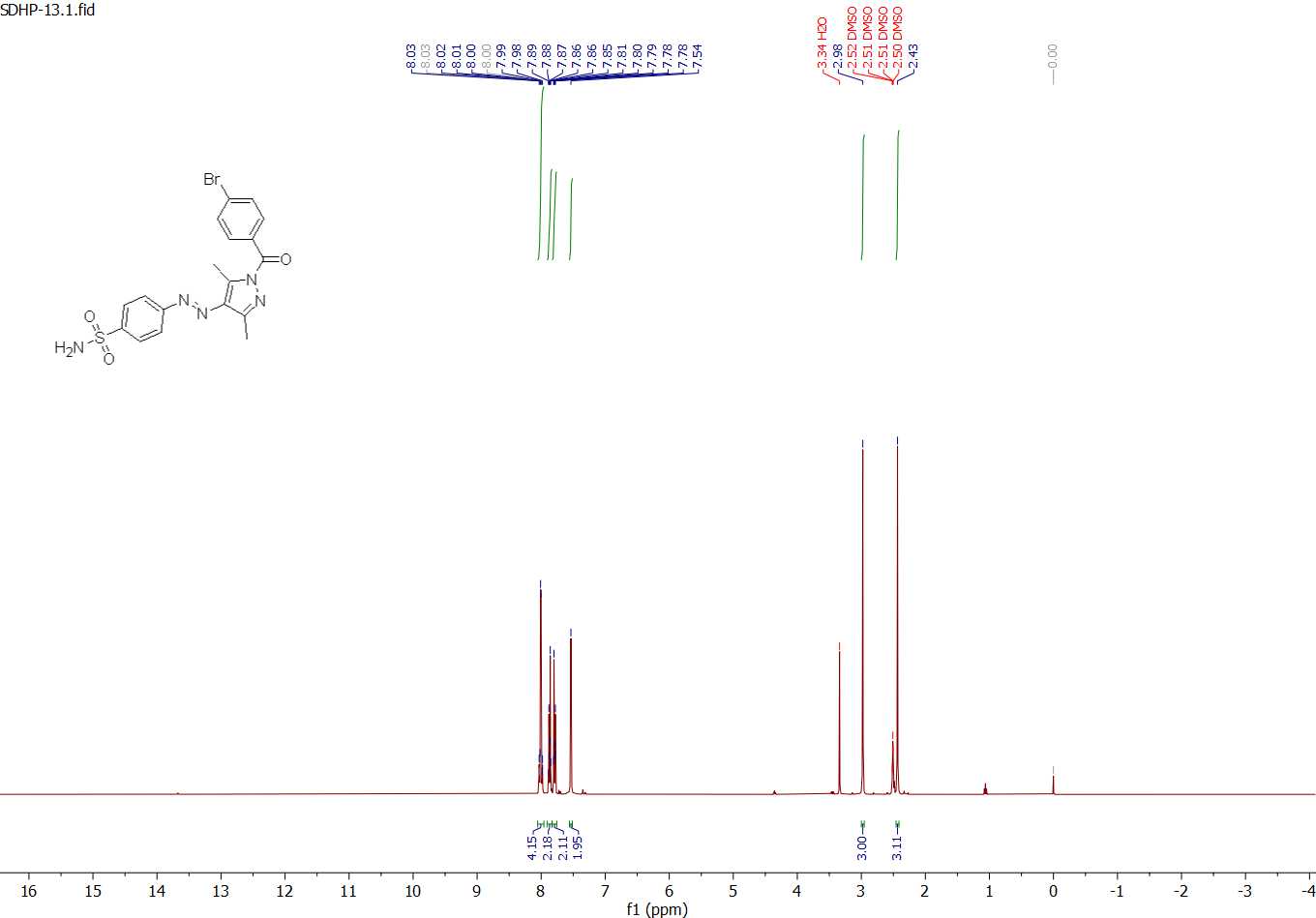

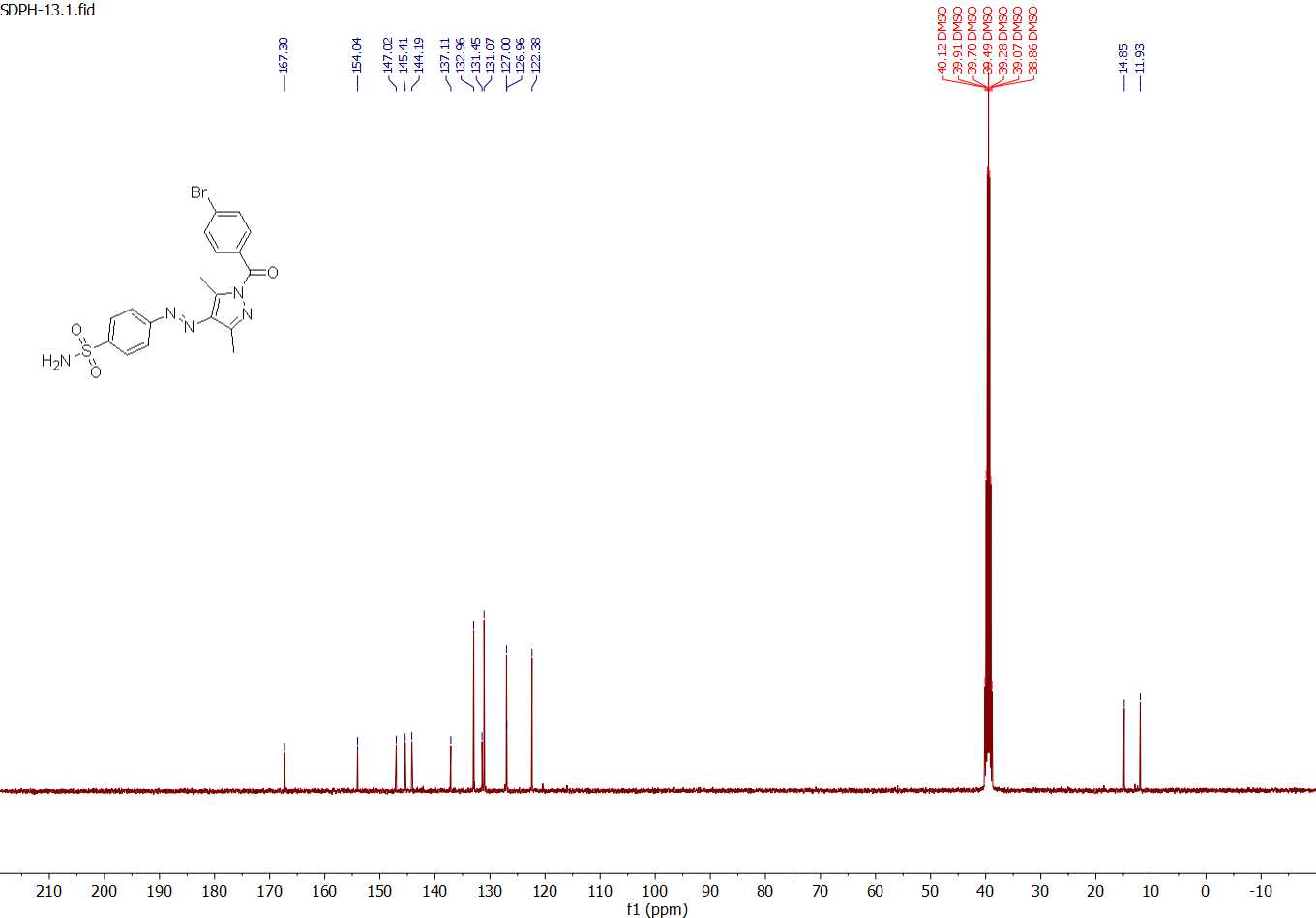


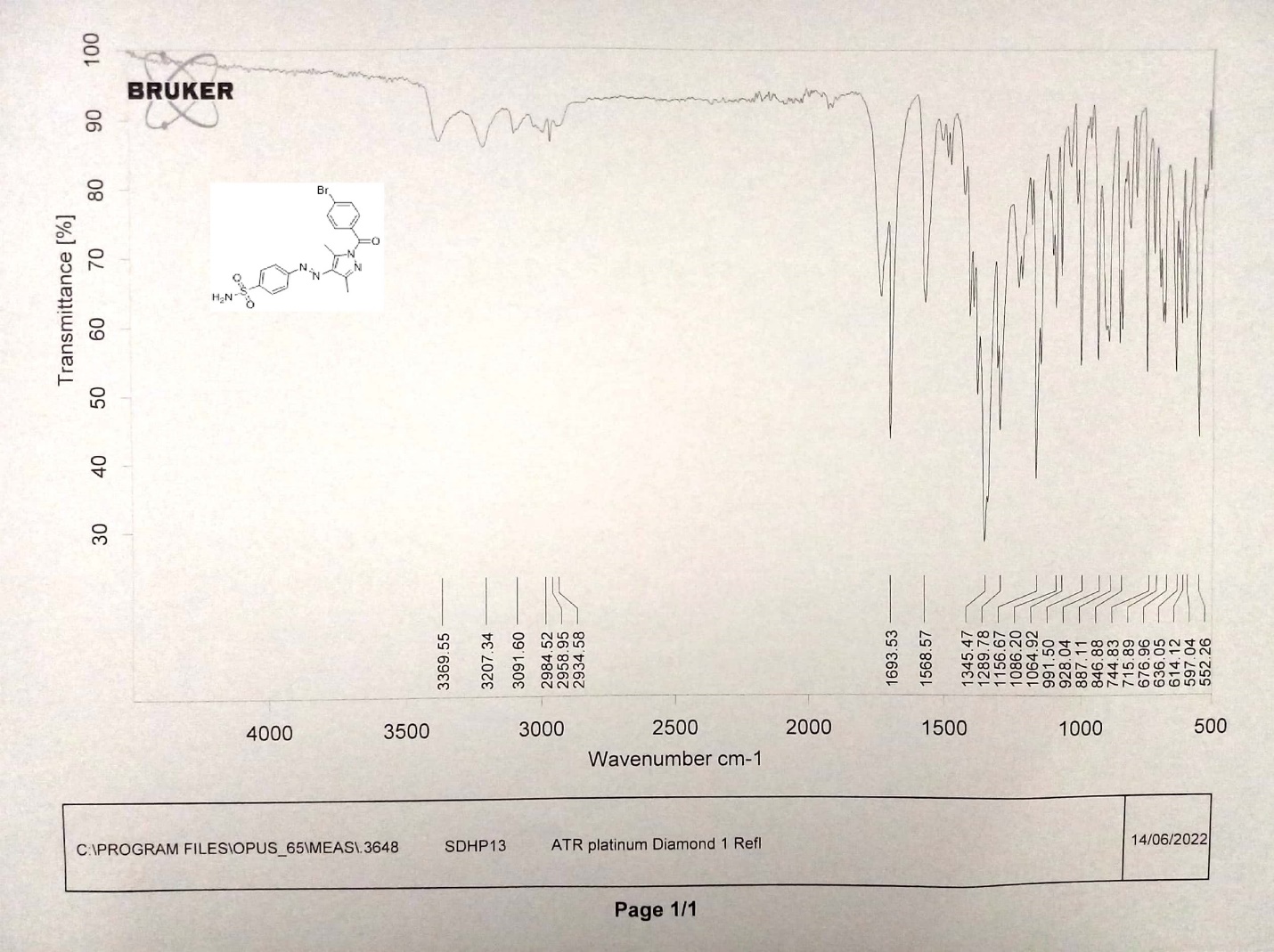


*(E)-4-((1-(3-Methoxybenzoyl)-3,5-dimethyl-1H-pyrazol-4-yl)diazenyl)benzenesulfonamide (****j****)*


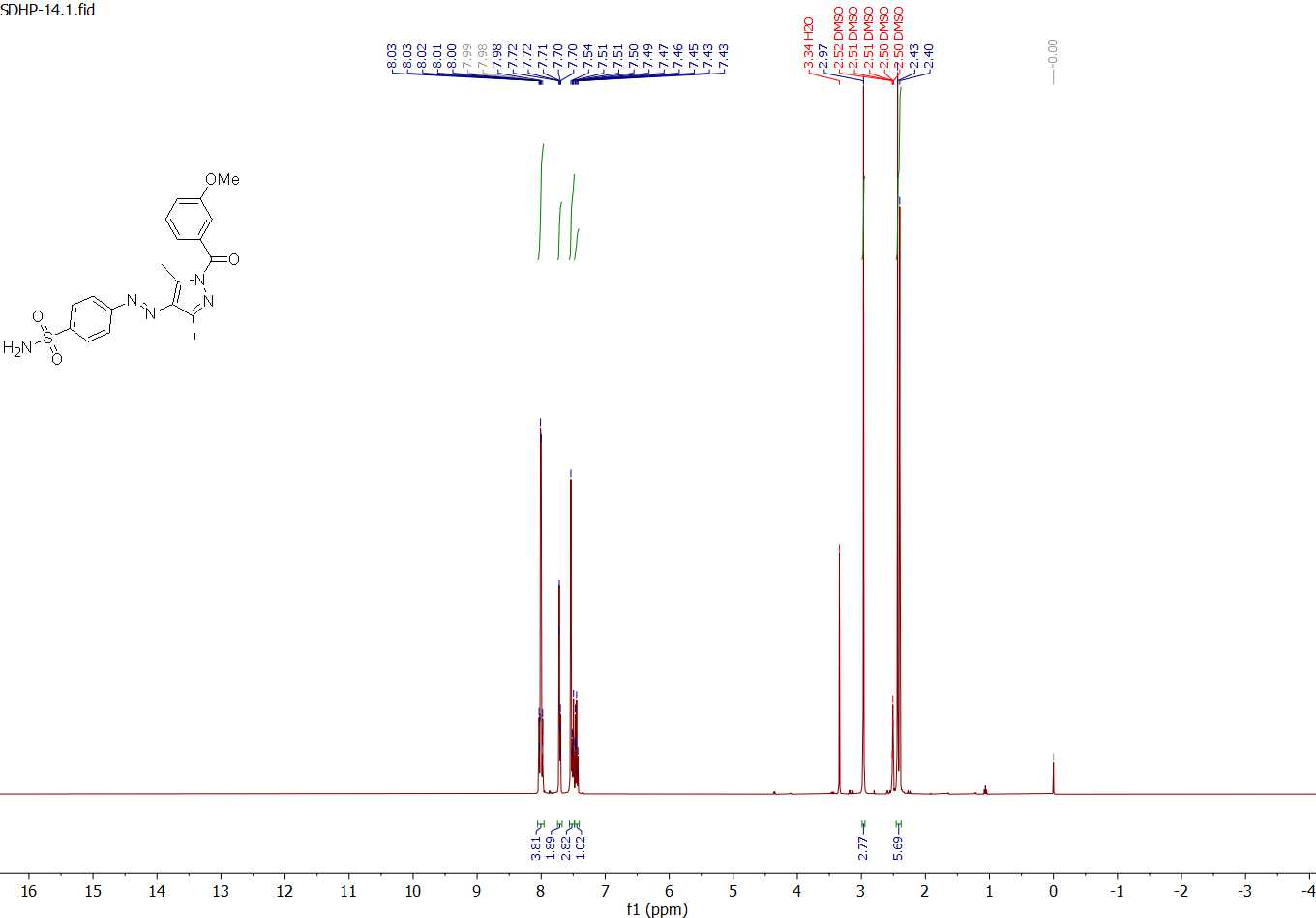

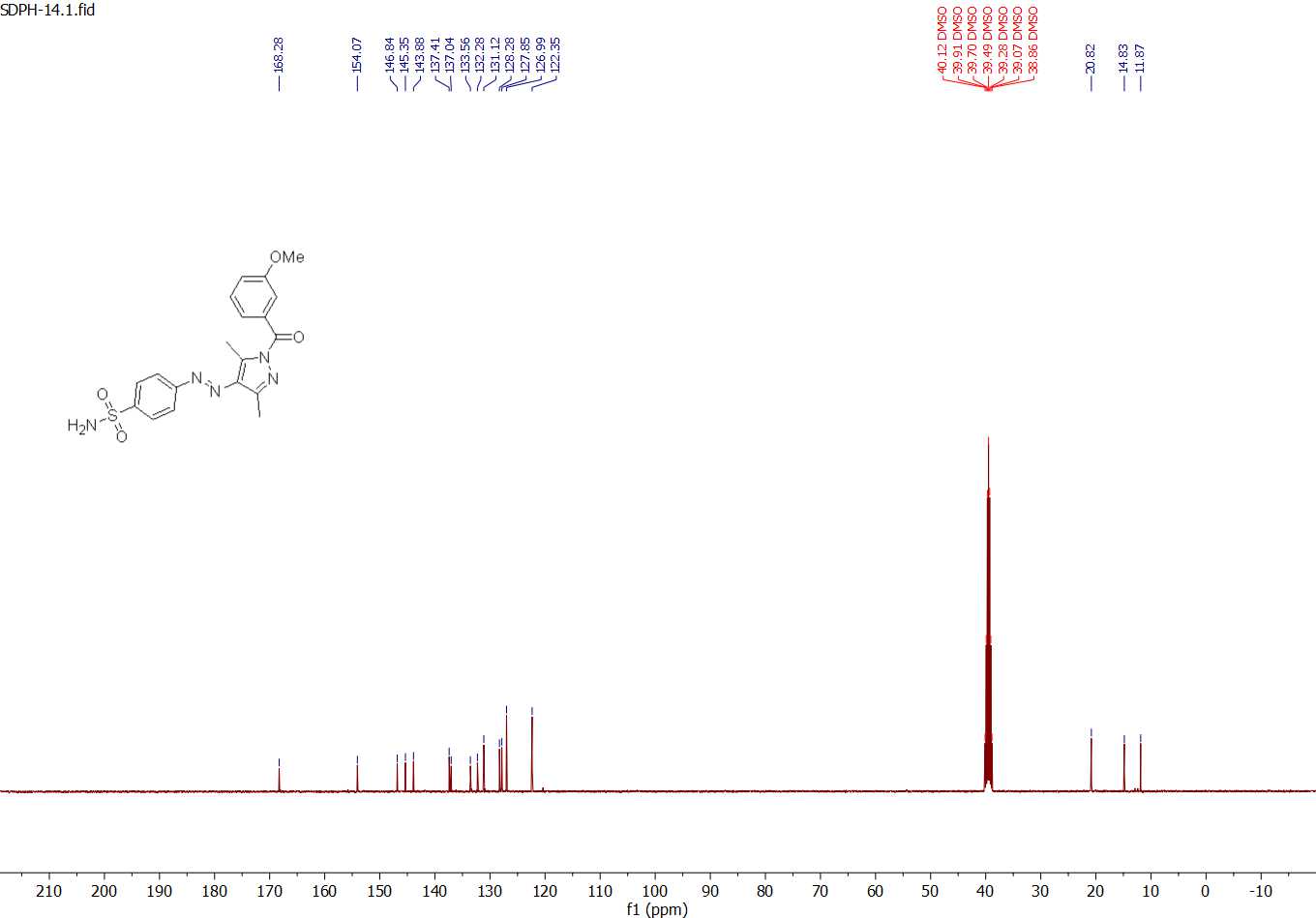


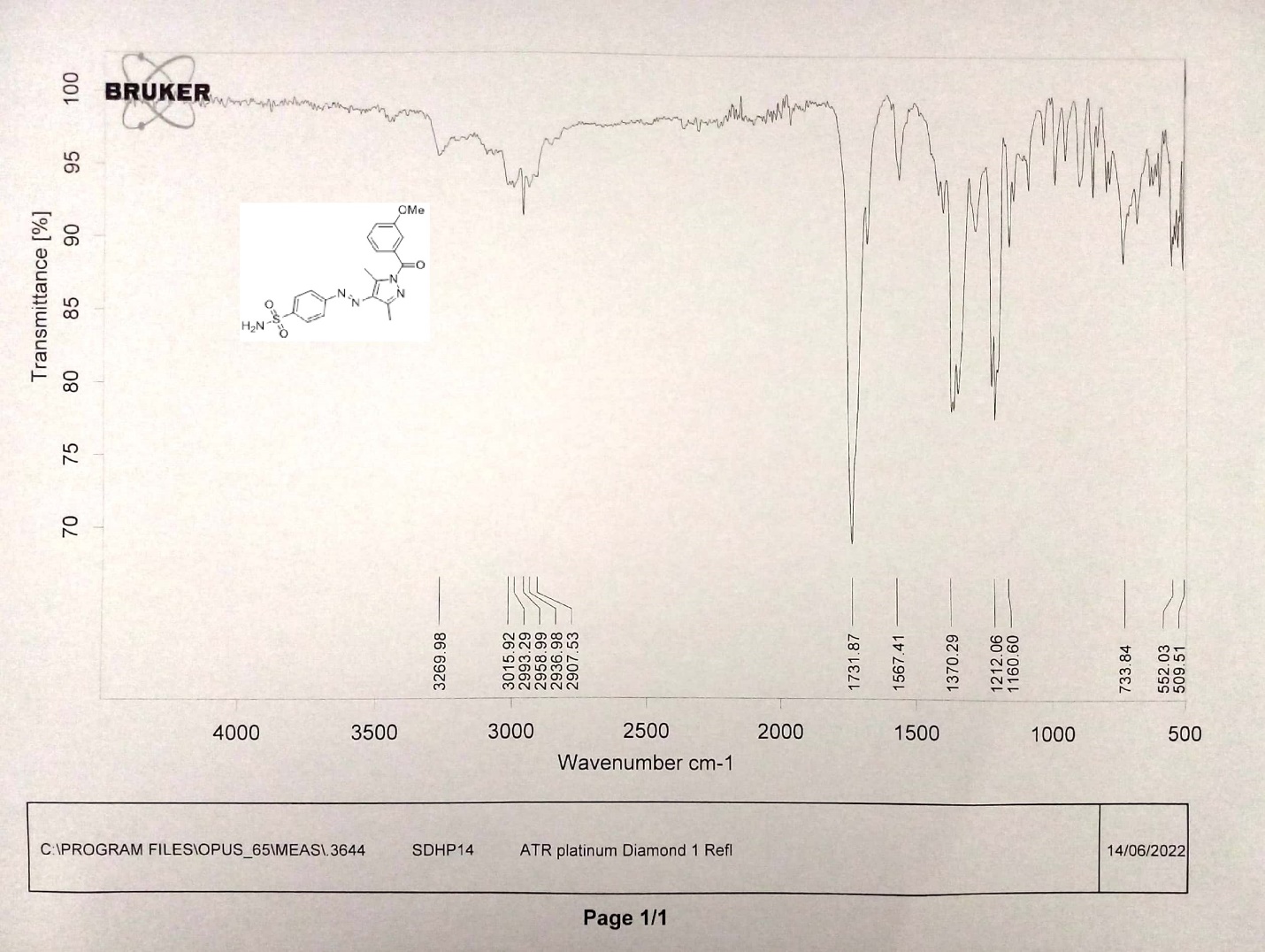


*(E)-4-((3,5-Dimethyl-1H-pyrazol-4-yl)diazenyl)benzenesulfonamide (****5k****)*


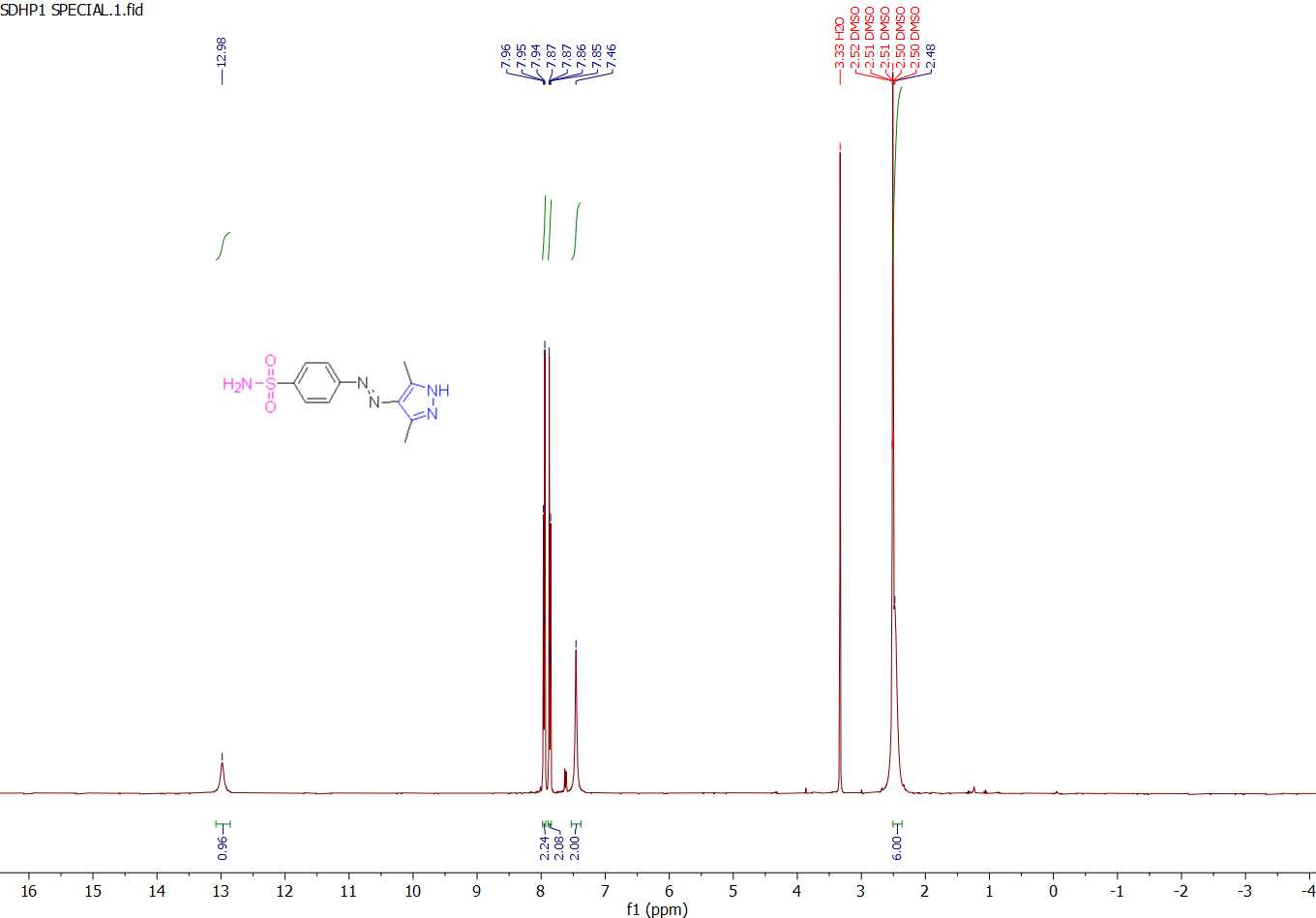

Supplement: Supplementary file 1 [file DataSheet1.docx]
